# Epigenetic map and genetic map basis of complex traits in cassava population

**DOI:** 10.1038/srep41232

**Published:** 2017-01-25

**Authors:** Meiling Zou, Cheng Lu, Shengkui Zhang, Qing Chen, Xianglai Sun, Pingan Ma, Meizhen Hu, Ming Peng, Zilong Ma, Xin Chen, Xincheng Zhou, Haiyan Wang, Subin Feng, Kaixin Fang, Hairong Xie, Zaiyun Li, Kede Liu, Qiongyao Qin, Jinli Pei, Shujuan Wang, Kun Pan, Wenbin Hu, Binxiao Feng, Dayong Fan, Bin Zhou, Chunling Wu, Ming Su, Zhiqiang Xia, Kaimian Li, Wenquan Wang

**Affiliations:** 1 Huazhong Agricultural University, Wuhan, China; 2 The Institute of Tropical Biosciences and Biotechnology, Chinese Academy of Tropical Agriculture Sciences, Haikou, China; 3 Guangxi Academy of Agricultural Sciences, Guilin, China

## Abstract

Cassava (*Manihot esculenta* Crantz) is an important tropical starchy root crop that is adapted to drought but extremely cold sensitive. A cold-tolerant, high-quality, and robust supply of cassava is urgently needed. Here, we clarify genome-wide distribution and classification of CCGG hemi-methylation and full-methylation, and detected 77 much candidate QTLs^epi^ for cold stress and 103 much candidate QTLs^epi^ for storage root quality and yield in 186 cassava population, generated by crossing two non-inbred lines with female parent KU50 and male parent SC124 (KS population). We developed amplified-fragment single nucleotide polymorphism and methylation (AFSM) genetic map in this population. We also constructed the AFSM QTL map, identified 260 much candidate QTL genes for cold stress and 301 much candidate QTL genes for storage root quality and yield, based on the years greenhouse and field trials. This may accounted for a significant amount of the variation in the key traits controlling cold tolerance and the high quality and yield of cassava.

The majority of studies on many species are based on the complex traits^1^,^2^,^3^. Even though an increasing number of studies are investigating the heritable phenotypic variation in the model plants, such as *Arabidopsis thaliana*^2^,^4^,^5^ and soybean^6^, the heritable variations in cytosine methylation in non-model crops have not been investigated^7^,^8^. Cytosine methylation is a DNA base modification involved in the development, disease, and silencing of transposable elements and genes^9^. CG methylation is commonly found within gene bodies in plants^10^. Intraspecific surveys have revealed widespread variations in DNA methylation patterns within populations^11^,^12^. A key challenge in the field of population genetics is showing the changes in the genome-wide CCGG hemi-methylation and full-methylation heritable variation patterns and SNVs (single-nucleotide polymorphisms and indels) associated with heritable phenotypic variation in populations with high heterozygosity and large genomes.

To address these difficulties, we established in a cassava population with a highly heterozygous and large genome^13^, and measured eight complex traits through field and greenhouse experiments. Cassava (*Manihot esculenta* Crantz), a starchy root crop, is a staple food and animal feed and serves as an important source of bioethanol^14^. As a tropical root crop, cassava is sensitive to cold. At temperatures less than 10 °C, cassava undergo chilling-injury or death, including delayed sprouting of the stem cutting, decreased yield, reduced leaf expansion and even leaf necrosis^15^. Thus, chilling injury is the most important factor limiting cassava’s geographic distribution and productivity. The cold tolerance of cassava is very important for protection of the storage roots and propagation stems^15^. Cold tolerance, high quality, and a robust supply of cassava are urgently in demand. SC124 exhibits a high yield and cold tolerance but low proportion of starch. In contrast, KU50 has many elite economic traits, such as a high proportion of starch and the ability to produce high yields, but is intolerant of cold temperatures. In this study, we established a cold-tolerance cassava mapping population (KS) that descended from a cross between the two non-inbred lines, with KU50 as the female parent and SC124 as the male parent. We then used an amplified-fragment single nucleotide polymorphism and methylation approach (AFSM)^16^ to concurrently identify whole genome hemi-methylation and full-methylation heritable variation pattern and SNVs in 186 cassavas from the KS population and performed deep sequencing on the transcriptomes of the parents to assess the function of the differentially methylated regions (DMRs), and to clarify the relationship between them and differentially expressed genes in cassava. Moreover, these AFSM markers were used to generate a genetic linkage map and identify repeatable quantitative trait loci (QTL) and epigenetic quantitative trait loci (QTL^epi^) based on years of repeated experiments in the greenhouse and field.

## Result and Discussion

### DNA methylation landscape and cytosine methylation patterns in cassava genome

**Figure 1:**
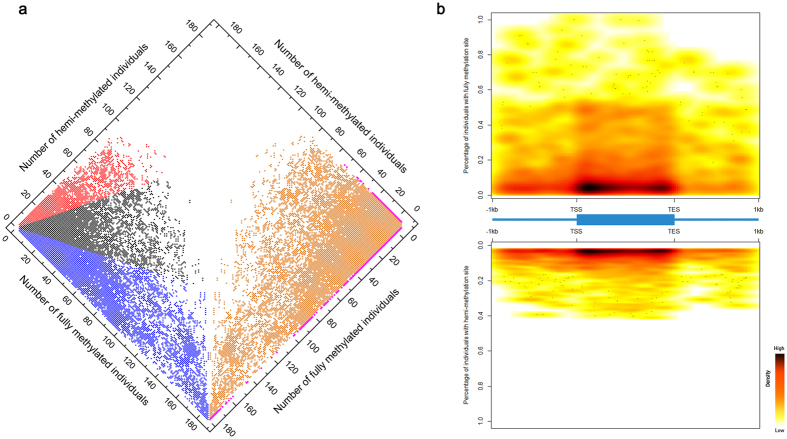
DNA Methylation Landscape in Cassava Genome. (**a**) Graphical representation of the pattern of methylated sites in cassava KS population. The y-axis and x-axis indicate the number of individuals, which were detected to have either hemi-methylated or fully methylated sites in the KS cassava population. The left region indicates the distribution of hemi-methylated or fully methylated sites. The red circles represent the hemi-methylated sites (hemi-methylated sites > 2/3), while the blue circles depict the fully methylated sites (fully methylated sites > 2/3). The right region indicates the distribution of DNA methylation polymorphisms for the cassava KS population. The orange circles represent the polymorphic methylated sites in the population, while the pink circles represent the monomorphic methylated sites. (**b**) Distribution of the density of fully methylation (up) and hemi-methylation (down) in cassava KS population across gene bodies (from the start of 5′ UTR to the end of the 3′ UTR, including 2000 bp up- and downstream), using heatmap (the red and yellow color represent the density).

A total of 122,466,322 reads from the cassava KS populations, which covered 32.3 Mb, or 8.43% of the cassava genome, were aligned to the KU50 cassava genomic DNA sequences^14^ using Bowtie2^17^. Among these reads, 17,590,268 reads were covered 12.65% of the cassava gene body and promoter (2,000-bp upstream) regions. The average coverage of the cassava genome for each sample was 5-fold. A total of 2,918,057 reads were mapped for the female parent KU50 and 2,785,375 reads were mapped for the male parent SC124. Out of the 247,737 total methylated sites, 103,641 polymorphic methylated sites (41.84%) were identified in the parents and F1 individuals using the AFSM approach (Fig. 1a, right). The 73,212 filtered CCGG-methylated sites (hemi-methylated or fully methylated sites, >2/3) included 17,555 hemi-methylated and 55,657 fully methylated sites (Fig. 1a, left). The total full-methylation density was higher than the hemi-methylation, especial enriched in gene-body (Fig. 1b). The CmCGG fully methylation detected in this study was higher than the mCCGG hemi-methylation and mCCGG fully methylations (49.35% Vs. 26.27% and 24.38% for CmCGG fully methylation, mCCGG hemi-methylation and mCCGG fully methylation, respectively), especially in gene regions (51.66% Vs. 25.27% and 23.07% for CmCGG fully methylation, mCCGG hemi-methylation and mCCGG fully methylation, respectively) in cassava (Fig. S2).

These 119,285 methylated sites in both the parents and F1 individuals could be grouped into twelve classes (classes A1-2, B1-4, C1-2 and D1-4, as shown in Tables S1 and S2). Classes D2-4 contained the most methylated sites, including 62,669 methylated sites exhibiting differing methylation patterns in the F1 progeny and 48,007 methylated sites exhibiting disparate methylation patterns between the parents. Interestingly, we did not observe Classes B1-4 and C1-2 in cassava. One possible source of heritable methylation patterns could be selection for species survival. In addition, heritable methylation patterns can also be affected by SNPs^18^.

### Methylation diversity in population

The observation that monomorphic fully methylation in population, biased the distribution toward the promoter and transcribed regions (Fig. 1b) may indicate that monomorphic full-methylation is associated with the maintenance of a higher species fidelity, including cell parts, basic cellular and metabolic processes, catalytic activity and binding function (Fig. S1 and Table S3). On the contrary, we observed that polymorphic methylation, especially polymorphic fully methylation, tended to exhibit enrichment in gene bodies in population (Fig. 1b).

### Effects of DNA methylation on gene expression

**Figure 2:**
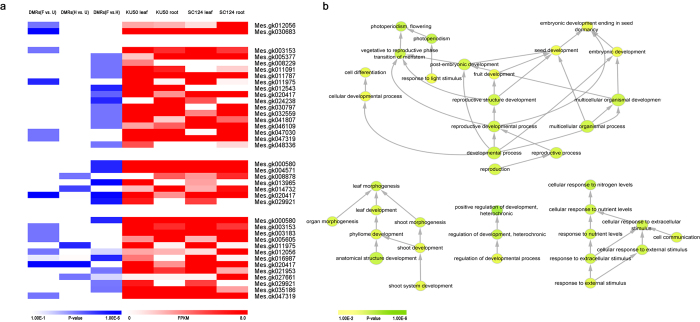
Differentially methylated and differentially expressed genes in Cassava. (**a**) A heatmap representation of genes with differential methylation and differentially expressed (DMR-DEGs) between the leaf and root in SC124 and KU50, based on a cuffdiff pairwise analysis (false discovery rate [FDR] < 0.001). The related genes were categorized; (**b**) Part of functional category enrichment was calculated using BiNGO^19^ (P < 0.001, χ^2^ text) for these DMR-DEGs relative to the KU50 genes^14^.

To assess the function of the differentially methylated regions (DMRs) in cassava, and to clarify the relationship between them and differentially expressed genes, we constructed RNA-seq libraries and performed deep sequencing on the transcriptome of the parents’ roots and leaves belonging to the KS population as well as AFSM sequencing. A total of 130,707,837 high-quality (filtered) reads were generated (including 29,905,212 reads for the KU50 leaf, 34,029,215 reads for the KU50 root, 33,174,195 reads for the SC124 leaf, and 33,599,215 reads for the SC124 root). A total of 1,482 DMRs (SC124 leaf vs. KU50 leaf, SC124 root vs. KU50 root, leaf vs. root in KU50, and leaf vs. root in SC124) were detected in gene bodies and promoters (Fig. S4). Among these, 38 DMRs were considered significantly differentially expressed (DMR-DEGs) based on a cuffdiff pairwise analysis (false discovery rate (FDR) < 0.001) (Fig. 2). The functional category enrichment was then assessed using BiNGO^19^ (P < 0.001, χ^2^ text) for these DMR-DEGs relative to the KU50 genes^14^. These DMR-DEGs tended to exhibit enrichment of cellular components, including small nucleolar ribonucleoprotein complex and intracellular membrane-bounded organelle. Enriched molecular function for DMR-DEGs included translation factor activity, nucleic acid binding, ATPase activity. For biological processes, organ morphogenesis, cell differentiation, response to light stimulus and cellular response to extracellular stimulus (Table S4, Fig. 2b).

### QTLs^epi^ mapping and relative genes involved in cold tolerance and yielding

**Figure 3:**
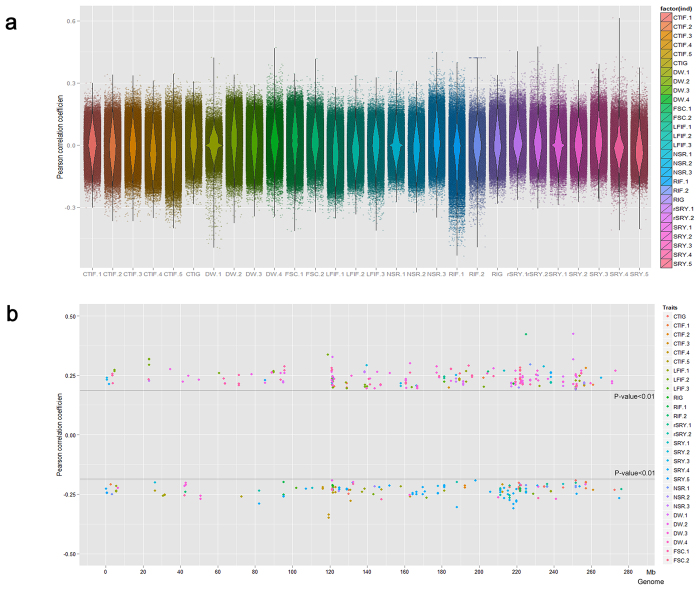
QTL^epi^ mapping profiles in KS population. (**a**) As a violin plot shows the distribution of all the Pearson correlation coefficient for epigenetic QTLs with cold tolerance (CT-QTLs^epi^) and quality and yield (QY-QTLs^epi^); (**b**) Distribution of epigenetic QTLs significantly associated with cold tolerance (CT-QTLs^epi^) and quality and yield (QY-QTLs^epi^). The y-axis indicates the correlation coefficient (p-value < 0.01, *t* test) and x-axis indicates the length of the genome contigs (Mb).

We found 318 methylated QTL genes that were significantly associated with cold tolerance (CT-QTLGs^epi^, including 11 repeatable CT-QTLGs^epi^) and 524 that were significantly associated with quality and yield (QY-QTLGs^epi^, including 105 repeatable QY-QTLGs^epi^), based on the correlation analysis (x^2^ test, p < 0.01, Fig. 3 and Table S5). Among these CT-QTLGs^epi^, we observed *glycoside hydrolase family 2 β-mannosidase (GH family 2β-mannosidase*, Mes.gk016414) was significantly associated with CTIG, CTIF-4, LFIF-2 (Fig. S7a). Significantly correlated polymorphic methylation sites (SCPMs) in the promoter and coding region of *GH family 2β-mannosidase* were found, including these SCPMs in the CpG island of 3′ coding region. *Photosystem II protein D1* (Mes.gk020417) was observed significantly associated with LFIF-2 (Fig. S7b). Near the 5′ initiation codon area, several SCPMs were detected. Interesting, we also found SCPMs in photosystem II protein D1 CT-QTL^epi^ transcription factor binding sites in DNA sequence (reCT-QTL^epi^TFBSs). This gene has previously been shown to a general adaptive response to environmental extremes^20^. Photosystem II D1 protein degradation speed will be greater than the synthesis under various stress conditions, resulting the damage of photosystem II reaction center. Under cold stress, decreased membrane fluidity affected the diffusion rate of the damaged Photosystem II D1 protein, hindering the insert of newly synthesized Photosystem II D1 protein. Plant photosynthetic activity decreased obviously, as photosystem II D1 protein damage-repair turnover was interferenced by low temperature stress^21^. The SC124 *Photosystem II protein D1* genes expressions were higher than KU50 in the leaves and roots, which in accordance with their cold tolerance. Two *peroxidase superfamily protein (POD*, Mes.gk031011 and Mes.gk045763) were significantly associated with RIF-2 and RIG, respectively (Fig S7c and d). We found SCPMs in the coding region in both genes. Previous study showed these *POD* gene family members in response to environmental stimulus, including low-temperature stimulus^22^,^23^. In accordance with previous studies showed that *mitogen-activated protein kinase kinase 4 (MKK4*) were activated and rising its mRNA levels by cold stress in a mitogen-activated protein kinase pathway, suggested it could be a plant stress signaling^24^, we found *MKK4* (Mes.gk046937) was significantly associated with rSRY-2 (Fig. S7e). SCPMs were detected in *MKK4* coding region.

Among these QY-QTLGs^epi^, *phosphoserine aminotransferase (PSAT*, Mes.gk000126) was significantly associated with SRY-4, DW-2 and FSC-1 (Fig. S7f). SCPMs were detected in *PSAT* coding region. In previous studies observed serine took part in senescence, protein degradation, and Inhibit the growth of plants^25^,^26^. In accordance with previous study we found the *PSAT* gene expression level in the roots and leaves of KU50 were lower than that in SC124. *Snrnp auxiliary factor, small subunit* (Mes.gk030683) was significantly associated with DW-2, DW-3, FSC-1 (Fig. S7g), and *Zinc finger (RING-H2-type finger*, Mes.gk024887) was significantly associated with DW-2, FSC-1 (Fig. S7h). Besides, we found *translocon at the outer envelope membrane of chloroplasts 33 (TOC33*, Mes.gk016669), *chaperonin 20 (CPN20*, Mes.gk033730), *NADH-dependent glutamate synthase 1 (GLT1*, Mes.gk036403) were significantly associated with DW-2, DW-3 (Fig. S7i,j and k). SCPMs were detected in *PSAT, Snrnp auxiliary factor, RING-H2-type finger, CPN20, GLT1* gene coding regions, while were detected in *TOC33* intron region. Besides, we found SCPMs in *Ribulose-1,5 bisphosphate carboxylase/oxygenase large subunit N-methyltransferase* (Mes.gk014095) and *RNA methyltransferase* (Mes.gk015292) QY-QTL^epi^TFBSs, and their adjacent genes expressions were both higher in SC124 than KU50. These data may reflect a metastable phenomenon in the heritable hemi-methylation and full-methylation patterns in cassava.

### High dense AFSM linkage group maps based on KS population

**Figure 4:**
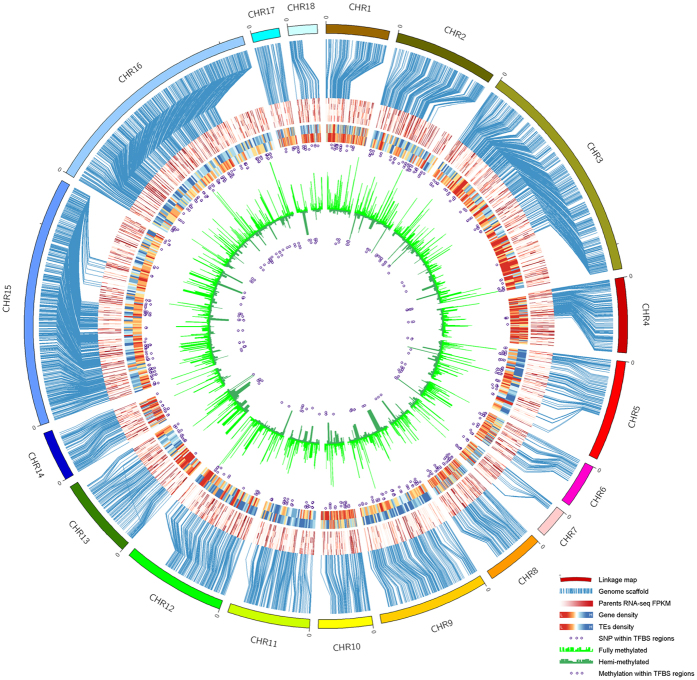
A high density KS genetic map. Outside cylindrical, KS genetic linkage groups; Blue lines, synteny between the scaffold and genetic linkage groups; Red lines, expressed genes from outside to inside, are root of SC124, leaf of SC124, root of KU50, and leaf of KU50; Blue and red lines, gene and TEs density (the outside for gene, the inside for TEs); Purple circles in the middle, SNPs within predict transcription factor binding sites in DNA sequence (TFBSs); Light green, Fully-methylated sites; Dark green, hemi-methylated sites; Purple circles in the center, Methylated sites within TFBSs.

In addition to methylation, 573,557 single nucleotide variants (SNVs; SNP and indel markers) were identified using this approach. Among the 573,557 SNVs, 10,627 were distributed in the CDS region, 22,439 in the gene region, and 8,709 in the promoter region. A high density KS genetic map was produced for the KS cassava mapping population using JoinMap version 4.1 to estimate the linkage, map order, and distance (Fig. 4 and Table S8). This study is attempt to develop the biggest cassava genetic linkage map using only AFSM markers. Four thousand six hundred and forty-eight out of 69,141 filtered AFSM markers (consisting of 4,437 SNVs and 211 methylation polymorphic markers) were assigned to 18 linkage groups (LGs). A total of 2,605 of the markers were located within gene regions. The identification of 18 LGs is consistent with cytological studies reporting the diploid number of chromosomes in cassava^27^. The lengths of the LGs varied from 38.39 (LG 1) to 235.26 cM (LG 15), and the KS genetic map spanned a total of 2190.34 cM. A total of 4,648 AFSM linkage group markers were associated with 2,734 KU50 scaffolds. Then, 38,380 AFSM makers were mapped to these scaffolds. Using the single copies, the KU50, AM560 and W14 genomes were associated with each other. We combined 461 AM560 scaffolds and 933 W14 scaffolds into 18 linkage groups using 1,634 and 1,408 AFSM linkage group markers, respectively (Fig. S8 and Table S8).

### Fine mapping of QTLs and relative genes for cold tolerance, yield and other economic traits

**Figure 5:**
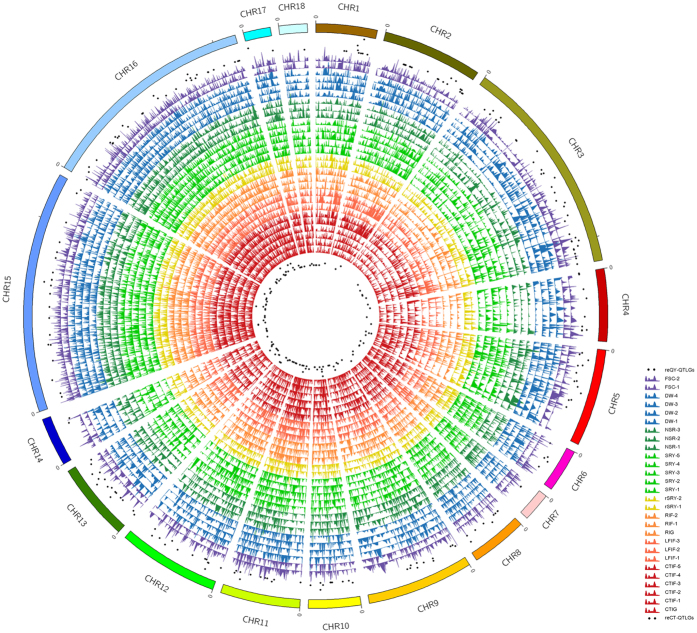
QTL mapping results for cold tolerance-related traits (CTRTs) and yield and quality-related traits (YQRTs) in KS population. QTL mapping profiles for four independent CTRTs measurements [Red: Cold-tolerance index in greenhouse (CTIG) and Cold-tolerance index in field (CTIF) 1–5; Dark orange: Leaf fall index (LFIF) 1–3; Light orange: Recovery index in greenhouse (RIG) and Recovery index in field (RIF) 1–2; Yellow: Relative storage root yield (rSRY) 1–2], as well as four independent YQRTs measurements [Light green: Storage root yield (SRY) 1–5; Dark green: Number of storage root (NSR) 1–3; Blue: Storage root dry weigh (DW) 1–4; Purple: Fresh root starch content (FSC) 1–2].

Our linkage mapping obtained 574 repeatable cold-tolerance QTLs and 499 repeatable quality and yield QTLs (Fig. 5 and Table S8). To explore the biological functions of these QTLs, we entered them into the MapMan software for pathway analysis. We detected 260 genes with repeatable cold-tolerance QTLs (reCT-QTLGs) and 301 genes with repeatable quality and yield QTLs (reQY-QTLGs) in these pathways (Tables S9 and S10). Phytohormones play important roles in the adjustment to adapt to the environmental stresses^22^,^28^,^29^. Consistent with these findings, ABA biosynthesis reCT-QTLG (*nine-cis-epoxycarotenoid dioxygenase 3, NCED3*), jasmonate biosynthesis reCT-QTLG (*OPDA reductase 3, OPR3*) and ethylene biosynthesis reCT-QTLGs (1-aminocyclopropane-1-carboxylate synthase 7 and ethylene signal DNA binding/transcription factor) were detected in the hormone synthesis and metabolism pathway (Fig. S9a). *Calmodulin-dependent protein kinase (CDPK*), *CBL4, CaLB* and *IQ-domain 10 (IQD10*) reCT-QTLGs were found in the calcium/calmodulin-mediated signaling network (Fig. S9a), indicating that these QTLs might be related to the cold tolerance of cassava. These findings contrast with the previously reported result that calcium/calmodulin-mediated genes play important roles in cold stress response for plant^30^,^31^. In a general adaptive response to cold stress, plants always grow slow or stop growing. In this study, we found SNVs within *auxin response factor 2* (ARF 2) (Mes.gk011443) reCT-QTLTFBS, and its adjacent gene expression in SC124 is significantly lower than that in KU50. This may be one of strategies for SC124 to be more cold resistant than KU50, by slowing or stopping growth during chilling stress. WD-40 repeat family protein is associated with biomass accumulation. We found SNVs within *WD-40 repeat family protein* (Mes.gk025413) reQY-QTLTFBS, and its adjacent gene expression in SC124 is significantly higher than in KU50.

In addition, we identified 25 repeatable cold-tolerance QTL transcription factors (reCT-QTLTFs, Fig. S9b), out of 46 CT-QTL transcription factors (CT-QTLTFs). *WRKY* genes showed strong and rapid induction in cold stress response in previous studies^32^,^33^. Consistent with these findings, we identified WRKY reCT-QTLTFs (*WRKY31* and *WRKY9*). *Myb domain protein 55 (MYB55*), *MYB70, MYB40, helix–loop–helix (bHLH*), *auxin response factor 2 (ARF 2*) and *zinc finger protein 6 (ZFP6*) reCT-QTLTFs were also detected in our study (Fig. S9b). Moreover, we identified *sucrose phosphate synthase 2F (SPS2F*), *sucrose synthase 6 (Susy6*), *neutral invertases, fructokinase*, and *beta-amylase (BAM*) reQY-QTLTFs in the starch and sucrose metabolism pathway (Fig. S9c,d and e). These QTLs might play important roles in improving the cold tolerance, high quality and yield of cassava.

## Conclusion

In summary, we combined novel genome-wide CCGG hemi-methylation and full-methylation analysis and the SNP discovery sequencing approach AFSM, the transcriptome deep sequencing method RNA-seq and years of independent field and greenhouse trials in the cassava KS population and the parents to enable the high-resolution genome-wide characterization of a map with CCGG DNA methylation sites, Gene and TEs density, SNPs, repeatable QTLs, QTLs^epi^ and related gene expression profiles. In addition to a large number of SNVs, we also identified thousands of hemi-methylated and fully methylated sites using the same genotypes. We described the distribution of these methylated sites in the cassava genome and grouped them into twelve classes based on the hemi-methylated or fully methylated site heritability in the KS population. Furthermore, we provide the description of the DMR-DEGs in cassava.

The complete set of whole-genome CCGG DNA methylation and gene expression data can be downloaded from the NCBI Sequence Read Archive (SRA) (SRX1674579 and SRX53531 for AFSM data, SRR2361999, SRR2404206, SRR2495947 and SRR2496326 for RNA-seq data). The related software including AFSM Perl scripts can be downloaded at (http://afsm.strikingly.com/). We build a new SNP and methylation genome browse website for cassava at (http://192.64.83.141/JBrowse-1.11.5/?data=test). We showed 77 much candidate QTLs^epi^ for cold stress and 103 much candidate QTLs^epi^ for storage root quality and yield (Tables S6 and S7). Besides, 260 much candidate QTL genes for cold stress and 301 much candidate QTL genes for storage root quality and yield were also presented at Tables S9 and S10. These new tools and genome-wide resources should serve as the molecular basis for future cassava marker-assisted breeding programs and highlight the discovered cold-tolerance and high-quality QTLs^epi^ and QTLs that may reduce timelines. Finally, the whole genome approaches developed here should be useful for future studies with many other organisms with large complex genomes and complex traits.

## Methods

### KS mapping population development

In this study, the KS mapping population of 186 progenies was generated by crossing two non-inbred lines with differentially cold-resistant. The female parent, KU50, possess many elite economic traits with high rate of starch and high-yielding, and is sensitive to chilling. The male parent, SC124 yielded high and is tolerant of chilling. Seeds were disinfected using sodium hypochloride and stratified in sterile water. Then the seeds were germinated in sterilized garden soil and transplanted two month after sowing. At maturity, the stem cutting were planted at Haikou, Hainan Provience (HK09), Wenchang, Hainan Provience (WC10, WC11, WC12, WC13), Guiling, Guangxi Provience (GL11, GL12), Hezhou, Guangxi Provience (HZ13) from 2009–2013.

### Cold-tolerance index: cold-tolerance index in greenhouse (CTIG) and cold-tolerance index in field (CTIF)

For each seedling line, eight seedlings were grown on 15Χ 15 cm pots containing sterilized garden soil. After two months, the strong and uniform seedlings were selected for chilling injury treatment in a phytotron at photon flux density of 800 μmol m^−2^ s^−1^ PAR, 60–80% relative humidity. The 20 °C/18 °C day-night (12 h/12 h) treatment was carried out for 24 h. After 15 °C/12 °C day-night (12 h/12 h) chilling treatment was performed for 24 h, then 6 °C/4 °C day-night cycle (12 h/12 h) chilling treatment was carried out by exposure to cool air for 5 d. The temperature was returned to 28 °C after the chilling treatment and the plants was allowed to recover for 24 h. The CTIG was calculated as follows:


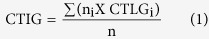


In this equation, CTLG_i_ was the cold-tolerance level in greenhouse (0–4 levels), n_i_ was the number of plants with the same cold-tolerance level.

The entire population was planted at Wenchang in Hainan province (19.20° North latitude, 108.21° East longitude) and Guilin (24.18° North latitude, 109.45° East longitude) or Hezhou (23.39° North latitude, 111.05° East longitude) in Guangxi province using a randomized complete block design. Each clone was planted in three replicated plots of ten individuals per plot in each line, with plant spacing of 1m × 0.8 m for four 12-month long cropping seasons (2010, 2011, 2012 and 2013). Generation-to-generation propagation through cloning was based on use of 15-cm long stem cutting in F1. The CTIF was measured after the temperature fell to 15 °C/10 °C day-night in winter for 7 d. We consider five CTIF measurements. Two CTIF measurements (CTIF1 and CTIF5) were come from Wenchang field experiments in 2010 and 2013, one (CTIF4) from Hezhou field experiment in 2013, and two (CTIF2 and CTIF3) were from Guilin field experiments in 2011 and 2012. The CTIF was calculated as follows:


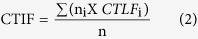


In this equation, CTLF_i_ was the cold-tolerance level in field (0–4 levels), n_i_ was the number of plants with the same cold-tolerance level.

### Leaf fall index in field (LFIF)

The total numbers of leaves (NL) and leaves fall (NLF) were recorded through the leaf scars in the early winter when temperature was above 10 °C. The first (LFIF1), second (LFIF2) and third (LFIF3) measurements of LFIF were performed. Measurement LFIF1 came from Guilin in 2012, LFIF2 measurement came from Hezhou in 2013, and LFIF3 measurement from a Wenchang field experiment in 2013. The LFIF was calculated from the following equation:


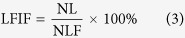


### Recovery index in greenhouse (RIG) and Recovery index in field (RIF)

The temperature was returned to 28 °C after the chilling treatment and the plants was allowed to recover for 10 d. The RIG was calculated from the following equation:


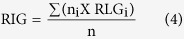


In this equation, *RLG*_*i*_ was the recovery level in greenhouse (1–4 levels), n_i_ was the number of plants with the same recovery level.

The temperature was returned to 20 °C/15 °C day-night in the next year early spring and the plants was allowed to recover for 14 d. The first (RIF1) and second (RIF2) measurements of RIF were performed in 2010 and 2013. The RIF was calculated from the following equation:


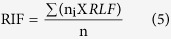


In this equation, RLF_i_ was the recovery level in field (0–4 levels), n_i_ was the number of plants with the same recovery level.

### Relative storage root yield (rSRY)

Storage root samples were harvested between February and March (11–12 months old plants). Two rSRY measurements (rSRY1–2) were performed. Measurement rSRY1 was root tuber yield from Guilin field relative to the root tuber yield from Wenchang field in 2012, and measurement rSRY2 was root tuber yield from Hezhou field relative to the root tuber yield from Wenchang field in 2013.

### Storage root yield (SRY)

We performed five SRY measurements (SRY1–5). Measurements SRY1, SRY2 and SRY5 come from Wenchang field experiments in 2010, 2012 and 2013, SRY3 measurement from a Guilin field experiment in 2012 and, SRY4 measurement from a Hezhou field experiment in 2013.

### Number of storage root: (NSR)

Three measurements of NSR1–3 were performed. Measurements NSR1 and NSR3 come from Wenchang field experiments in 2010 and 2013, and NSR2 measurement from Hezhou field experiment in 2013.

### Storage root dry weigh (DW)

DW was determined using the method of Benesi *et al*.^34^. Six undamaged roots were randomly selected. The medial sections of selected fresh roots were shredded into thin slices, mixed thoroughly and duplicate of 200 g (w1) were oven dried at 65 °C for 72 h. Dry matter content was weighed immediately (w2). Four measurements (DW1–4) were performed for four years (2010, 2011, 2012 and 2013). The percentage of storage root dry weigh (DW%) was calculated via the following equation:


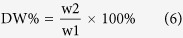


This was done within 12 h after harvest to avoid post harvest changes through physiological deterioration or moisture loss of the root.

### Fresh root starch content (FSC)

FSC measurements (FSC1–2) were done in Wenchang over 2 years (2011 and 2013). Starch content was analyzed using a Total Starch Assay kit (Megazyme International, Wicklow, Ireland), and spectrophotometric readings were conducted using a Spectronic 1201 spectrophotometer (Milton Roy Company, Ivyland, PA, USA) using glucose as sugar control and maize starch as the starch control. The starch content of the storage roots was first calculated as a percentage per dry weight basis, and later converted to a percentage per fresh weight basis for analysis.

### Principal coordinate analysis

A principal coordinate analysis (PCoA) was constructed based on the binary character matrix using the princomp in R^35^. The PCoA plot revealed the structeristics of a binary data matrix of complex traits in the entire population. All complex traits were approximately scatted in two areas (Fig. S6): The cold-tolerance related traits (CTRTs, right blue circle) and the yield and quality related traits (YQRTs, left green circle).

### AFSM library construction and sequencing

DNA from the fully expanded leaves of 5-month-old crops was extracted using Plant DNeasy Maxi Kit (QIAGEN, Valencia, CA), with two biological replicates were pooled for each individual. AFSM libraries was constructed using AFSM method^16^, which can concurrently identify whole genome SNPs, indels, fully-methylation and hemi-methylation sites for 186 samples from KS population. AFSM libraries were sequenced using Illumina HiSeq2500 with Pair-end 150 bp lengths.

### RNA-seq library construction and sequencing

Total RNA was extracted from the fully expanded leaves and storage roots of the parents of KS population at the same developmental stage (120 days), using RNA plant reagent kit (Tiangen Company). The RNA-seq libraries were performed according to Illumina manufacturer’s instructions (Illumina). Then, using Illumina HiSeq2500, 51 bp sequencings were performed.

### Alignments of Illumina AFSM reads and data analysis

The raw Illumina AFSM sequence reads were processed using custom Perl scripts^16^ and then aligned to the MK_v1 cassava genome using Bowtie2^17^, allowing one mismatch. SNPs were identified using the SAMtools and VCFtools_v0.1.9 (http://vcftools.sourceforge.net/). Besides, custom algorithms were used for methylation analyses as decribed by previously^16^.

### Methylation analyses

Analyses of the AFSM methylation results were based on comparisons of the EcoRI-HpaII- and EcoRI-MspI-assembled sequences with methylated cytosines at the 5′-CCGG sites using custom Perl scripts (http://afsmseq.sourceforge.net/) for individual plants, described as Xia *et al*.^16^. In this study, a CCGG methylated site was definited as that present at more than 4 reads (at least 2 HpaII-reads and 2 MspI-reads). For each individual assembled sequence, it was first determined whether those with CCGG sites were: (1) present only in the HpaII cleavage sites of the EcoRI-HpaII products and the body sequences of the EcoRI-MspI products but not in the MspI-cleaved sites; (2) present only in the MspI cleavage sites of the EcoRI-MspI products and the body sequences of the EcoRI-HpaII products but not in the HpaII cleavage sites; (3) present in the body sequences of both the EcoRI-HpaII and EcoRI-MspI products but not in the HpaII or MspI cleavage sites. Condition (1) denotes a hemi-mCCGG methylated state, and conditions (2) and (3) correspond to fully CmCGG and fully mCCGG methylated states. Only methylated sites that were similarly methylated at least in two samples were remained in this study.

Methylated density was computed as described in Weiss^36^ and each region was split into 80 equal windows, with the average alignment depth calculated for each window. Methylated genes were determined only when a methylated site similarly methylated at least in two samples in cassava gene body or promoter (2 kb upstream) regions.

We refer to the level of methylation of genomic regions in this study. To compute this level within a bin, we summed the number of methylated CCGG (the number of methylated CCGG sites multiplied by the number of reads from methylated fragments within a bin), and divided the summed number of sequenced bases covering all CCGG (the number of CCGG sites multiplied by the number of reads from all fragments within a bin).

DMRs were identified using our pipeline. Each methylation was scanned genome-wide requiring at least 5 methylated CCGG differences within a given window. DMRs were identified by comparison of the leaf and root in SC124 and KU50 methylations(SC124 leaf vs. KU50 leaf, SC124 root vs. KU50 root, leaf vs. root in KU50 and leaf vs. root in SC124). A DMR was identified if the P-value from Chi-squared test was ≤ 0.05.

### Alignments of Illumina RNA-seq reads and data analysis

Adapters were removed from raw Illumina RNA-seq sequence reads using FASTX-toolkit pipeline, version 0.0.13 (http://hannonlab.cshl.edu/fastx_toolkit/). Sequence quality was examined using FastQC (http://www.bioinformatics.babraham.ac.uk/projects/fastqc/). Reads were mapped to the cassava genome (MK_v1)^14^ using Tophat v. 2.0.10^37^. Gene level expression and differential expression analysis were performed using the program Cufflinks version 0.8.0^38^ with default settings. To test DE with unambiguous mapping data DEGseq^39^ was used.

### Functional annotation

For mapping KU50 genes onto the Kyoto encyclopedia of genes and genomes (KEGG)^40^ pathway annotation, we used a strategy similar to the KAAS method (KEGG Automatic Annotation Server)^41^, which is based on reciprocally best blast similarity hits against all KEGG orthology (KO) groups of functionally related genes assigned in the KEGG GENES database. The KU50 genome is still only partially annotated, we also allowed one-directional best blast hits with significant *E*-values (<1*e* − 5) to annotate additional sequences. To assign predicted proteins to MapMan categories, all proteins were used in a blast search (NCBI Blast version 2.2.16) against plant proteins, which had previously been classified using the MapMan classification system^42^. Here, all blast-derived hits with bit scores of ≤50 were excluded from further analysis. Furthermore, all sequences were scanned for known motifs and/or families using InterProScan^43^. Gene Ontology (GO) enrichment analysis was performed using BiNGO (Biological Networks Gene Ontology)^19^, a Cytoscape 2.8.3 plugin that allows the over representation of GO terms to be analyzed in a given set of genes within a statistical framework. Potential transcription factor binding sites were analyzed using TESS (Transcription Element Search http://www.cbil.upee.edu/tess) and the Blastn (NCBI) algorithms.

### Cassava linkage map construction

To identify the SNPs, indels and methylation polymorphic sites, all pairs of tags were evaluated for the presence of at least two reads. JoinMap 4.1^44^ was used to construct the cassava linkage map for the KS population. Bi-allelic SNPs, indels and methylation polymorphic sites were identified by querying the filtered tags for pairs of sequences with the following characteristics: 1) identical in at least two reads; 2) present in >50% of the individuals; 3) passed a Fisher’s exact test for independence; 4) fit to the expected Mendelian segregation ratio as demonstrated by a chi-squared test at a P < 0.01; 5) possessed a threshold of ≤5.0 with a LOD score of >1.0; 6) possessed a recombination frequency of <0.4; and 7) had AFSM markers specific to the female or male parent that fit to a 1:1 segregation ratio in addition to shared AFSM markers that fit to a 3:1 segregation ratio in the KS population. If a SNP, indel or methylation polymorphic site call was heterozygous, presumably due to sequencing errors, then the call was classified as missing data. According to JoinMap 4.1, the KS population could be considered as a CP population according to the genetic background if the two parents were heterozygous. The parent-specific AFSM markers, which segregated at a 1:1 ratio in the population, were recorded as lm × ll (marker in the female parent) and nn × np (marker in the male parent). The AFSM markers that were present in both parents and segregated at a 3:1 ratio in the population were recorded as hk × hk (marker present in both parents).

### Cassava comparative map construction

Single copy homologous genes from KU50, AM560 and W14 were used as connection points, for performing comparative genomes analysis. The CirCOS map was constructed using Circos (http://circos.ca/)^45^.

### Analysis of parentally-derived QTL^epi^

Among the 47,395 filtered methylation sites (only samples with overlaps between polymorphic methylation sites and phenotypes ≥20 are remained), we scored fully methylated sites as “a”, hemi-methylated sites as “2/3a”, and non-methylated sites as “0”. The significant correlation between polymorphic methylated sites and phenotypes were calculated using Pearson correlation coefficients (p-value < 0.01, t test).

The t test was performed with N-1 degrees of freedom.


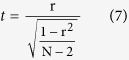


PDL::GSL::CDF parameter: pvalue_2 tail = 2 × (1 − gsl_cdf_tdist_P(abs(t), N − 2)). N is the number of samples with overlap between significantly polymorphic methylated sites and phenotypes.


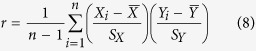


### Analysis of parentally-derived QTL

We used the framework maps to search for QTLs on parental and consensus maps. First, the nonparametric Kruskal–Wallis (KW) rank-sum test was carried out at each marker on raw phenotypic data with MapQTL 5.0^46^ for muscat score and each raw monoterpene content (with a first type error rate of a = 0.001 for each individual test). We used MapQTL for KW tests at each marker, with a = 0.001 for individual tests, for parental and consensus maps. Then KW tests, Simple Interval Mapping (SIM) and Composite Interval Mapping (CIM) were performed on ln-transformed monoterpene contents. Composite interval mapping (CIM) was performed to identify QTL controlling LL using the MQM method with the program MapQTL 5.0 and the appropriate cofactor selection. The permutation test^47^ was performed with 1000 runs to determine the P = 0.05 genomewide significance level for declaring QTL for LL significant, according to the complementarities of these softwares.

## Additional Information

**Accession codes:** Sequence Read Archive (SRA) database SRX1674579, SRX53531, SRR2361999, SRR2404206, SRR2495947 and SRR2496326. SNP and methylation genome browse website for cassava Browse website: http://192.64.83.141/JBrowse-1.11.5/?data=test.

**How to cite this article**: Zou, M. *et al*. Epigenetic map and genetic map basis of complex traits in cassava population. *Sci. Rep.* **7**, 41232; doi: 10.1038/srep41232 (2017).

**Publisher's note:** Springer Nature remains neutral with regard to jurisdictional claims in published maps and institutional affiliations.

## Supplementary Material

Supplementary Materials
