# Supplementary material for: Epigenetic map and genetic map basis of complex traits in cassava population
Source: Sci Rep. 2017 Jan 25;7:41232. doi: 10.1038/srep41232 (PMC5264614; doi:10.1038/srep41232)
Supplement: Supplementary Materials [file srep41232-s1.pdf]

Supplementary Materials for

Epigenetic map and genetic map basis of complex traits in cassava population

Meiling Zou[1,2], Cheng Lu[2], Shengkui Zhang[2], Qing Chen[2], Xianglai Sun[2], Pingan Ma[2], Meizhen Hu[2], Ming Peng[2], Zilong Ma[2], Xin Chen[2], Xincheng Zhou[2], Haiyan Wang[2], Subin Feng[2], Kaixin Fang[2], Hairong Xie[2], Zaiyun Li[1], Kede Liu[1], Qiongyao Qin[2], Jinli Pei[2], Shujuan Wang[2], Kun Pan[2], Wenbin Hu[2], Binxiao Feng[2], Dayong Fan[3], Bin Zhou[3], Chunling Wu[3], Ming Su[3], Zhiqiang Xia[1,2*], Kaimian Li[2*], Wenquan Wang[2*]

[1]Huazhong Agricultural University, Wuhan, China.
[2]The Institute of Tropical Biosciences and Biotechnology, Chinese Academy of Tropical Agriculture Sciences, Haikou, China.
[3]Guangxi Academy of Agricultural Sciences, Guilin, China.

*To whom correspondence should be addressed. E-mail: wangwenquan@itbb.org.cn (W.W.); zqiangx@gmail.com (Z.X.); likaimian@itbb.org.cn (K.L.)

Supplementary File	Title
Supplementary Figure 1	Annotation of monomorphic fully methylation in population, with BGI WEGO (P<0.001, χ^2 text), compared with cassava KU50 genes
Supplementary Figure 2	The percentage of cytosine methylation at the CCGG sites in the cassava KS population.
Supplementary Figure 3	The profile of CCGG methylation density in the TEs region.
Supplementary Figure 4	Differentially methylated regions (DMRs) relative to the cassava KU50 genes were functionally categorized, and significant differences were assessed using BiNGO (P<0.1, χ^2 text)
Supplementary Figure 5	Phenotypic distributions using box-whisker plots for cassava KS population with sample median.
Supplementary Figure 6	A principal coordinate analysis (PCoA) of the complex traits for cassava KS population. All complex traits were approximately scatted in two areas: the cold-tolerance related traits (CTRTs, blue circle) and the yield and quality related traits (YQRTs, green circle).
Supplementary Figure 7	DNA methylation profiles of methylated QTL genes (QTLGs^{epi}) in KS population (A, B, C, D, E, F, G, H, I, J, K,)
Supplementary Figure 8	A CirCOS (http://circos.ca/) figure showing synteny among three paralogous cassava genomic regions

Supplementary Figure 9	Proposed cassava cold-tolerance QTL genes (CT-QTLGs) and quality and yield QTL genes (QY-QTLGs) pathways (A and B for CT-QTLGs pathways; C, D and E for QY-QTLGs pathways)
Supplementary Table 1	A table of methylation patterns in SC124 and cassava KU50 parents and their F1 progenies
Supplementary Table 2	Summary of GO terms over-represented in functional type of D1 methylation of cassava
Supplementary Table 3	Annotation of monomorphic fully methylation in population, with BGI WEGO (*P*<0.001, χ^2 text), compared with cassava KU50 genes
Supplementary Table 4	Annotation of significantly differential methylation and differentially expressed genes in Cassava (DMR-DEGs) with BiNGO (*P*<0.1, χ^2 text) compared with cassava KU50 genes
Supplementary Table 5	QTL^{epi} results in KS population
Supplementary Table 6	Candidate key $QTLs^{epi}$ for cold stress
Supplementary Table 7	Candidate key $QTLs^{epi}$ for storage root quality and yield
Supplementary Table 8	Genetic analysis of cassava, including SNVs, QTLs and annotations
Supplementary Table 9	Genes with repeatable cold-tolerance QTLs (reCT-QTLGs)
Supplementary Table 10	Genes with repeatable quality and yield QTLs (reQY-QTLGs)

This PDF file includes:

Figs. S1 to S9
Tables S1, S2, S4, S6, S7, S9 and S10

Other Supplementary Material for this manuscript includes the following:

Tables S3, S5 and S8 as Excel files
Table S3, S5 and S8 can be download from http://192.64.83.141/ftp/

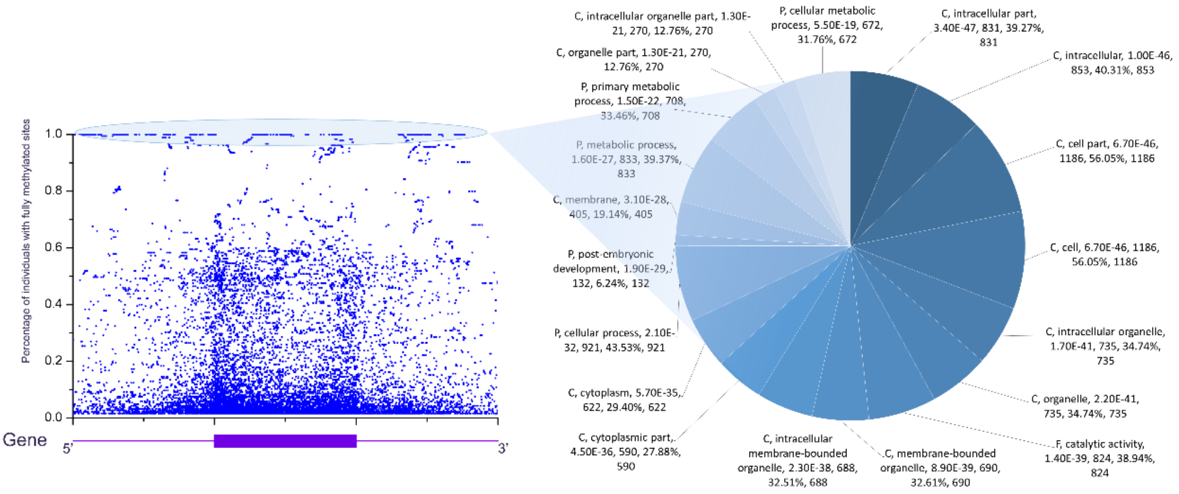

Fig. S1

Annotation of monomorphic fully methylation in population, with BGI WEGO (P<0.001, χ^2 text), compared with cassava KU50 genes[14]

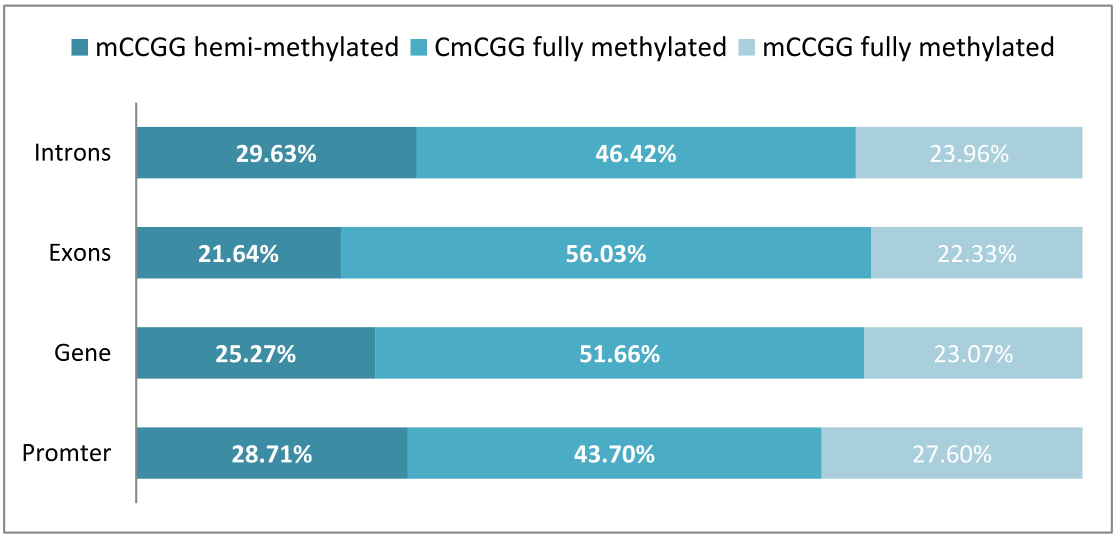

Fig. S2

The percentage of cytosine methylation at the CCGG sites in the cassava KS population.

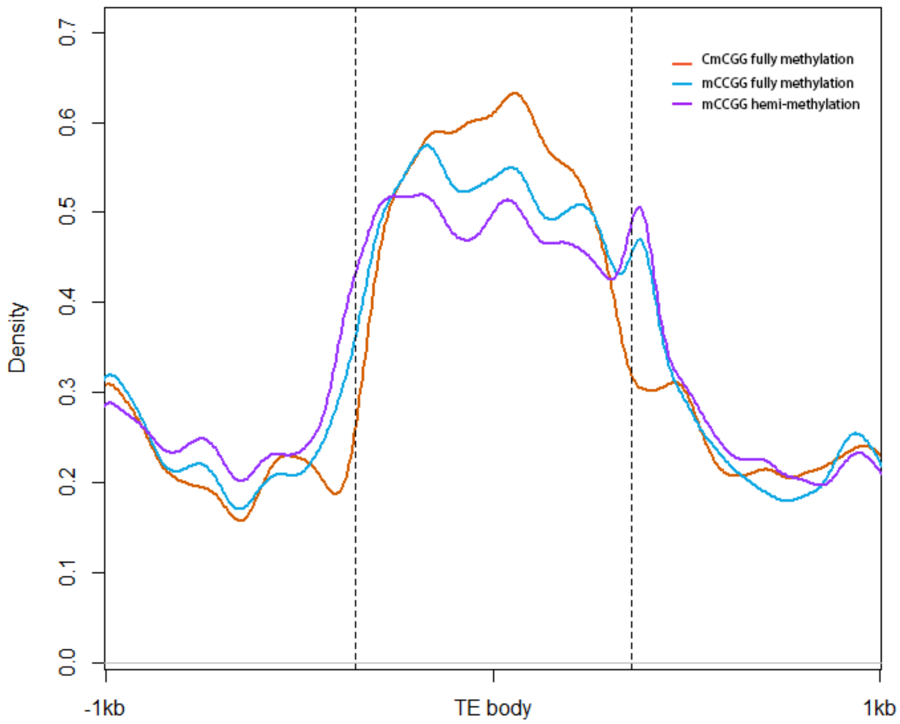

Fig. S3

The profile of CCGG methylation density in the TEs region.

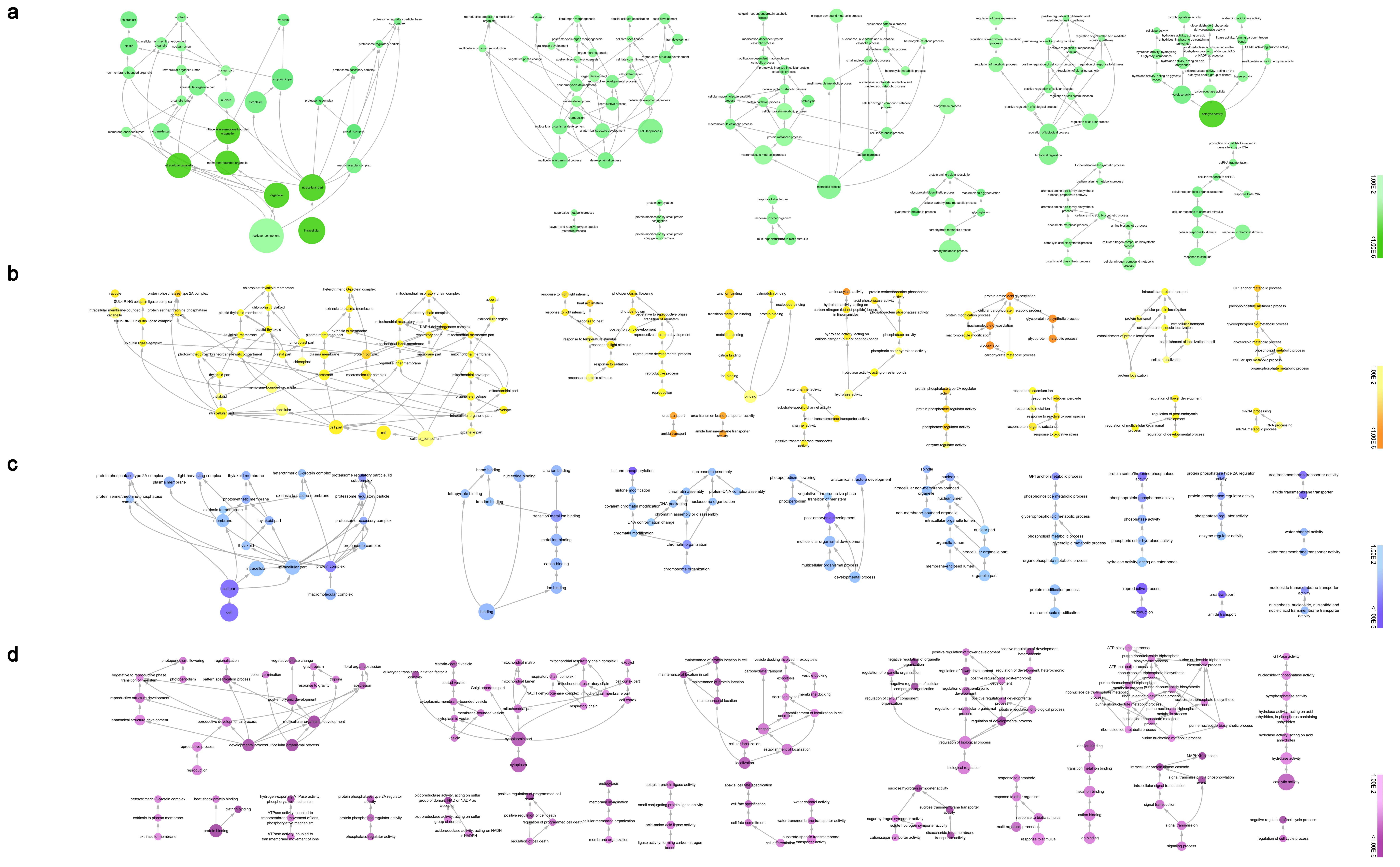
a
b
c
d
1.00E-2
<1.00E-6

Fig. S4

Differentially methylated regions (DMRs) relative to the cassava KU50 genes[14] were functionally categorized (a. DMRs of SC124 leaf vs. KU50 leaf, b. DMRs of SC124 root vs. KU50 root, c. DMRs of leaf vs. root in KU50 and d. DMRs of leaf vs. root in SC124), and significant differences were assessed using BiNGO[19] (P<0.1, χ^2 text).

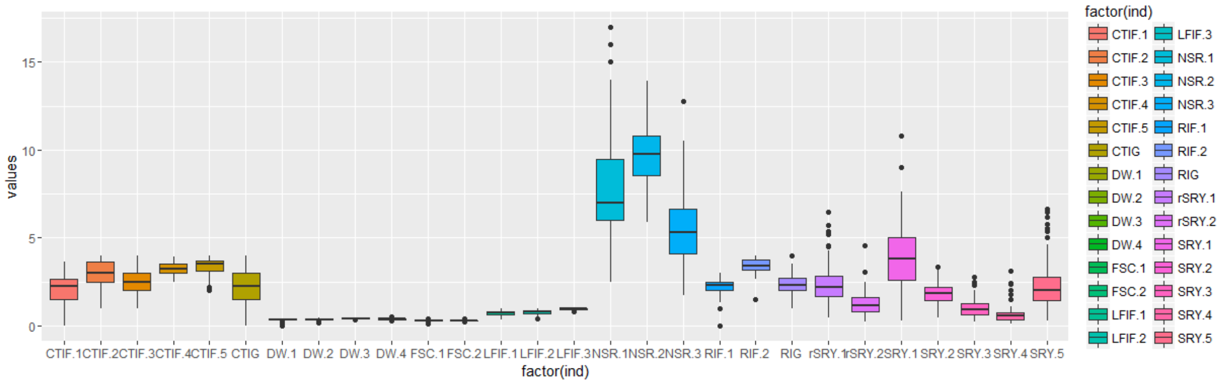

Fig. S5

Phenotypic distributions using box-whisker plots for cassava KS population with sample median. It was presented the cold tolerance-related traits (CTRTs) and yield and quality-related traits (YQRTs) distribution (as a violin plot) for stable complex traits that have an overlap with one or more individuals. We used vegetative propagation to replicate individuals from the mapping population, which enabled us to perform field and greenhouse experiments using the same genotypes in 2009-2013. The cold tolerance index ranged between 0 and 4.00. The recovery index ranged between 0.02 and 4.00. The leaf fall index ranged between 33.76% and 100%. The relative storage root yield ranged between 0.11 and 6.67. The storage root yield ranged between 0.22 and 10.80 kg. The number of storage roots ranged between 1.75 and 17.00. The storage root dry weigh ranged between 22.41 and 48.50%. The fresh root between starch content ranged between 14.79 and 39.1%. The y-axis indicates the correlation coefficient between methylated sites and phenotyping traits, while x-axis indicates the CTRTs and YQRTs

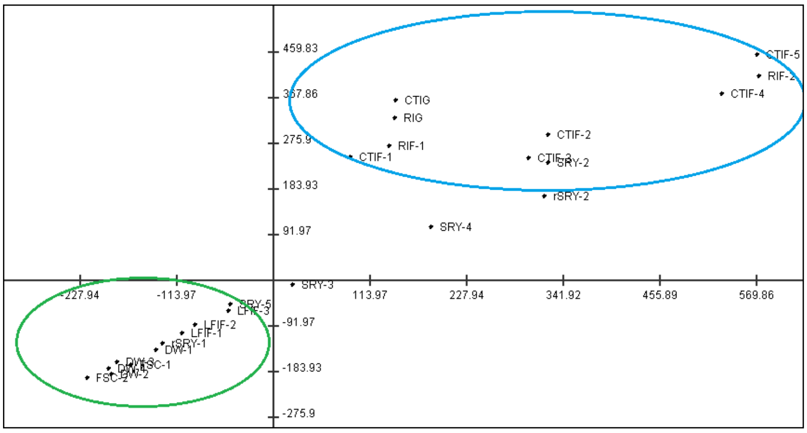

Fig. S6

A principal coordinate analysis (PCoA) of the complex traits for cassava KS population. All complex traits were approximately scatted in two areas: the cold-tolerance related traits (CTRTs, blue circle) and the yield and quality related traits (YQRTs, green circle).

a

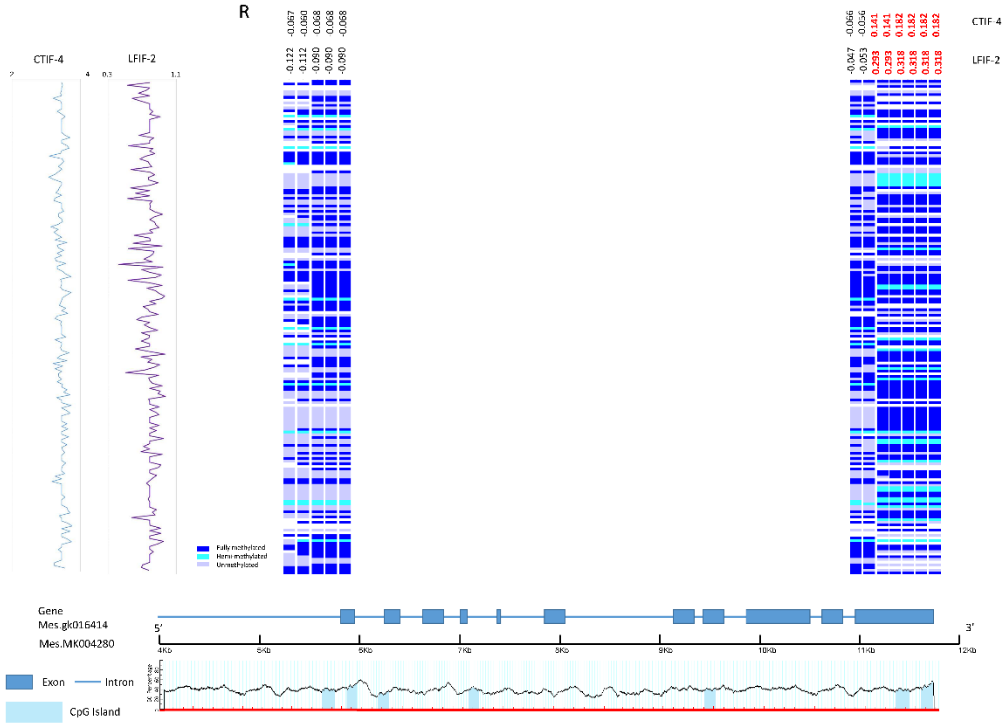
R
CTIF-4
LFIF-2
CTIF 4
LFIF-2
Fully methylated
Hemi-methylated
Unmethylated
Gene
Mes.gk016414
Mes.MK004280
5'
3'
Exon
Intron
CpG Island

b

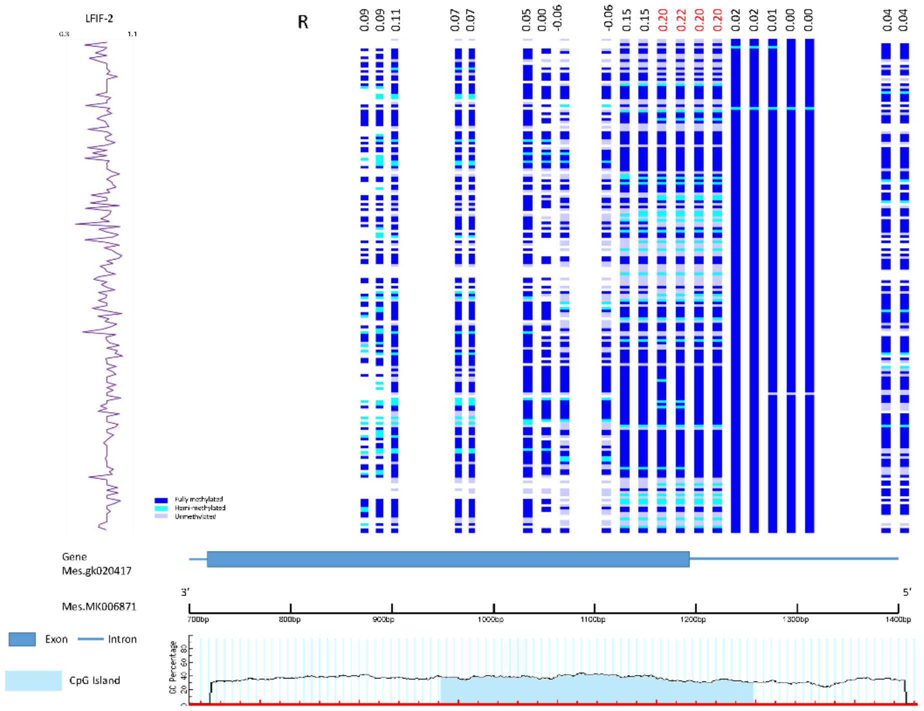
LFIF-2
R
Fully methylated
Hemi-methylated
Unmethylated
Gene
Mes.gk020417
Mes.MK006871
3'
5'
Exon
Intron
CpG Island

c

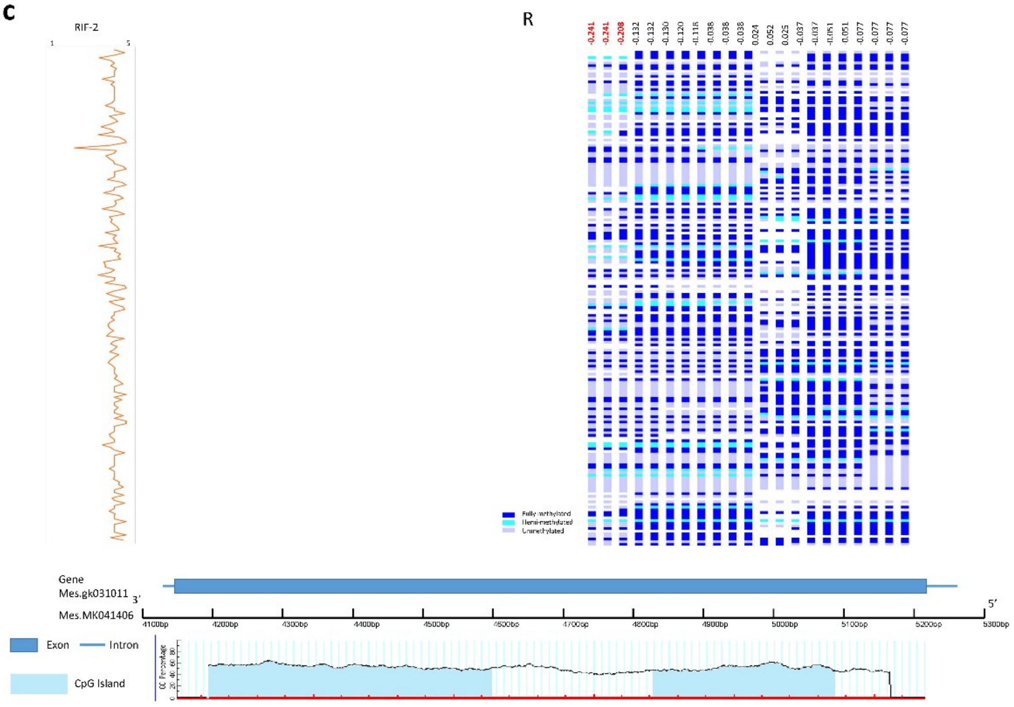

RIF-2
R
-0.241
-0.241
-0.208
-0.132
-0.132
-0.130
-0.120
-0.118
-0.038
-0.038
-0.038
0.024
0.052
0.025
-0.037
-0.037
-0.051
-0.051
-0.077
-0.077
-0.077
-0.077
Fully methylated
Hemi-methylated
Unmethylated
Gene
Mes.gk031011
3′
5′
Mes.MK041406
4100bp
4200bp
4300bp
4400bp
4500bp
4600bp
4700bp
4800bp
4900bp
5000bp
5100bp
5200bp
5300bp
Exon
Intron
CpG Island
GC Percentage

d

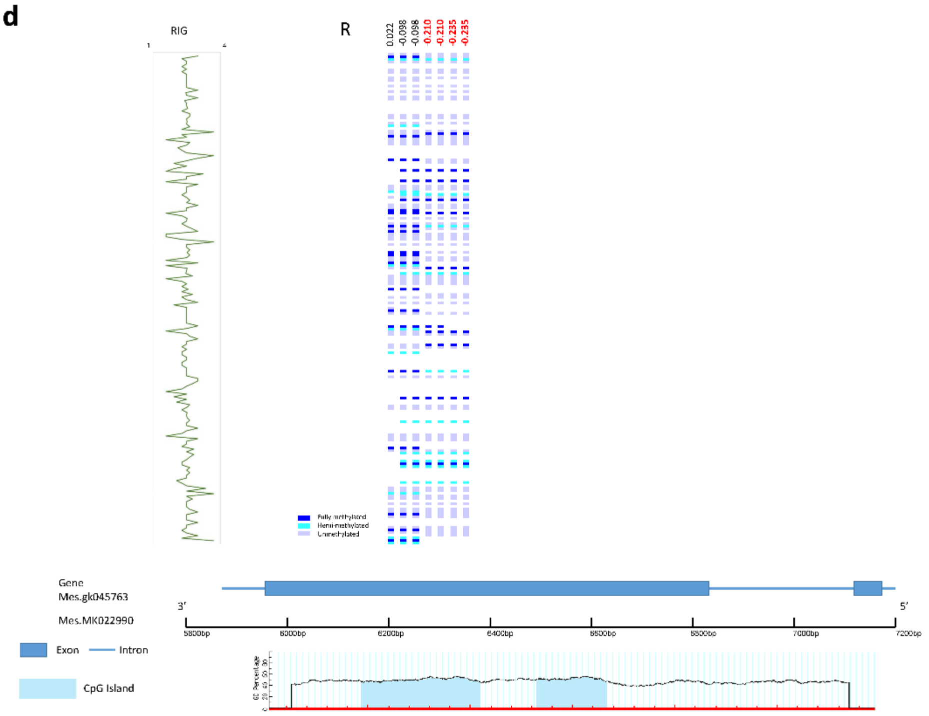

RIG
R
0.022
-0.098
-0.098
-0.210
-0.210
-0.235
-0.235
Fully methylated
Hemi-methylated
Unmethylated
Gene
Mes.gk045763
3′
5′
Mes.MK022990
5800bp
6000bp
6200bp
6400bp
6600bp
6800bp
7000bp
7200bp
Exon
Intron
CpG Island
GC Percentage

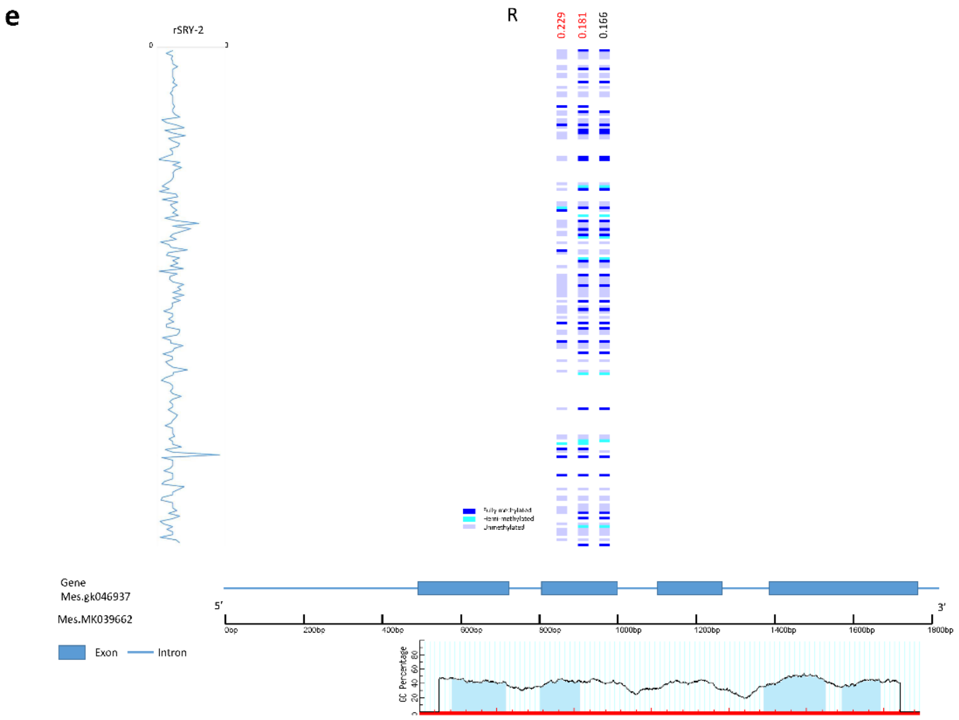
e
rSRY-2
R
0.229
0.181
0.166
Gene
Mes.gk046937
Mes.MK039662
5'
3'
Exon
Intron
GC Percentage

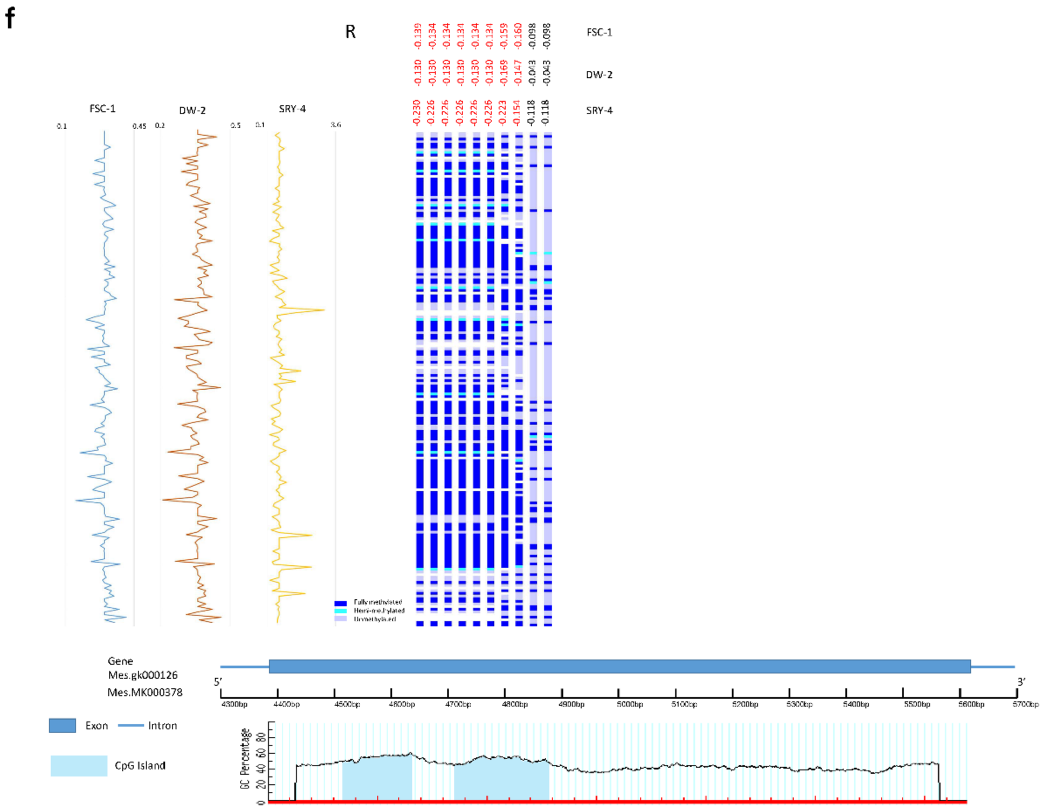
f
R
FSC-1
DW-2
SRY-4
Gene
Mes.gk000126
Mes.MK000378
5'
3'
Exon
Intron
CpG Island
GC Percentage

g

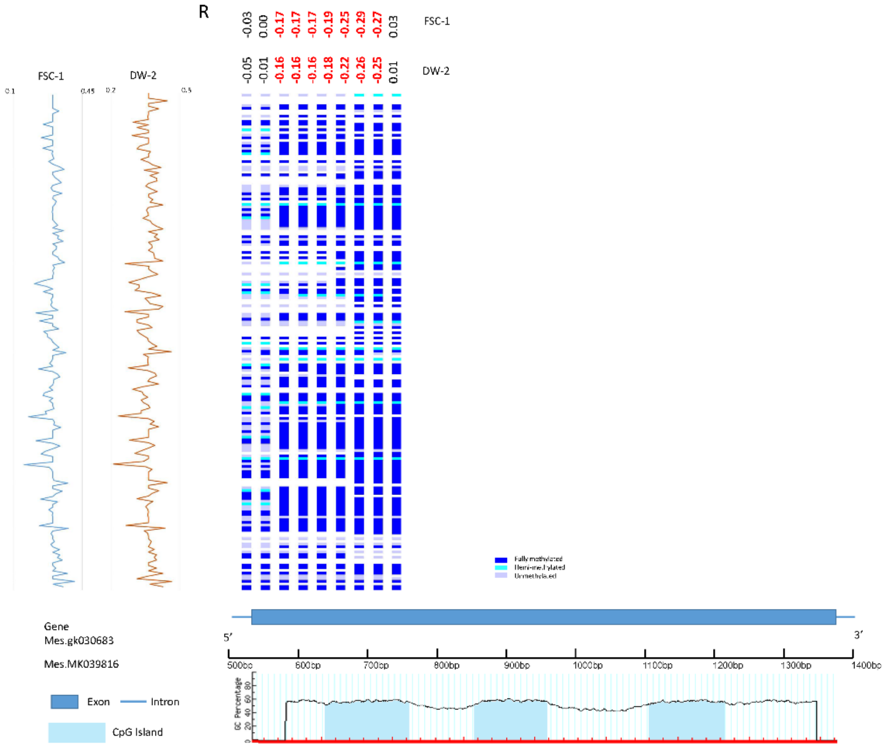
R
FSC-1
DW-2
-0.03 0.00 -0.17 -0.17 -0.17 -0.19 -0.25 -0.29 -0.27 0.03
-0.05 -0.01 -0.16 -0.16 -0.16 -0.18 -0.22 -0.26 -0.25 0.01
Fully methylated
Hemi-methylated
Unmethylated
Gene
Mes.gk030683
Mes.MK039816
Exon
Intron
CpG Island
5′
3′
500bp 600bp 700bp 800bp 900bp 1000bp 1100bp 1200bp 1300bp 1400bp
GC Percentage

h

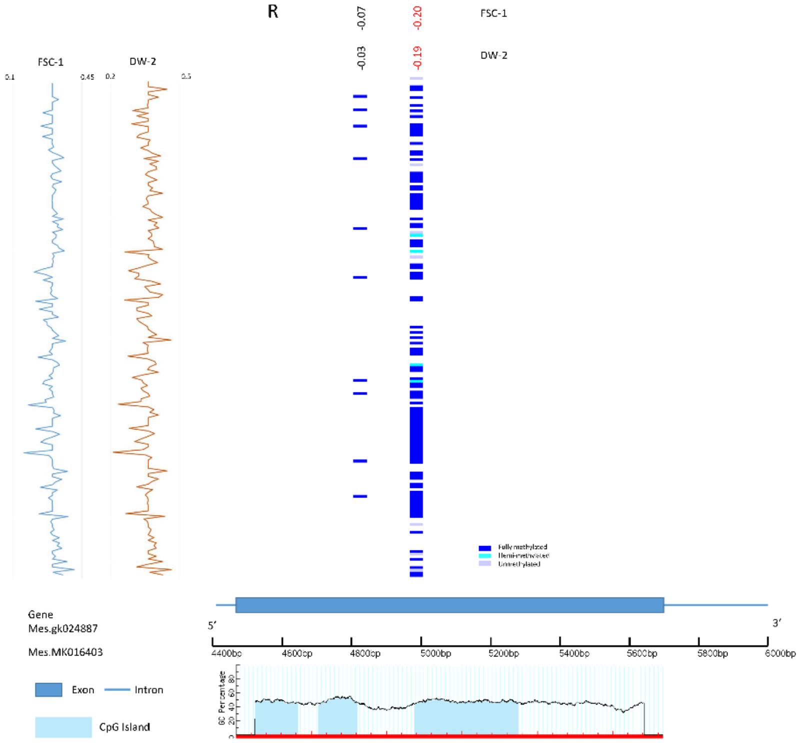
R
FSC-1
DW-2
-0.07 -0.20
-0.03 -0.19
Fully methylated
Hemi-methylated
Unmethylated
Gene
Mes.gk024887
Mes.MK016403
Exon
Intron
CpG Island
5′
3′
4400bp 4600bp 4800bp 5000bp 5200bp 5400bp 5600bp 5800bp 6000bp
GC Percentage

i

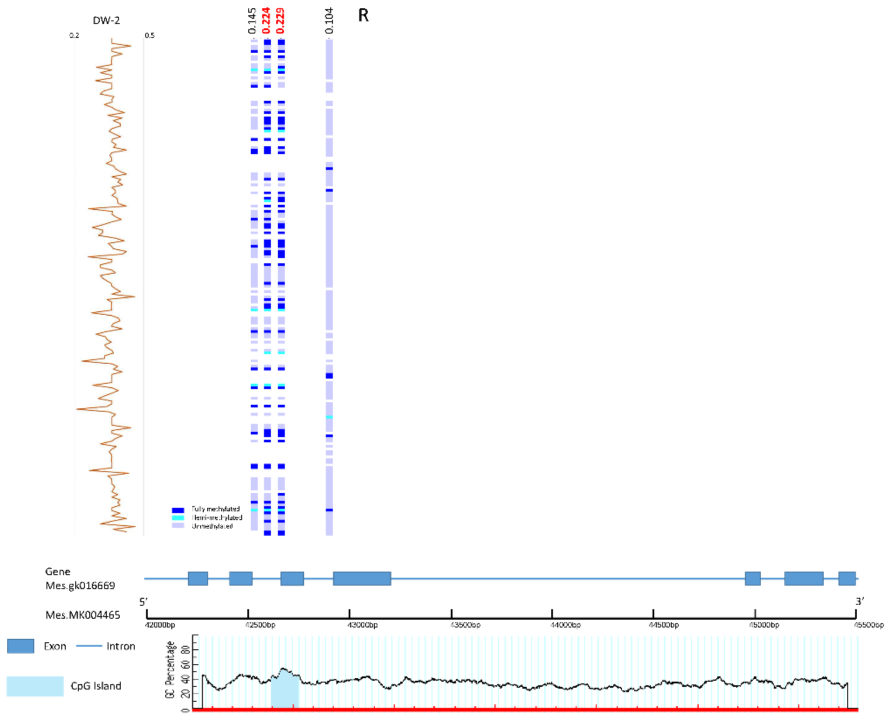
DW-2
0.2
0.5
0.145
0.224
0.229
0.104
R
Fully methylated
Hemi-methylated
Unmethylated
Gene
Mes.gk016669
Mes.MK004465
5′
3′
42000bp
42500bp
43000bp
43500bp
44000bp
44500bp
45000bp
45500bp
Exon
Intron
CpG Island
GC Percentage
0
20
40
60
80

j

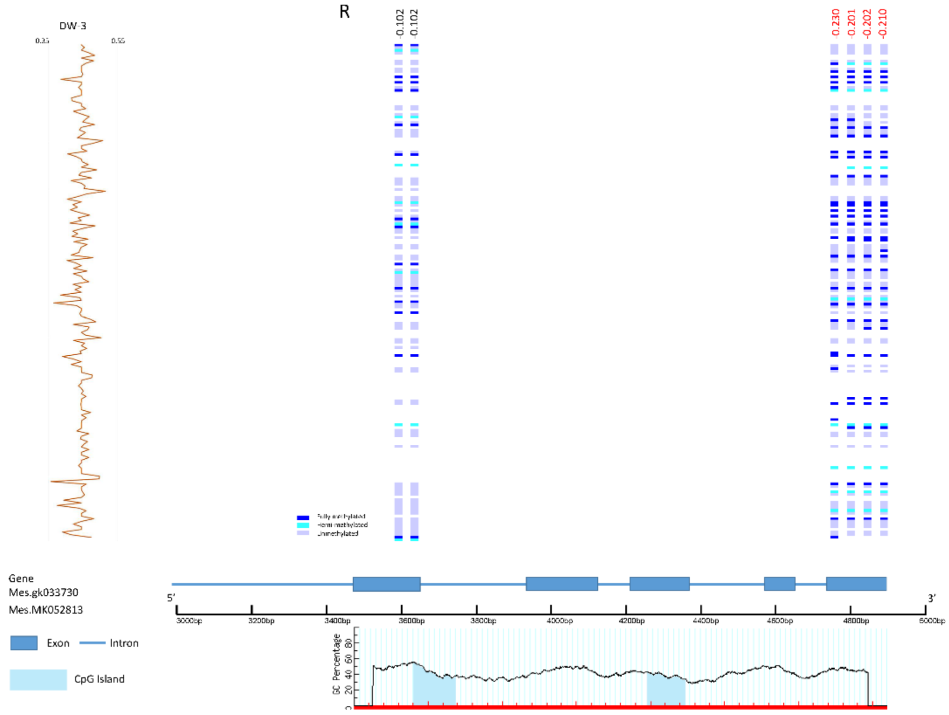
DW-3
0.35
0.55
R
0.102
0.102
0.230
0.201
-0.202
-0.210
Fully methylated
Hemi-methylated
Unmethylated
Gene
Mes.gk033730
Mes.MK052813
5′
3′
3000bp
3200bp
3400bp
3600bp
3800bp
4000bp
4200bp
4400bp
4600bp
4800bp
5000bp
Exon
Intron
CpG Island
GC Percentage
0
20
40
60
80

k

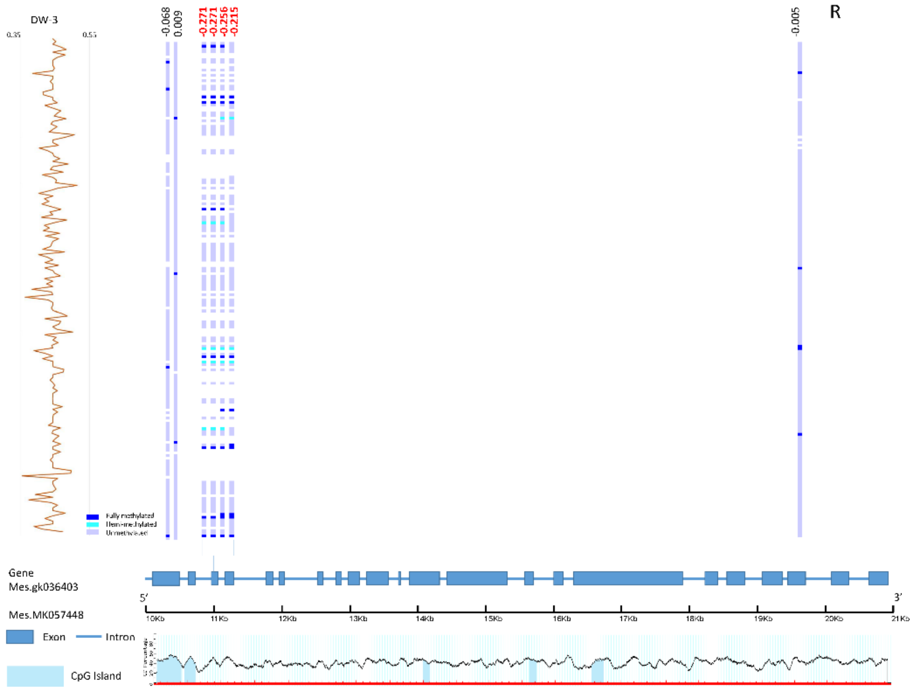

Fig. S7

DNA methylation profiles of methylated QTL genes (QTLGs^{epi}) in KS population (a, b, c, d, e, f, g, h, i, j, k,). Dark blue: fully methylation, light blue: hemi-methylation, gray: unmethylation, left: traits significantly associated with QTLGs^{epi}, y-axis indicates the number of individuals, red: the hemi-methylated or fully methylated sites significantly associated with traits based on the correlation analysis (x^2 test, p<0.01).

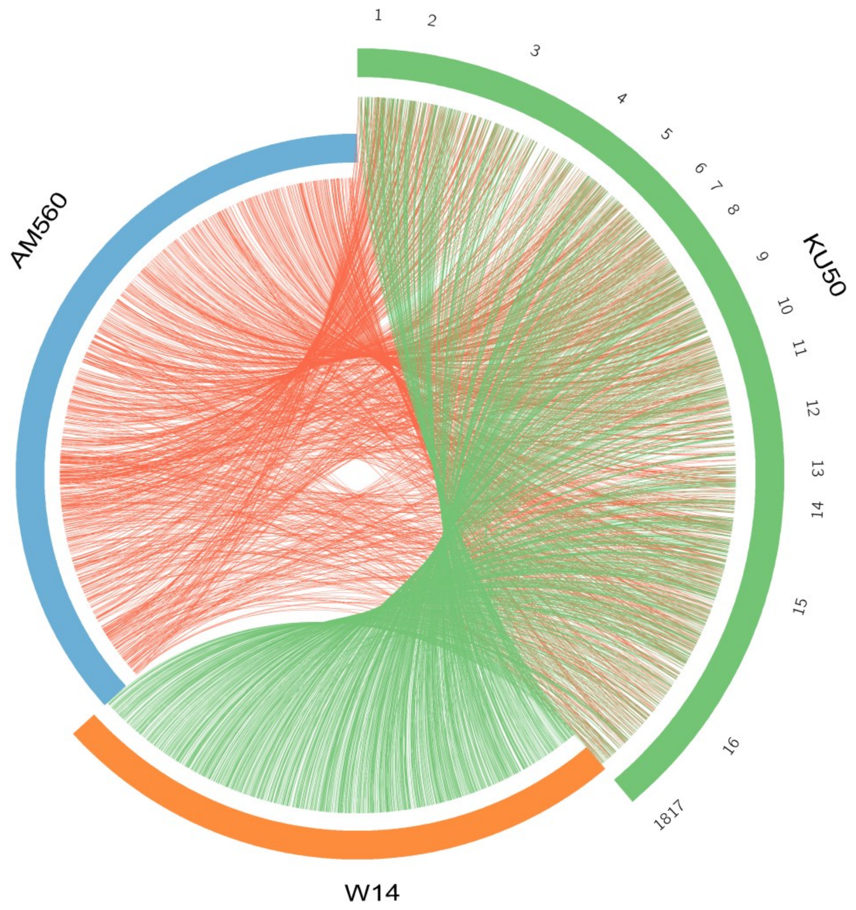

Fig. S8

A CirCOS (http://circos.ca/) figure showing synteny among three paralogous cassava genomic regions. Coloured lines connect the cassava KU50 scaffolds to AM560 and W14 scaffolds. The line distances across different scaffolds denote the similarities of the segments, with a longer line indicating a higher similarity.

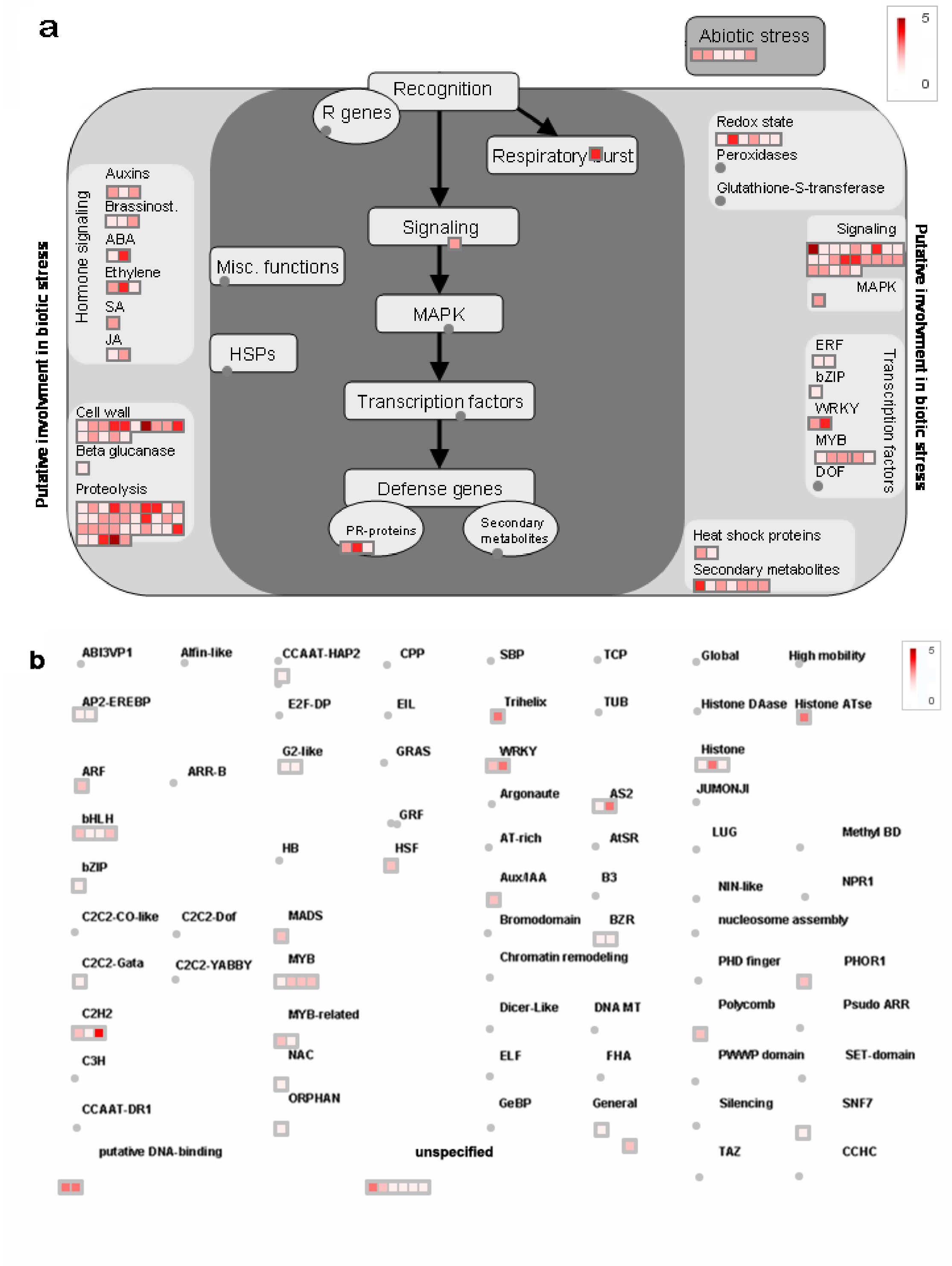
a
Abiotic stress
5
0
Recognition
R genes
Respiratory burst
Signaling
Misc. functions
MAPK
HSPs
Transcription factors
Defense genes
PR-proteins
Secondary metabolites
Putative involvment in biotic stress
Hormone signaling
Auxins
Brassinost.
ABA
Ethylene
SA
JA
Cell wall
Beta glucanase
Proteolysis
Redox state
Peroxidases
Glutathione-S-transferase
Signaling
MAPK
ERF
bZIP
WRKY
MYB
DOF
Transcription factors
Putative involvment in biotic stress
Heat shock proteins
Secondary metabolites
b
ABI3VP1
Alfin-like
CCAAT-HAP2
CPP
SBP
TCP
Global
High mobility
5
0
AP2-EREBP
E2F-DP
EIL
Trihelix
TUB
Histone DAase
Histone ATse
G2-like
GRAS
WRKY
Histone
ARF
ARR-B
Argonaute
AS2
JUMONJI
GRF
bHLH
HB
HSF
AT-rich
AtSR
LUG
Methyl BD
bZIP
Aux/IAA
B3
NIN-like
NPR1
C2C2-CO-like
C2C2-Dof
MADS
Bromodomain
BZR
nucleosome assembly
C2C2-Gata
C2C2-YABBY
MYB
Chromatin remodeling
PHD finger
PHOR1
C2H2
MYB-related
Dicer-Like
DNA MT
Polycomb
Psudo ARR
NAC
C3H
ELF
FHA
PWWP domain
SET-domain
ORPHAN
CCAAT-DR1
GeBP
General
Silencing
SNF7
putative DNA-binding
unspecified
TAZ
CCHC

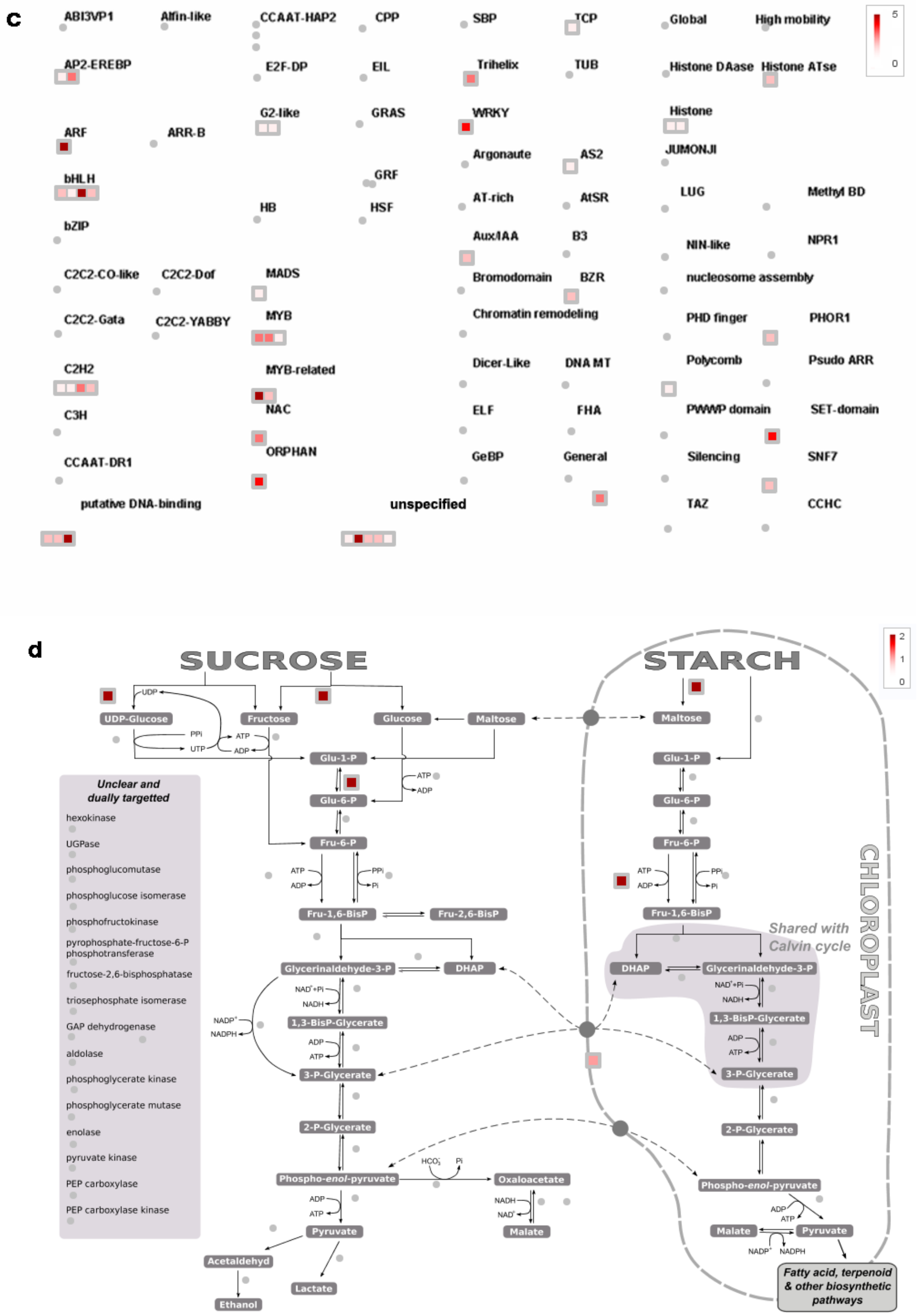
c
ABI3VP1
Alfin-like
CCAAT-HAP2
CPP
SBP
TCP
Global
High mobility
5
0
AP2-EREBP
E2F-DP
EIL
Trihelix
TUB
Histone DAase
Histone ATse
G2-like
GRAS
WRKY
Histone
ARF
ARR-B
Argonaute
AS2
JUMONJI
bHLH
GRF
AT-rich
AtSR
LUG
Methyl BD
HB
HSF
bZIP
Aux/IAA
B3
NIN-like
NPR1
C2C2-CO-like
C2C2-Dof
MADS
Bromodomain
BZR
nucleosome assembly
C2C2-Gata
C2C2-YABBY
MYB
Chromatin remodeling
PHD finger
PHOR1
C2H2
MYB-related
Dicer-Like
DNA MT
Polycomb
Psudo ARR
C3H
NAC
ELF
FHA
PWWP domain
SET-domain
CCAAT-DR1
ORPHAN
GeBP
General
Silencing
SNF7
putative DNA-binding
unspecified
TAZ
CCHC
d
SUCROSE
STARCH
2
1
0
UDP
UDP-Glucose
Fructose
Glucose
Maltose
Maltose
PPi
UTP
ATP
ADP
Glu-1-P
Glu-1-P
ATP
ADP
Glu-6-P
Glu-6-P
Fru-6-P
Fru-6-P
ATP
ADP
PPi
Pi
Fru-1,6-BisP
Fru-2,6-BisP
Fru-1,6-BisP
Unclear and dually targetted
hexokinase
UGPase
phosphoglucomutase
phosphoglucose isomerase
phosphofructokinase
pyrophosphate-fructose-6-P phosphotransferase
fructose-2,6-bisphosphatase
triosephosphate isomerase
GAP dehydrogenase
aldolase
phosphoglycerate kinase
phosphoglycerate mutase
enolase
pyruvate kinase
PEP carboxylase
PEP carboxylase kinase
Glycerinaldehyde-3-P
DHAP
DHAP
Glycerinaldehyde-3-P
Shared with Calvin cycle
CHLOROPLAST
NAD⁺+Pi
NADH
NADP⁺
NADPH
1,3-BisP-Glycerate
1,3-BisP-Glycerate
ADP
ATP
3-P-Glycerate
3-P-Glycerate
2-P-Glycerate
2-P-Glycerate
Phospho-enol-pyruvate
Phospho-enol-pyruvate
HCO₃⁻
Pi
Oxaloacetate
NADH
NAD⁺
Malate
Pyruvate
Acetaldehyd
Ethanol
Lactate
Malate
Pyruvate
NADP⁺
NADPH
Fatty acid, terpenoid & other biosynthetic pathways

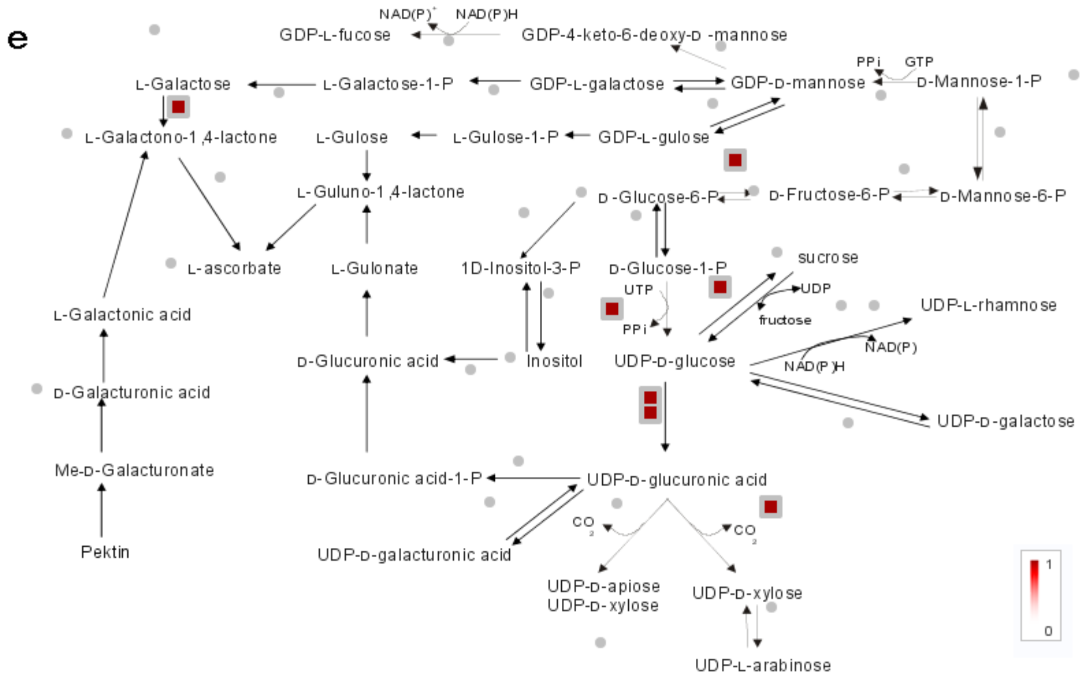

Fig. S9

Proposed cassava cold-tolerance QTL genes (CT-QTLGs) and quality and yield QTL genes (QY-QTLGs) pathways (a and b for CT-QTLGs pathways; c, d and e for QY-QTLGs pathways).

Table S1

A table of methylation patterns in SC124 and KU50 parents and their F1 progenies. Monomorphic methylation in the male and female parents and F1 progenies grouped into Class A (Class A1 for hemi-methylation, Class A2 for full methylation). Differing methylation patterns between parents, but same methylation patterns in one of the parents and F1 progenies grouped into Class B (Class B1, Class B2, Class B3 and Class B4). Same methylation patterns between parents, but different patterns between F1 progenies and each parent grouped into Class C (Class C1 and Class C2). Different patterns among F1 progenies and parents grouped into Class D (Class D1, Class D2, Class D3 and Class D4)

Pattern	Cassava SC124		Cassava KU50		F1		Number of sites
	Hemi-methylation	Fully-methylation	Hemi-methylation	Fully-methylation	Hemi-methylation	Fully-methylation	
A1	+	-	+	-	+	-	720
A2	-	+	-	+	-	+	6802
B1	+	-	-	+	+	-	0
B2	+	-	-	+	-	+	0
B3	-	+	+	-	+	-	0
B4	-	+	+	-	-	+	0
C1	+	-	+	-	-	+	0
C2	-	+	-	+	+	-	0
D1	+	-	+	-	+	+	1087
D2	-	+	-	+	+	+	62669
D3	+	-	-	+	+	+	11099
D4	-	+	+	-	+	+	36908

Table S2

Summary of GO terms over-represented in functional type of D1 methylation of cassava

Term_type[a]	*GO term*[b]	*Number (%) in group*	*Number (%) in background*[c]
1 (C)	└ nuclear ubiquitin ligase complex,	1/21 4.7%	10/27594 0.0%
	└ anaphase-promoting complex,	1/21 4.7%	7/27594 0.0%
	└ proteasome regulatory particle, base subcomplex,	1/21 4.7%	9/27594 0.0%
	└ organelle,	10/21 47.6%	6091/27594 22.0%
	└ nuclear part,	3/21 14.2%	459/27594 1.6%
	└ intracellular organelle,	10/21 47.6%	6090/27594 22.0%
	└ nuclear chromatin,	1/21 4.7%	8/27594 0.0%
	└ euchromatin,	1/21 4.7%	4/27594 0.0%
	└ nuclear euchromatin,	1/21 4.7%	3/27594 0.0%
	└ Cajal body,	1/21 4.7%	7/27594 0.0%
	└ cytosol,	3/21 14.2%	504/27594 1.8%
	└ intracellular,	12/21 57.1%	7208/27594 26.1%
	└ intracellular part,	12/21 57.1%	6908/27594 25.0%
2 (F)	└ GDP-dissociation inhibitor activity,	1/21 4.7%	7/27594 0.0%
	└ Rab GDP-dissociation inhibitor activity,	1/21 4.7%	4/27594 0.0%
	└ catalytic activity,	14/21 66.6%	7553/27594 27.3%
	└ tRNA dihydrouridine synthase activity,	1/21 4.7%	4/27594 0.0%
	└ nucleotidyltransferase activity,	2/21 9.5%	154/27594 0.5%
	└ glucose-1-phosphate adenylyltransferase activity,	1/21 4.7%	6/27594 0.0%
	└ hydrolase activity, acting on acid anhydrides,	5/21 23.8%	688/27594 2.4%
	└ hydrolase activity, acting on acid anhydrides, in phosphorus-containing anhydrides,	5/21 23.8%	681/27594 2.4%
	└ pyrophosphatase activity,	5/21 23.8%	679/27594 2.4%
	└ nucleoside-triphosphatase activity,	4/21 19.0%	651/27594 2.3%
	└ helicase activity,	2/21 9.5%	160/27594 0.5%
	└ nucleotide diphosphatase activity,	1/21 4.7%	8/27594 0.0%
	└ bis(5'-adenosyl)-pentaphosphatase activity,	1/21 4.7%	3/27594 0.0%
	└ bis(5'-nucleosyl)-tetraphosphatase activity,	1/21 4.7%	3/27594 0.0%
	└ bis(5'-nucleosyl)-tetraphosphatase (asymmetrical) activity,	1/21 4.7%	3/27594 0.0%
	└ leucine-tRNA ligase activity,	1/21 4.7%	2/27594 0.0%
	└ binding,	13/21 61.9%	9513/27594 34.4%

	└ nucleotide binding,	7/21 33.3%	2085/27594 7.5%
	└ purine nucleotide binding,	6/21 28.5%	1601/27594 5.8%
	└ adenyl nucleotide binding,	6/21 28.5%	1387/27594 5.0%
	└ adenyl ribonucleotide binding,	5/21 23.8%	1314/27594 4.7%
	└ ATP binding,	5/21 23.8%	1311/27594 4.7%
	└ purine ribonucleotide binding,	5/21 23.8%	1524/27594 5.5%
	└ ribonucleotide binding,	5/21 23.8%	1524/27594 5.5%
	└ nucleoside binding,	6/21 28.5%	1393/27594 5.0%
	└ purine nucleoside binding,	6/21 28.5%	1389/27594 5.0%
	└ siRNA binding,	1/21 4.7%	4/27594 0.0%
3 (P)	└ chromatin organization,	2/21 9.5%	160/27594 0.5%
	└ chromatin modification,	2/21 9.5%	86/27594 0.3%
	└ histone H3-K9 methylation,	1/21 4.7%	6/27594 0.0%
	└ chloroplast relocation,	1/21 4.7%	9/27594 0.0%
	└ establishment of plastid localization,	1/21 4.7%	9/27594 0.0%
	└ establishment of organelle localization,	1/21 4.7%	12/27594 0.0%
	└ plastid localization,	1/21 4.7%	10/27594 0.0%
	└ chloroplast avoidance movement,	1/21 4.7%	5/27594 0.0%
	└ chloroplast accumulation movement,	1/21 4.7%	4/27594 0.0%
	└ positive regulation of DNA metabolic process,	1/21 4.7%	4/27594 0.0%
	└ regulation of DNA metabolic process,	2/21 9.5%	26/27594 0.0%
	└ regulation of DNA repair,	1/21 4.7%	4/27594 0.0%
	└ positive regulation of DNA repair,	1/21 4.7%	1/27594 0.0%
	└ chromatin silencing by small RNA,	1/21 4.7%	4/27594 0.0%
	└ regulation of DNA endoreduplication,	1/21 4.7%	11/27594 0.0%
	└ regulation of DNA-dependent DNA replication,	1/21 4.7%	12/27594 0.0%
	└ regulation of gibberellic acid mediated signaling pathway,	1/21 4.7%	13/27594 0.0%
	└ positive regulation of gibberellic acid mediated signaling pathway,	1/21 4.7%	4/27594 0.0%
	└ positive regulation of cellular process,	2/21 9.5%	137/27594 0.4%
	└ positive regulation of response to stimulus,	2/21 9.5%	57/27594 0.2%
	└ regulation of response to stimulus,	2/21 9.5%	157/27594 0.5%
	└ metabolic process,	11/21 52.3%	6834/27594 24.7%
	└ macromolecule metabolic process,	9/21 42.8%	4086/27594 14.8%
	└ cellular macromolecule metabolic process,	9/21 42.8%	3667/27594 13.2%
	└ RNA metabolic process,	3/21 14.2%	510/27594 1.8%

	└ ncRNA metabolic process,	2/21 9.5%	198/27594 0.7%
	└ tRNA metabolic process,	2/21 9.5%	98/27594 0.3%
	└ DNA metabolic process,	3/21 14.2%	311/27594 1.1%
	└ somatic cell DNA recombination,	1/21 4.7%	4/27594 0.0%
	└ nucleobase, nucleoside, nucleotide and nucleic acid metabolic process,	5/21 23.8%	1104/27594 4.0%
	└ nucleic acid metabolic process,	5/21 23.8%	866/27594 3.1%
	└ primary metabolic process,	10/21 47.6%	5719/27594 20.7%
	└ raffinose family oligosaccharide biosynthetic process,	1/21 4.7%	4/27594 0.0%
	└ nitrogen compound metabolic process,	5/21 23.8%	1548/27594 5.6%
	└ cellular nitrogen compound metabolic process,	5/21 23.8%	1484/27594 5.3%
	└ cellular process,	12/21 57.1%	7393/27594 26.7%
	└ organelle organization,	3/21 14.2%	526/27594 1.9%

[a] P=Biological Process, F=Molecular Function; C=Cellular Component [b] GO terms best describing all branches of the network are selected;

[c] GO annotation of *A. thaliana* (TAIR10, n= 27,594) was used as the background.

Table S4

Annotation of significantly differential methylation and differentially expressed genes in Cassava (DMR-DEGs) with BiNGO[19] (P<0.1, χ2 text) compared with cassava KU50 genes[14].

Tterm_type[a]	GO term[b]	Number % in group	Number % in background[c]
1(C)	└ ribonucleoprotein complex	1/14 7.1%	439/27594 1.5%
	└ small nucleolar ribonucleoprotein complex	1/14 7.1%	97/27594 0.3%
	└ organelle	5/14 35.7%	6091/27594 22.0%
	└ membrane-bounded organelle	5/14 35.7%	5767/27594 20.8%
	└ intracellular membrane-bounded organelle	5/14 35.7%	5766/27594 20.8%
	└ nucleus	2/14 14.2%	2308/27594 8.3%
	└ vacuole	2/14 14.2%	455/27594 1.6%
	└ intracellular organelle	5/14 35.7%	6090/27594 22.0%
	└ membrane	3/14 21.4%	3727/27594 13.5%
	└ plasma membrane	3/14 21.4%	1574/27594 5.7%
	└ cytosol	1/14 7.1%	504/27594 1.8%
	└ cytoplasm	4/14 28.5%	4745/27594 17.1%
	└ cytoplasmic part	4/14 28.5%	4323/27594 15.6%
	└ intracellular	6/14 42.8%	7208/27594 26.1%
	└ intracellular part	6/14 42.8%	6908/27594 25.0%
	└ extracellular region	1/14 7.1%	393/27594 1.4%
	└ apoplast	1/14 7.1%	286/27594 1.0%
2(F)	└ catalytic activity	5/14 35.7%	7553/27594 27.3%
	└ hydrolase activity, acting on carbon-nitrogen (but not peptide) bonds	1/14 7.1%	104/27594 0.3%
	└ hydrolase activity, acting on carbon-nitrogen (but not peptide) bonds, in linear amides	1/14 7.1%	58/27594 0.2%
	└ beta-ureidopropionase activity	1/14 7.1%	1/27594 0.0%
	└ hydrolase activity, acting on acid anhydrides	1/14 7.1%	688/27594 2.4%
	└ hydrolase activity, acting on acid anhydrides, in phosphorus-containing anhydrides	1/14 7.1%	681/27594 2.4%
	└ pyrophosphatase activity	1/14 7.1%	679/27594 2.4%
	└ nucleoside-triphosphatase activity	1/14 7.1%	651/27594 2.3%
	└ ATPase activity	1/14 7.1%	328/27594 1.1%
	└ isomerase activity	1/14 7.1%	198/27594 0.7%
	└ cis-trans isomerase activity	1/14 7.1%	57/27594 0.2%
	└ peptidyl-prolyl cis-trans isomerase activity	1/14 7.1%	56/27594 0.2%
	└ nucleic acid binding	3/14 21.4%	3806/27594 13.7%
	└ translation factor activity, nucleic acid binding	1/14 7.1%	124/27594 0.4%
	└ translation initiation factor activity	1/14 7.1%	83/27594 0.3%
3(P)	└ reproduction	2/14 14.2%	931/27594 3.3%
	└ reproductive process	2/14 14.2%	911/27594 3.3%
	└ reproductive developmental process	2/14 14.2%	829/27594 3.0%

└ vegetative to reproductive phase transition of meristem	1/14 7.1%	86/27594 0.3%
└ photoperiodism, flowering	1/14 7.1%	36/27594 0.1%
└ reproductive structure development	2/14 14.2%	735/27594 2.6%
└ fruit development	1/14 7.1%	459/27594 1.6%
└ seed development	1/14 7.1%	438/27594 1.5%
└ embryonic development ending in seed dormancy	1/14 7.1%	372/27594 1.3%
└ multicellular organismal process	3/14 21.4%	1732/27594 6.2%
└ multicellular organismal development	3/14 21.4%	1655/27594 5.9%
└ system development	1/14 7.1%	720/27594 2.6%
└ embryonic development	1/14 7.1%	429/27594 1.5%
└ post-embryonic development	2/14 14.2%	884/27594 3.2%
└ developmental process	3/14 21.4%	1820/27594 6.5%
└ anatomical structure development	3/14 21.4%	1392/27594 5.0%
└ organ development	1/14 7.1%	719/27594 2.6%
└ shoot system development	1/14 7.1%	304/27594 1.1%
└ shoot development	1/14 7.1%	300/27594 1.0%
└ leaf morphogenesis	1/14 7.1%	117/27594 0.4%
└ phyllome development	1/14 7.1%	222/27594 0.8%
└ leaf development	1/14 7.1%	201/27594 0.7%
└ cell differentiation	1/14 7.1%	296/27594 1.0%
└ anatomical structure morphogenesis	1/14 7.1%	515/27594 1.8%
└ shoot morphogenesis	1/14 7.1%	157/27594 0.5%
└ organ morphogenesis	1/14 7.1%	178/27594 0.6%
└ response to stimulus	3/14 21.4%	3207/27594 11.6%
└ response to inorganic substance	1/14 7.1%	434/27594 1.5%
└ response to metal ion	1/14 7.1%	350/27594 1.2%
└ response to cadmium ion	1/14 7.1%	279/27594 1.0%
└ response to external stimulus	1/14 7.1%	229/27594 0.8%
└ cellular response to external stimulus	1/14 7.1%	128/27594 0.4%
└ response to extracellular stimulus	1/14 7.1%	140/27594 0.5%
└ response to nutrient levels	1/14 7.1%	126/27594 0.4%
└ cellular response to nutrient levels	1/14 7.1%	114/27594 0.4%
└ cellular response to nitrogen levels	1/14 7.1%	17/27594 0.0%
└ response to radiation	1/14 7.1%	471/27594 1.7%
└ response to light stimulus	1/14 7.1%	455/27594 1.6%
└ photoperiodism	1/14 7.1%	39/27594 0.1%
└ ion transport	1/14 7.1%	345/27594 1.2%
└ ion transmembrane transport	1/14 7.1%	40/27594 0.1%
└ cation transport	1/14 7.1%	271/27594 0.9%
└ monovalent inorganic cation transport	1/14 7.1%	105/27594 0.3%
└ proton transport	1/14 7.1%	42/27594 0.1%
└ energy coupled proton transport, down electrochemical gradient	1/14 7.1%	33/27594 0.1%
└ hydrogen transport	1/14 7.1%	42/27594 0.1%

└ regulation of developmental process	1/14 7.1%	273/27594 0.9%
└ regulation of development, heterochronic	1/14 7.1%	33/27594 0.1%
└ positive regulation of development, heterochronic	1/14 7.1%	8/27594 0.0%
└ metabolic process	5/14 35.7%	6834/27594 24.7%
└ macromolecule metabolic process	3/14 21.4%	4086/27594 14.8%
└ translational initiation	1/14 7.1%	63/27594 0.2%
└ protein metabolic process	3/14 21.4%	3147/27594 11.4%
└ proteolysis	1/14 7.1%	614/27594 2.2%
└ cellular metabolic process	4/14 28.5%	5407/27594 19.5%
└ cellular nitrogen compound catabolic process	1/14 7.1%	23/27594 0.0%
└ nucleobase, nucleoside and nucleotide catabolic process	1/14 7.1%	7/27594 0.0%
└ nucleobase catabolic process	1/14 7.1%	4/27594 0.0%
└ pyrimidine base catabolic process	1/14 7.1%	3/27594 0.0%
└ uracil catabolic process	1/14 7.1%	3/27594 0.0%
└ cellular nitrogen compound biosynthetic process	1/14 7.1%	378/27594 1.3%
└ nucleobase, nucleoside and nucleotide biosynthetic process	1/14 7.1%	112/27594 0.4%
└ nucleotide biosynthetic process	1/14 7.1%	99/27594 0.3%
└ purine nucleotide biosynthetic process	1/14 7.1%	74/27594 0.2%
└ nucleoside triphosphate biosynthetic process	1/14 7.1%	61/27594 0.2%
└ ribonucleotide biosynthetic process	1/14 7.1%	75/27594 0.2%
└ purine ribonucleotide biosynthetic process	1/14 7.1%	68/27594 0.2%
└ ribonucleoside triphosphate biosynthetic process	1/14 7.1%	60/27594 0.2%
└ nucleobase, nucleoside, nucleotide and nucleic acid metabolic process	2/14 14.2%	1104/27594 4.0%
└ nucleobase, nucleoside, nucleotide and nucleic acid biosynthetic process	1/14 7.1%	112/27594 0.4%
└ nucleobase, nucleoside, nucleotide and nucleic acid catabolic process	1/14 7.1%	7/27594 0.0%
└ nucleobase, nucleoside and nucleotide metabolic process	2/14 14.2%	182/27594 0.6%
└ heterocycle catabolic process	1/14 7.1%	28/27594 0.1%
└ heterocycle metabolic process	2/14 14.2%	340/27594 1.2%
└ purine nucleotide metabolic process	1/14 7.1%	79/27594 0.2%
└ purine nucleoside triphosphate biosynthetic process	1/14 7.1%	60/27594 0.2%
└ purine ribonucleoside triphosphate biosynthetic process	1/14 7.1%	60/27594 0.2%
└ ATP biosynthetic process	1/14 7.1%	59/27594 0.2%
└ purine nucleoside triphosphate metabolic process	1/14 7.1%	60/27594 0.2%
└ purine ribonucleoside triphosphate metabolic process	1/14 7.1%	60/27594 0.2%

└ ATP metabolic process	1/14 7.1%	59/27594 0.2%
└ purine ribonucleotide metabolic process	1/14 7.1%	72/27594 0.2%
└ pyrimidine base metabolic process	1/14 7.1%	6/27594 0.0%
└ uracil metabolic process	1/14 7.1%	3/27594 0.0%
└ generation of precursor metabolites and energy	1/14 7.1%	199/27594 0.7%
└ oxidative phosphorylation	1/14 7.1%	54/27594 0.1%
└ cellular aromatic compound metabolic process	1/14 7.1%	296/27594 1.0%
└ nucleobase metabolic process	1/14 7.1%	18/27594 0.0%
└ ATP synthesis coupled proton transport	1/14 7.1%	33/27594 0.1%
└ primary metabolic process	5/14 35.7%	5719/27594 20.7%
└ small molecule metabolic process	2/14 14.2%	1248/27594 4.5%
└ nucleoside phosphate metabolic process	1/14 7.1%	145/27594 0.5%
└ nucleotide metabolic process	1/14 7.1%	145/27594 0.5%
└ nucleoside triphosphate metabolic process	1/14 7.1%	61/27594 0.2%
└ ribonucleoside triphosphate metabolic process	1/14 7.1%	60/27594 0.2%
└ ribonucleotide metabolic process	1/14 7.1%	81/27594 0.2%
└ nitrogen compound metabolic process	2/14 14.2%	1548/27594 5.6%
└ cellular nitrogen compound metabolic process	2/14 14.2%	1484/27594 5.3%
└ catabolic process	1/14 7.1%	660/27594 2.3%
└ cellular catabolic process	1/14 7.1%	513/27594 1.8%
└ small molecule catabolic process	1/14 7.1%	163/27594 0.5%
└ cellular biosynthetic process	2/14 14.2%	2444/27594 8.8%
└ small molecule biosynthetic process	1/14 7.1%	622/27594 2.2%
└ cellular process	5/14 35.7%	7393/27594 26.7%
└ cellular developmental process	1/14 7.1%	435/27594 1.5%
└ cellular response to stimulus	1/14 7.1%	729/27594 2.6%
└ transmembrane transport	1/14 7.1%	79/27594 0.2%
└ cell communication	1/14 7.1%	206/27594 0.7%
└ cellular response to extracellular stimulus	1/14 7.1%	128/27594 0.4%

[a] P=Biological Process, F=Molecular Function; C=Cellular Component [b] GO terms best describing all branches of the network are selected; [c] GO annotation of All Methylation Gene (n= 6905) was used as the background.

Table S6

Candidate key QTLs^{epi} for cold stress

CT-QTLs^{epi}	CTIG	CTIF-1	CTIF-2	CTIF-3	CTIF-4	CTIF-5	LFIF-1	LFIF-2	LFIF-3	RIG	RIF-1	RIF-2	rSRY-1	rSRY-2
Mes.MK003417_7870	-	-	-	-	-	-	-	-	-	-	-	-	-	-
Mes.MK004280_11693	-	-	-	-	0.18	-	-	0.31	-	-	-	-	-	-
Mes.MK004280_11707	-	-	-	-	0.18	-	-	0.31	-	-	-	-	-	-
Mes.MK004280_11721	-	-	-	-	0.18	-	-	0.31	-	-	-	-	-	-
Mes.MK004280_11734	-	-	-	-	0.18	-	-	0.31	-	-	-	-	-	-
Mes.MK004524_1832	-	-	-	-	-	-	0.18	0.19	-	-	-	-	-	-
Mes.MK004547_1083	-	-	-	-	-	-	0.20	0.15	-	-	-	-	-	-
Mes.MK004830_1645	-	-	-	-	-	-	0.24	0.16	-	-	-	-	-	-
Mes.MK004830_1664	-	-	-	-	-	-	0.20	0.20	-	-	-	-	-	-
Mes.MK004830_1680	-	-	-	-	-	-	0.22	0.19	-	-	-	-	-	-
Mes.MK004830_1694	-	-	-	-	-	-	0.21	0.19	-	-	-	-	-	-
Mes.MK004830_1707	-	-	-	-	-	-	0.16	0.17	-	-	-	-	-	-
Mes.MK004830_1713	-	-	-	-	-	-	0.16	0.15	-	-	-	-	-	-
Mes.MK004830_1732	-	-	-	-	-	-	0.14	0.15	-	0.14	-	-	-	-
Mes.MK004880_421	-	-	-	-	-	-	-	-	-	-	-	-	-	-
Mes.MK004880_444	-	-	-	-	-	-	-	-	-	-	-	-	-	-
Mes.MK004880_454	-	-	-	-	-	-	-	-	-	-	-	-	0.16	-
Mes.MK004880_460	-	-	-	-	-	-	-	-	-	-	-	-	-	-
Mes.MK004880_471	-	-	-	-	-	-	-	-	-	-	-	-	-	-
Mes.MK005211_12464	-	-	-	-	-	-	0.17	0.19	-	-	-	-	-	-
Mes.MK005722_3737	-	-	-	-	-	-	-	-	-	0.15	-	-	-	-
Mes.MK005722_3805	-	-	-	-	-	-	-	-	-	0.19	0.16	-	-	-
Mes.MK005869_11660	-	-	-	-	-	-	0.16	0.16	-	-	-	-	-	-
Mes.MK006708_18367	-	-	-	-	-	-	0.21	0.15	-	-	-	-	-	-
Mes.MK006871_1121	-	-	-	-	-	-	-	0.14	-	-	-	-	-	-
Mes.MK006871_1170	-	-	-	-	-	-	-	0.19	-	-	-	-	-	-
Mes.MK006871_1186	-	-	-	-	-	-	0.12	0.21	-	-	-	-	-	-
Mes.MK006871_1195	-	-	-	-	-	-	0.13	0.20	-	-	-	-	-	-
Mes.MK006871_1206	-	-	-	-	-	-	0.13	0.20	-	-	-	-	-	-
Mes.MK007333_2075	0.20	-	-	-	-	-	-	-	-	-	-	-	-	-
Mes.MK007333_2083	0.20	-	-	-	-	-	-	-	-	-	-	-	-	-
Mes.MK007397_2954	-	-	-	-	-	-	-	-	-	-	-	-	-	-
Mes.MK009561_9567	-	-	-	-	-	-	-	-	-	-	-	-	-	-
Mes.MK017953_6802	-	-	-	-	-	-	-	-	-	-	-	-	-	-
Mes.MK017953_6828	-	-	-	-	-	-	-	-	-	-	-	-	-	-
Mes.MK017953_6834	-	-	-	-	-	-	-	-	-	-	-	-	-	-
Mes.MK017953_6866	-	-	-	-	-	-	-	-	-	-	-	-	-	-
Mes.MK017953_6879	-	-	-	-	-	-	-	-	-	-	-	-	-	-
Mes.MK022990_6335	-	-	-	-	-	-	-	-	-	-	-	-	-	-
Mes.MK022990_6346	-	-	-	-	-	-	-	-	-	-	-	-	-	-
Mes.MK022990_6366	-	-	-	-	-	-	-	-	-	-	-	-	-	-
Mes.MK022990_6382	-	-	-	-	-	-	-	-	-	-	-	-	-	-
Mes.MK033815_102	-	-	-	-	-	-	0.22	-	0.17	-	-	-	-	-
Mes.MK033815_111	-	-	-	-	-	-	0.22	-	0.17	-	-	-	-	-
Mes.MK039662_3566	-	-	-	-	-	-	-	-	-	-	-	-	-	-
Mes.MK039662_3592	-	-	-	-	-	-	-	-	-	-	-	-	-	-
Mes.MK039662_825	-	-	-	-	-	-	-	-	-	-	-	-	-	0.22
Mes.MK039662_907	-	-	-	-	-	-	-	-	-	-	-	-	-	0.18
Mes.MK041351_6728	-	-	-	-	-	-	-	-	-	-	-	-	-	-
Mes.MK041406_4836	-	-	-	-	-	-	-	-	-	-	-	-	-	-
Mes.MK041406_4850	-	-	-	-	-	-	-	-	-	-	-	-	-	-
Mes.MK041406_4862	-	-	-	-	-	-	-	-	-	-	-	-	-	-
Mes.MK046277_871	-	-	-	-	-	-	-	-	-	-	-	-	-	-
Mes.MK046277_882	-	-	-	-	-	-	-	-	-	-	-	-	-	-

Mes.MK046277_892	-	-	-	-	-	-	-	-	-	-	-	-	-	-
Mes.MK046277_904	-	-	-	-	-	-	-	-	-	-	-	-	-	-
Mes.MK046277_930	-	-	-	-	-	-	-	-	-	-	-	-	-	-
Mes.MK046277_942	-	-	-	-	-	-	-	-	-	-	-	-	-	-
Mes.MK055282_12519	-	-	-	-	-	-	-	-	-	-	-	-	-	-
Mes.MK055588_5010	-	-	-	-	-	-	-	-	-	-	-	-	0.17	-
Mes.MK055588_5080	-	-	-	-	-	-	-	-	-	-	-	-	-	-
Mes.MK055975_19318	-	-	-	-	-	-	-	-	-	-	-	-	-	-
Mes.MK055975_19339	-	-	-	-	-	-	-	-	-	-	-	-	-	-
Mes.MK055975_19390	-	-	-	-	-	-	-	-	-	-	-	-	-	-
Mes.MK056600_17332	-	-	-	-	-	-	0.19	0.28	-	-	-	-	-	-
Mes.MK059216_610	-	-	-	-	-	-	-	-	-	-	-	-	-	-
Mes.MK059216_622	-	-	-	-	-	-	-	-	-	-	-	-	-	-
Mes.MK059216_651	-	-	-	-	-	-	-	-	-	-	-	-	-	-
Mes.MK059216_666	-	-	-	-	-	-	-	-	-	-	-	-	-	-
Mes.MK059216_670	-	-	-	-	-	-	-	-	-	-	-	-	-	-
Mes.MK059216_679	-	-	-	-	-	-	-	-	-	-	-	-	-	-
Mes.MK059216_686	-	-	-	-	-	-	-	-	-	-	-	-	-	-
Mes.MK059216_699	-	-	-	-	-	-	-	-	-	-	-	-	-	-
Mes.MK059216_702	-	-	-	-	-	-	-	-	-	-	-	-	-	-
Mes.MK060675_443	-	-	-	-	-	-	-	-	-	-	-	-	-	-
Mes.MK061119_388	-	-	-	-	-	-	-	-	-	-	-	-	-	-

Table S7

Candidate key QTLs^{epi} for storage root quality and yield

QY-QTLs^{epi}	SRY-1	SRY-2	SRY-3	SRY-4	SRY-5	NSR-1	NSR-2	NSR-3	DW-1	DW-2	DW-3	DW-4	FSC-1	FSC-2
Mes.MK000113_6033	-	-	-	-	-	-	-	-	-	0.21	-	-	0.19	0.21
Mes.MK000201_1840	-	-	-	-	-	-	-	-	-	-	-	-	-	-
Mes.MK000201_1843	-	-	-	-	-	-	-	-	-	-	-	-	-	-
Mes.MK000201_1845	-	-	-	-	-	-	-	-	-	-	-	-	-	-
Mes.MK000201_1848	-	-	-	-	-	-	-	-	-	-	-	-	-	-
Mes.MK000201_1851	-	-	-	-	-	-	-	-	-	-	-	-	-	-
Mes.MK000378_4605	-	-	-	-	-	-	-	-	-	-	-	-	-	-
Mes.MK000378_4648	-	-	-	-	-	-	-	-	-	-	-	-	-	-
Mes.MK000378_4654	-	-	-	-	-	-	-	-	-	-	-	-	-	-
Mes.MK000378_4665	-	-	-	-	-	-	-	-	-	-	-	-	-	-
Mes.MK000378_4672	-	-	-	-	-	-	-	-	-	-	-	-	-	-
Mes.MK000378_4683	-	-	-	-	-	-	-	-	-	-	-	-	-	-
Mes.MK000378_4725	-	-	-	-	-	-	-	-	-	-	-	-	-	-
Mes.MK000378_4735	-	-	-	-	-	-	-	-	-	-	-	-	-	0.18
Mes.MK000602_8248	-	0.17	-	-	0.19	-	-	-	-	-	0.21	-	-	-
Mes.MK000602_8272	-	0.19	-	-	0.22	-	-	-	-	-	0.18	-	-	-
Mes.MK000602_8278	-	0.19	-	-	0.22	-	-	-	-	-	-	-	-	-
Mes.MK000602_8290	-	0.18	-	-	0.22	-	-	-	-	-	-	-	-	-
Mes.MK000602_8312	-	0.18	-	-	0.21	-	-	-	-	-	0.18	-	-	-
Mes.MK002388_6810	-	-	-	-	-	-	-	-	-	0.18	-	-	0.20	0.19
Mes.MK003284_2312	-	-	-	-	-	-	-	-	-	-	-	-	-	-
Mes.MK003284_2313	-	-	-	-	-	-	-	-	-	-	-	-	-	-
Mes.MK004465_4255	-	-	-	-	-	-	-	-	-	0.22	-	-	0.17	-
Mes.MK004465_4260	-	-	-	-	-	-	-	-	-	0.22	-	-	0.17	-
Mes.MK004546_3372	-	-	-	-	-	-	-	-	0.19	-	0.20	-	-	-
Mes.MK004583_1994	-	-	-	-	-	-	0.19	0.18	-	-	-	-	-	-
Mes.MK004623_2810	-	-	-	-	-	-	-	-	0.15	-	0.16	-	-	-
Mes.MK004833_1003	-	-	-	-	-	-	-	-	-	0.18	-	0.18	0.16	0.18
Mes.MK004833_1009	-	-	-	-	-	-	-	-	-	0.18	-	0.18	0.16	0.18

Mes.MK004833_1029	-	-	-	-	-	-	-	-	-	0.18	-	0.18	0.16	0.18
Mes.MK006989_7434	-	-	-	-	-	-	-	-	-	-	0.18	-	-	-
Mes.MK007844_1333	-	-	-	-	-	-	0.17	0.18	-	-	-	-	-	-
Mes.MK008209_238	-	-	-	-	-	-	-	-	-	-	-	-	-	-
Mes.MK012961_1065	-	-	0.21	-	-	-	0.18	0.17	-	-	-	-	-	-
Mes.MK016403_4977	-	-	-	-	-	-	-	-	-	-	-	-	-	-
Mes.MK020863_428	-	-	-	-	-	-	-	-	-	-	-	-	-	-
Mes.MK020863_438	-	-	-	-	-	-	-	0.16	-	-	-	-	-	-
Mes.MK022148_2364	-	-	-	-	-	-	-	-	-	-	-	-	-	-
Mes.MK026403_2572	-	-	-	-	-	-	-	-	-	-	-	-	-	-
Mes.MK026403_2583	-	-	-	-	-	-	-	-	-	-	-	-	-	-
Mes.MK026403_2645	-	-	-	-	-	-	-	-	-	-	-	-	-	-
Mes.MK026403_2654	-	-	-	-	-	-	-	-	-	-	-	-	-	-
Mes.MK030793_4457	-	-	0.22	-	-	-	0.17	0.22	-	-	-	-	-	-
Mes.MK031682_1212	-	-	-	-	-	-	-	-	0.18	-	0.16	-	-	-
Mes.MK039816_638	-	-	-	-	-	-	-	-	-	-	-	-	-	-
Mes.MK039816_699	-	-	-	-	-	-	-	-	-	-	-	-	-	-
Mes.MK039816_714	-	-	-	-	-	-	-	-	-	-	-	-	-	-
Mes.MK042185_523	-	-	-	-	-	-	-	-	0.19	-	0.17	-	-	-
Mes.MK042185_546	-	-	-	-	-	-	-	-	0.19	-	0.17	-	-	-
Mes.MK042185_556	-	-	-	-	-	-	-	-	0.19	-	0.17	-	-	-
Mes.MK042791_343	-	-	-	-	-	-	-	-	-	-	-	-	-	-
Mes.MK045068_1167	-	0.17	-	-	-	0.16	0.16	-	-	-	-	-	-	-
Mes.MK046256_8986	-	-	-	-	-	-	-	-	0.20	-	0.19	-	-	-
Mes.MK046256_8993	-	-	-	-	-	-	-	-	0.18	-	0.18	-	-	-
Mes.MK046256_9000	-	-	-	-	-	-	-	-	0.20	-	0.20	-	-	-
Mes.MK046277_871	-	-	-	-	-	-	-	-	-	-	-	-	-	-
Mes.MK046277_882	-	-	-	-	-	-	-	-	-	-	-	-	-	-
Mes.MK046277_892	-	-	-	-	-	-	-	-	-	-	-	-	-	-
Mes.MK046277_904	-	-	-	-	-	-	-	-	-	-	-	-	-	-
Mes.MK046277_930	-	-	-	-	-	-	-	-	-	-	-	-	-	-
Mes.MK046277_942	-	-	-	-	-	-	-	-	-	-	-	-	-	-
Mes.MK046277_968	-	-	-	-	-	-	-	-	-	-	-	-	-	-
Mes.MK046277_976	-	-	-	-	-	-	-	-	-	-	-	-	-	-
Mes.MK049007_3587	-	-	-	-	-	-	-	-	0.20	-	-	0.14	-	-
Mes.MK049783_1917	-	-	-	-	-	-	-	-	-	-	0.17	0.17	-	0.18
Mes.MK050954_1701	-	-	-	-	-	-	-	-	0.19	-	0.17	-	-	-
Mes.MK050954_1705	-	-	-	-	-	-	-	-	0.18	-	0.17	-	-	-
Mes.MK052168_523	-	-	-	-	-	-	-	0.19	-	-	-	0.25	0.16	0.28
Mes.MK052813_4797	-	-	-	-	-	-	-	-	-	-	-	-	-	-
Mes.MK052813_4827	-	-	-	-	-	-	-	-	-	-	-	-	-	-
Mes.MK052813_4847	-	-	-	-	-	-	-	-	-	-	-	-	-	-
Mes.MK052813_4883	-	-	-	-	-	-	-	-	-	-	-	-	-	-
Mes.MK055684_1407	-	-	-	-	-	-	-	-	0.16	-	0.26	-	-	-
Mes.MK055684_1654	-	0.20	0.18	-	-	-	-	-	-	-	-	-	-	-
Mes.MK057448_1096	-	-	-	-	-	-	-	-	-	-	-	-	-	-
Mes.MK057448_1097	-	-	-	-	-	-	-	-	-	-	-	-	-	-
Mes.MK057448_1101	-	-	-	-	-	-	-	-	-	-	-	-	-	-
Mes.MK057448_1101	-	-	-	-	-	-	-	-	-	-	-	-	-	-
Mes.MK059215_1159	-	-	-	-	-	-	-	-	0.15	-	0.22	-	-	-
Mes.MK059215_1618	-	-	-	-	-	-	0.15	-	0.39	-	0.14	-	-	-
Mes.MK059215_488	-	-	-	-	-	-	0.19	0.17	-	-	0.24	-	-	-
Mes.MK059215_605	-	-	-	-	-	-	-	-	0.17	-	0.23	-	-	-
Mes.MK059215_837	-	-	-	-	-	-	-	-	0.15	-	0.15	-	-	-
Mes.MK059330_1759	-	-	-	-	-	-	-	-	0.15	-	0.16	-	-	-
Mes.MK059330_538	-	-	-	-	-	-	-	-	-	-	-	-	-	-
Mes.MK059330_862	0.15	0.19	-	-	-	-	0.21	-	-	-	0.21	-	-	-
Mes.MK059330_902	-	0.25	-	-	0.16	-	-	-	-	-	-	-	-	-
Mes.MK060192_101	-	-	-	-	-	-	-	-	-	-	0.17	-	-	-
Mes.MK060192_89	-	-	-	-	-	-	-	-	-	-	0.17	-	-	-
Mes.MK060872_100	-	-	-	-	-	-	-	-	0.17	-	0.18	-	-	-

Mes.MK060872_118	-	-	-	-	-	-	-	-	0.16	-	0.21	-	-	-
Mes.MK060872_130	-	-	-	-	-	-	-	-	0.15	-	0.21	-	-	-
Mes.MK060872_55	-	-	-	-	-	-	-	-	0.16	-	0.18	-	-	-
Mes.MK060872_58	-	-	-	-	-	-	-	-	0.16	-	0.18	-	-	-
Mes.MK060872_59	-	-	-	-	-	-	-	-	0.16	-	0.18	-	-	-
Mes.MK060872_60	-	-	-	-	-	-	-	-	0.16	-	0.18	-	-	-
Mes.MK060872_61	-	-	-	-	-	-	-	-	0.16	-	0.18	-	-	-
Mes.MK060872_64	-	-	-	-	-	-	-	-	0.16	-	0.18	-	-	-
Mes.MK060872_72	-	-	-	-	-	-	-	-	0.17	-	0.19	-	-	-
Mes.MK062247_1031	-	-	-	-	-	-	-	-	-	0.16	0.16	-	-	-
Mes.MK062247_1042	-	-	-	-	-	-	-	-	-	0.16	0.16	-	-	-
Mes.MK062793_2268	-	-	-	-	-	-	-	-	0.15	-	-	0.15	-	0.15
Mes.MK062793_2271	-	-	-	-	-	-	-	-	0.15	-	-	0.15	-	0.15

Table S9

Genes with repeatable cold-tolerance QTLs (reCT-QTLGs)

reCT-QTLGs	Gene Region	CDS region	Promoter region	CTIG	CTIF-1	CTIF-2	CTIF-3	CTIF-4	CTIF-5	RIG	RIF-1	RIF-2	LFIF-1	LFIF-2	LFIF-3	rSRY-1	rSRY-2
Mes.MK051000_11037	-	-	Mes.gk047745	3.1	0.1	0.5	0.2	5.8	7.1	1.2	0.0	0.7	3.0	3.0	1.8	2.1	4.2
Mes.MK002334_81504	Mes.gk013944	Mes.gk013944	-	0.9	0.2	0.5	4.7	0.8	3.9	0.0	3.0	2.7	2.9	4.7	2.8	1.8	0.0
Mes.MK034765_707	-	-	Mes.gk005622	4.6	0.9	0.0	1.5	5.4	0.0	0.3	2.8	0.0	3.7	2.8	0.2	1.3	2.7
Mes.MK038088_1107	-	-	Mes.gk030313	2.6	3.1	0.5	4.4	5.4	3.3	0.0	0.0	0.0	4.4	2.3	3.4	1.5	0.4
Mes.MK012026_1275	-	-	Mes.gk023872	0.0	1.2	0.2	4.1	0.6	0.8	0.8	9.0	4.7	4.2	2.4	0.1	0.0	3.1
Mes.MK003903_12523	-	-	Mes.gk043018	0.9	1.0	0.4	2.0	1.1	4.5	7.6	0.4	2.8	1.7	3.0	2.7	0.0	3.5
Mes.MK062339_2983	-	-	Mes.gk010478	0.0	4.1	0.3	0.1	1.1	3.7	1.1	0.3	3.2	0.0	0.0	0.7	6.0	3.8
Mes.MK012032_6186	Mes.gk002187	-	-	0.6	6.9	2.8	0.9	1.5	1.7	0.1	1.7	0.1	6.4	2.8	2.1	0.8	4.8
Mes.MK044967_2125	-	-	Mes.gk007244	0.4	3.1	4.0	3.5	1.3	1.2	6.3	2.8	1.9	0.0	0.6	0.0	0.2	0.1
Mes.MK003152_7293	-	-	Mes.gk014951	2.2	6.8	1.2	6.2	0.3	0.3	3.0	3.2	0.5	0.3	0.5	0.2	0.7	3.5
Mes.MK026462_4164	Mes.gk027414	Mes.gk027414	-	5.6	3.8	0.4	0.0	0.1	3.0	4.9	0.7	0.2	0.3	4.8	2.7	0.6	0.0
Mes.MK034495_19625	Mes.gk029453	-	-	5.5	0.1	3.5	3.3	0.0	3.0	0.7	0.0	1.9	1.4	0.9	3.3	1.3	0.4
Mes.MK002334_81502	Mes.gk013944	Mes.gk013944	-	0.3	0.7	0.0	4.7	0.8	3.9	0.1	0.5	2.7	2.9	4.7	2.8	1.8	0.0
Mes.MK005832_9678	Mes.gk018641	-	-	0.0	1.5	0.0	4.3	0.3	3.0	3.2	1.0	5.1	0.0	0.1	3.0	2.3	1.2
Mes.MK047755_3873	Mes.gk047520	Mes.gk047520	-	7.1	0.2	3.1	0.9	4.0	2.1	2.8	0.1	0.0	0.1	0.0	4.9	1.0	0.1
Mes.MK030461_3864	-	-	Mes.gk005020	7.3	2.3	0.4	2.7	3.4	3.6	0.0	0.1	0.0	1.1	2.6	2.7	0.6	0.5
Mes.MK047898_1053	Mes.gk032612	-	-	2.9	0.1	0.1	0.0	0.0	2.9	0.9	0.1	0.0	0.4	7.6	5.3	2.2	2.7
Mes.MK013971_4747	Mes.gk002516	Mes.gk002516	-	0.1	0.2	0.3	0.3	6.6	2.8	0.6	0.2	5.2	0.0	5.7	1.1	0.6	0.6
Mes.MK022839_3613	Mes.gk003864	Mes.gk003864	-	0.1	1.0	3.1	0.9	2.2	1.1	0.0	0.8	0.3	3.4	3.5	0.4	1.6	6.2
Mes.MK027470_9514	Mes.gk027644	Mes.gk027644	-	0.2	0.1	5.4	1.1	4.0	1.6	0.5	0.0	1.4	1.1	3.1	0.1	1.3	4.3
Mes.MK025563_4186	-	-	Mes.gk045937	2.7	3.0	0.8	0.1	1.9	3.4	0.1	0.0	0.2	1.3	4.8	2.2	0.0	5.7
Mes.MK004967_4042	Mes.gk043273	-	-	1.1	0.2	0.0	2.8	3.2	0.1	0.0	0.0	2.6	2.8	0.4	0.1	2.6	4.6
Mes.MK000106_50561	-	-	Mes.gk010835	0.3	3.1	0.8	0.1	0.7	1.2	0.2	1.1	0.2	0.1	4.4	1.0	3.0	4.3
Mes.MK020572_380	Mes.gk003516	Mes.gk003516	-	6.1	0.5	0.4	0.1	0.6	3.7	3.6	1.7	0.2	3.9	0.0	1.4	0.5	2.1
Mes.MK001362_28633	Mes.gk042154	-	-	0.8	1.4	6.4	2.6	3.1	0.5	1.0	1.1	0.4	8.1	1.6	3.1	2.3	0.2
Mes.MK017757_2117	-	-	Mes.gk003063	0.0	6.2	0.0	0.4	1.5	5.5	0.0	1.9	0.4	0.3	1.2	8.6	0.1	4.0
Mes.MK044501_8936	-	-	Mes.gk031749	1.2	0.6	6.2	3.0	3.1	1.0	0.1	0.9	1.6	0.2	0.7	0.4	1.6	6.4
Mes.MK041988_10548	Mes.gk047104	Mes.gk047104	-	0.6	0.0	4.1	2.2	6.5	0.2	3.7	0.0	0.0	1.8	1.2	0.1	0.0	2.9
Mes.MK054405_17289	-	-	Mes.gk048014	3.3	0.0	0.2	2.9	4.9	0.8	0.0	1.6	0.0	0.4	0.1	0.5	0.2	3.2
Mes.MK002743_3531	Mes.gk042655	-	-	0.5	4.4	0.0	4.0	1.1	2.8	0.1	2.1	1.9	0.0	4.5	0.0	0.1	0.0
Mes.MK002946_4586	Mes.gk014749	-	-	1.2	2.8	0.0	0.9	4.5	4.9	1.3	0.9	2.6	0.9	2.2	2.8	1.4	0.0
Mes.MK034694_1418	Mes.gk005605	Mes.gk005605	-	1.7	2.1	0.2	0.2	1.0	0.1	4.0	3.2	4.5	3.5	0.2	0.8	1.2	0.2
Mes.MK000794_13185	-	-	Mes.gk011752	3.8	2.0	0.8	0.3	3.0	0.3	0.5	0.0	0.0	4.0	0.0	0.2	3.9	0.4
Mes.MK004280_5968	Mes.gk016414	Mes.gk016414	-	0.1	0.1	0.7	1.0	0.1	3.9	0.9	0.6	0.0	0.6	3.0	5.0	3.8	2.0

Mes.MK007399_14007	Mes.gk021416	-	-	4.4	0.3	0.0	0.9	0.2	2.9	1.7	0.2	0.0	0.1	1.8	2.8	1.0	6.0
Mes.MK003518_9727	Mes.gk015419	Mes.gk015419	-	0.0	0.4	0.1	0.0	7.2	0.3	0.6	0.2	0.2	2.8	4.6	1.1	0.1	4.1
Mes.MK002686_17310	-	-	Mes.gk014406	2.2	1.1	0.7	0.2	0.3	3.7	0.1	0.2	0.8	3.7	0.0	4.2	2.9	0.8
Mes.MK049988_6104	-	-	Mes.gk047668	0.0	2.8	0.1	1.1	5.7	0.3	0.1	0.2	0.2	0.6	3.8	0.6	3.8	0.4
Mes.MK002317_62142	Mes.gk013867	-	-	4.2	0.8	3.6	4.3	0.9	0.0	0.0	0.1	1.3	0.6	0.1	0.1	1.5	3.5
Mes.MK046315_12193	Mes.gk032197	-	-	0.4	0.1	2.5	0.3	3.3	0.1	0.3	0.0	0.1	3.3	6.2	0.1	5.2	0.8
Mes.MK003544_10954	Mes.gk001036	Mes.gk001036	-	3.0	0.0	0.1	0.0	6.9	0.5	1.0	0.0	0.3	0.4	6.3	0.2	3.9	0.0
Mes.MK042247_2553	-	-	Mes.gk047128	0.1	3.7	5.0	0.2	1.7	1.3	1.6	0.2	0.2	0.0	0.6	3.4	3.4	0.0
Mes.MK030630_3597	-	-	Mes.gk028419	3.2	3.6	1.4	0.3	0.1	0.4	2.3	2.2	5.3	0.0	1.1	0.8	7.2	0.0
Mes.MK001465_24398	Mes.gk012710	Mes.gk012710	-	3.1	2.6	0.0	2.5	2.4	0.1	0.3	0.0	2.9	0.2	0.0	0.1	3.4	5.6
Mes.MK046094_1775	Mes.gk047398	-	-	0.1	0.0	0.6	6.9	0.5	1.2	0.1	3.5	0.0	5.0	1.2	4.1	0.1	0.2
Mes.MK028060_7498	Mes.gk027789	Mes.gk027789	-	6.4	2.2	0.2	1.2	0.6	1.7	0.1	3.8	3.0	0.4	1.4	4.3	1.2	0.0
Mes.MK010174_16163	Mes.gk023347	-	-	0.1	5.8	0.0	0.7	0.2	4.3	0.1	0.4	0.2	0.6	6.9	4.3	0.2	0.1
Mes.MK017757_2004	-	-	Mes.gk003063	0.0	5.2	1.7	0.3	3.8	2.0	0.1	3.7	0.1	0.1	0.4	3.6	1.2	1.0
Mes.MK031712_7339	Mes.gk028817	-	-	3.0	4.7	4.3	0.2	0.4	0.5	0.9	0.1	0.0	3.3	2.4	0.1	0.0	1.3
Mes.MK022961_2412	-	-	Mes.gk003902	0.5	5.0	0.6	0.3	8.0	0.4	0.9	6.0	0.2	0.1	4.3	2.1	0.1	1.8
Mes.MK029828_509	-	-	Mes.gk046231	4.3	3.4	1.2	3.5	3.6	0.4	0.3	2.2	1.6	0.3	0.0	2.0	1.6	0.3
Mes.MK023200_5023	Mes.gk026631	-	-	4.5	0.7	3.2	2.1	0.2	5.8	0.1	2.6	1.5	0.1	0.0	1.7	1.1	5.8
Mes.MK000887_519	-	-	Mes.gk000275	1.6	2.1	0.3	2.9	3.0	1.4	0.0	1.9	1.5	3.2	1.0	0.4	3.8	0.1
Mes.MK051445_6526	-	-	Mes.gk008299	0.0	4.0	2.2	0.5	0.0	6.9	0.1	4.3	0.0	0.7	0.8	1.6	7.1	0.1
Mes.MK036083_14398	Mes.gk005832	Mes.gk005832	-	2.9	0.2	0.3	0.4	3.9	0.0	1.9	2.9	0.4	0.2	2.1	0.0	1.5	2.9
Mes.MK018779_10068	Mes.gk025472	-	-	4.4	0.0	3.6	0.1	0.2	0.0	3.0	1.8	1.3	0.2	0.2	0.0	2.9	0.3
Mes.MK059674_3764	-	-	Mes.gk009887	0.1	0.1	0.5	0.2	0.0	4.9	0.2	0.1	0.5	0.0	3.9	3.7	3.1	1.0
Mes.MK042247_2531	-	-	Mes.gk047128	0.0	5.1	5.0	0.0	1.1	0.4	1.1	0.2	0.2	0.2	0.1	2.8	3.1	0.2
Mes.MK054615_2771	Mes.gk008741	-	-	0.0	1.3	0.2	1.3	0.1	5.9	1.8	3.1	2.9	0.8	2.3	2.0	0.0	5.4
Mes.MK032048_14042	-	-	Mes.gk028894	0.0	0.1	1.4	1.0	0.7	0.1	0.1	0.4	0.0	3.3	5.2	3.2	1.3	4.0
Mes.MK004067_20869	Mes.gk016130	-	-	1.1	3.8	3.3	0.3	0.4	0.1	0.3	1.9	2.2	2.3	0.5	0.7	3.5	3.4
Mes.MK003818_604	Mes.gk042990	-	-	0.0	9.4	0.3	3.0	0.0	2.6	1.2	0.0	0.2	0.0	0.3	0.7	3.3	3.3
Mes.MK012026_1201	-	-	Mes.gk023872	1.7	2.7	0.8	0.3	2.4	6.3	0.5	3.3	0.0	0.8	0.2	3.9	0.2	0.2
Mes.MK000106_57336	-	-	Mes.gk010836	3.7	0.4	0.0	0.7	1.0	2.0	5.0	0.2	0.0	5.9	3.4	0.7	1.3	0.0
Mes.MK031530_44451	-	-	Mes.gk028764	3.6	0.2	0.2	0.2	0.0	0.6	4.5	3.2	3.5	1.5	1.4	1.1	2.0	0.2
Mes.MK036279_5015	Mes.gk046673	Mes.gk046673	-	0.1	0.3	2.8	0.7	0.7	0.0	0.0	0.0	3.3	3.3	5.7	0.2	1.1	0.0
Mes.MK057983_13513	Mes.gk049108	-	-	1.1	6.8	0.3	0.1	1.1	8.0	0.1	0.0	0.5	2.7	0.0	5.1	1.0	0.0
Mes.MK013621_5542	-	-	Mes.gk045011	0.0	0.6	1.4	3.1	0.0	2.8	0.4	0.9	0.1	3.2	0.7	3.4	0.0	1.0
Mes.MK003856_3326	Mes.gk015887	-	-	0.5	3.8	4.1	0.5	1.4	1.7	0.0	5.1	1.5	1.1	4.4	1.2	0.1	0.2
Mes.MK059203_3555	Mes.gk038174	Mes.gk038174	-	0.1	4.0	3.2	0.0	0.9	3.3	3.1	0.1	0.3	0.5	0.7	2.0	1.4	1.3
Mes.MK027755_1789	-	-	Mes.gk027706	3.4	0.0	3.4	0.0	5.4	1.9	0.0	0.0	0.2	5.2	0.0	2.7	0.5	0.3
Mes.MK003152_7329	-	-	Mes.gk014951	2.4	6.1	2.0	7.7	0.1	0.4	4.3	3.9	0.3	0.8	0.5	0.5	0.3	2.3
Mes.MK021196_5498	Mes.gk026087	-	-	2.8	0.4	0.3	3.2	0.0	2.4	0.4	2.0	0.7	1.8	0.2	3.7	1.9	3.0
Mes.MK046523_1112	Mes.gk032257	Mes.gk032257	-	0.3	0.2	0.2	3.6	1.6	5.5	0.6	0.2	0.0	1.4	2.3	6.8	0.0	3.3
Mes.MK025703_19408	Mes.gk027212	-	-	3.1	0.0	1.7	0.5	0.0	0.1	1.5	0.0	0.6	3.4	2.8	1.1	3.0	0.0
Mes.MK035348_206	Mes.gk005716	Mes.gk005716	-	0.1	1.4	2.9	2.0	0.4	0.0	3.2	3.0	0.8	0.3	0.2	1.3	0.1	2.9
Mes.MK035348_204	Mes.gk005716	Mes.gk005716	-	0.5	1.3	2.9	2.2	0.4	0.1	3.2	3.0	0.9	0.0	0.2	1.6	0.4	3.2
Mes.MK001371_31643	Mes.gk042158	-	-	0.0	0.0	2.2	0.5	3.3	2.7	0.2	0.2	1.5	3.3	0.0	2.9	0.4	0.0
Mes.MK002213_492	-	-	Mes.gk042442	2.7	3.5	0.1	0.8	1.9	0.2	0.2	0.1	0.0	0.0	6.1	2.9	1.4	1.1
Mes.MK012060_31292	Mes.gk023885	-	-	1.3	3.6	1.7	3.0	0.6	2.2	0.0	4.6	1.6	7.3	0.1	0.4	0.1	0.1
Mes.MK060744_7140	Mes.gk010113	Mes.gk010113	-	0.6	4.0	0.9	0.5	0.8	0.1	2.8	0.4	0.0	3.9	0.1	5.1	0.1	0.2
Mes.MK024522_5706	Mes.gk045854	-	-	4.6	0.0	2.8	1.0	0.8	2.9	0.8	2.6	1.5	1.3	0.1	5.3	0.1	0.8
Mes.MK035076_2575	Mes.gk005676	-	-	0.3	5.7	0.4	4.9	2.8	0.6	0.3	0.9	0.0	0.1	1.4	3.6	1.4	0.0
Mes.MK047677_5411	Mes.gk032556	-	-	0.1	3.3	1.7	1.0	3.6	0.1	0.0	0.9	0.1	8.6	6.4	0.2	0.1	0.0
Mes.MK003263_5633	Mes.gk015116	Mes.gk015116	-	0.0	0.5	0.3	0.0	0.5	3.5	3.5	1.2	0.8	0.0	3.5	5.8	0.7	0.0
Mes.MK003263_5631	Mes.gk015116	Mes.gk015116	-	0.0	0.4	0.3	0.0	0.4	3.2	3.7	1.2	0.9	0.0	3.8	5.8	0.7	0.1
Mes.MK044107_6854	Mes.gk007103	-	-	0.2	0.1	4.0	3.0	0.2	0.0	0.1	1.5	0.0	2.8	3.4	0.0	0.1	1.2
Mes.MK002388_47027	-	-	Mes.gk014037	3.6	0.4	2.9	3.9	0.2	0.0	0.0	1.2	0.2	0.8	0.4	4.3	0.2	0.3
Mes.MK051204_1067	Mes.gk033369	Mes.gk033369	-	2.8	1.1	0.5	0.3	0.4	1.7	4.7	0.4	0.4	3.8	0.1	4.7	0.0	0.2
Mes.MK000341_17669	-	-	Mes.gk000115	13	2.8	1.2	0.0	1.2	0.1	0.1	3.5	0.1	0.3	2.9	0.4	0.1	0.2
Mes.MK000341_17691	-	-	Mes.gk000115	12.	3.7	1.2	0.3	0.6	0.1	0.0	5.3	0.1	0.4	3.6	0.3	0.2	0.0
Mes.MK058985_37493	-	-	Mes.gk009710	5.4	3.3	6.6	1.4	0.1	1.8	0.0	1.7	0.4	0.0	0.0	3.0	0.0	0.1
Mes.MK058985_37495	-	-	Mes.gk009710	5.5	3.5	6.6	1.4	0.1	1.8	0.0	1.5	0.4	0.0	0.0	3.0	0.0	0.1
Mes.MK001377_8160	Mes.gk012502	-	-	0.3	1.5	0.0	1.1	0.8	1.7	0.7	3.5	10.	0.8	0.3	0.1	3.9	0.5
Mes.MK010955_156	-	-	Mes.gk001969	3.9	2.7	1.6	0.2	0.0	2.7	0.5	1.0	0.6	2.3	0.1	0.1	0.0	5.9

Mes.MK040449_998	Mes.gk030811	Mes.gk030811	-	0.1	7.8	4.3	0.4	0.5	3.3	0.1	0.0	0.8	2.2	1.1	0.6	0.0	1.1
Mes.MK025967_4841	-	-	Mes.gk045965	0.7	0.0	2.4	0.9	4.5	0.7	1.1	0.2	0.2	5.4	4.0	0.2	0.0	1.2
Mes.MK049198_13143	Mes.gk032950	-	-	1.9	0.1	0.4	0.1	1.8	0.4	0.0	3.0	0.0	2.7	3.1	0.2	1.7	0.5
Mes.MK055035_5763	Mes.gk008822	Mes.gk008822	-	2.9	0.0	4.4	1.9	0.0	0.6	0.0	0.3	0.1	0.0	1.5	2.7	1.1	4.5
Mes.MK055035_5739	Mes.gk008822	Mes.gk008822	-	4.0	0.4	3.4	1.7	0.1	0.7	0.0	0.5	0.0	0.0	1.0	2.2	0.8	5.3
Mes.MK002152_432	Mes.gk013520	-	-	0.1	2.7	1.6	0.7	1.1	2.9	1.3	0.8	0.4	5.6	0.6	0.3	0.5	0.4
Mes.MK000971_768	Mes.gk011964	Mes.gk011964	-	1.4	0.8	0.4	0.3	0.4	4.0	6.2	11.	0.2	0.1	0.0	1.9	0.0	1.9
Mes.MK042131_2570	Mes.gk031189	-	-	0.2	5.4	0.6	0.0	0.8	0.2	1.1	0.6	2.5	3.7	2.9	0.1	0.5	0.3
Mes.MK002795_14976	-	-	Mes.gk014569	3.8	0.1	3.2	0.0	1.3	0.0	0.1	0.1	2.9	0.7	0.3	0.5	2.3	0.3
Mes.MK004129_34017	Mes.gk016223	Mes.gk016223	-	2.1	6.2	0.0	0.4	0.0	3.3	0.3	0.0	0.2	3.5	1.6	0.9	0.0	0.4
Mes.MK002294_27632	Mes.gk013716	Mes.gk013716	-	0.0	0.5	0.5	0.9	1.0	1.8	1.5	2.8	3.3	0.3	1.2	0.7	3.6	0.5
Mes.MK019169_21342	Mes.gk025573	-	-	2.4	1.6	1.5	1.0	0.1	0.7	4.7	3.0	0.2	0.0	0.1	1.2	0.3	3.6
Mes.MK003698_12381	-	-	Mes.gk001091	0.1	0.0	0.1	0.1	0.0	0.1	0.3	1.2	2.7	0.0	0.0	0.5	4.1	3.2
Mes.MK003698_12400	-	-	Mes.gk001091	0.1	0.0	0.1	0.1	0.0	0.1	0.3	1.2	2.7	0.0	0.0	0.5	4.1	3.2
Mes.MK002836_30416	-	-	Mes.gk014606	0.4	4.0	1.6	0.4	1.8	0.0	0.2	3.4	5.1	0.3	0.3	0.3	1.7	2.2
Mes.MK033753_7825	-	-	Mes.gk029282	1.2	0.2	2.0	0.4	0.5	3.0	2.8	2.8	0.5	0.7	0.1	0.7	0.1	2.4
Mes.MK052354_4582	Mes.gk008414	Mes.gk008414	-	1.3	0.1	0.0	4.1	0.3	4.1	0.0	0.0	4.0	1.3	0.5	0.1	0.2	0.3
Mes.MK059203_3619	Mes.gk038174	Mes.gk038174	-	3.1	3.8	0.7	0.0	1.9	0.3	1.3	0.0	1.2	0.1	3.3	0.0	0.0	1.0
Mes.MK030449_9572	-	-	Mes.gk028378	1.3	4.3	0.0	4.7	0.0	4.8	0.5	0.1	1.0	0.2	0.5	1.9	1.8	0.9
Mes.MK003171_2615	Mes.gk000911	-	-	3.5	0.3	3.5	0.5	3.3	0.9	0.1	0.1	1.1	0.1	0.4	0.0	0.0	0.1
Mes.MK001302_6462	-	-	Mes.gk012377	0.5	3.4	0.1	0.4	4.5	0.1	0.1	1.1	0.1	0.1	5.2	0.1	0.7	0.0
Mes.MK019194_8181	Mes.gk025580	Mes.gk025580	-	2.4	3.2	0.9	0.2	4.3	1.1	0.1	2.6	0.8	0.0	0.7	1.1	3.2	0.5
Mes.MK019194_8183	Mes.gk025580	Mes.gk025580	-	2.4	3.2	0.9	0.2	4.3	1.1	0.1	2.6	0.8	0.0	0.7	1.1	3.2	0.5
Mes.MK000339_15862	Mes.gk011091	Mes.gk011091	-	0.0	6.0	0.7	0.1	0.8	2.9	0.0	0.1	0.9	0.6	1.9	0.1	4.5	1.2
Mes.MK049202_9179	Mes.gk032952	-	-	2.9	0.1	0.6	0.8	7.2	0.6	0.3	0.6	1.7	0.0	0.4	1.0	1.4	5.8
Mes.MK003942_4842	Mes.gk043026	-	-	2.9	0.0	0.0	0.8	4.8	0.0	2.3	1.0	1.1	0.1	1.1	0.0	2.8	0.1
Mes.MK046371_5013	Mes.gk032219	Mes.gk032219	-	0.0	0.0	0.1	0.0	0.8	2.5	0.1	0.9	0.5	1.5	3.3	6.1	0.0	3.7
Mes.MK022281_6965	Mes.gk026397	Mes.gk026397	-	2.2	0.9	2.0	0.0	0.0	0.0	0.0	0.0	0.0	2.8	4.2	0.5	4.6	0.8
Mes.MK030418_6677	Mes.gk046290	-	-	3.3	0.8	4.2	0.0	0.2	0.1	0.1	0.1	0.5	0.0	0.0	0.2	5.6	0.6
Mes.MK001728_1566	-	-	Mes.gk000497	0.4	2.1	0.0	0.4	0.1	1.3	0.8	0.4	0.0	4.5	0.4	1.3	5.6	4.9
Mes.MK020662_5998	Mes.gk045550	Mes.gk045550	-	8.3	0.5	0.4	0.2	0.0	0.0	0.0	3.4	6.6	0.5	1.4	0.0	0.2	0.2
Mes.MK020662_6007	Mes.gk045550	Mes.gk045550	-	9.6	0.2	0.4	0.6	0.1	0.2	0.0	3.5	5.5	1.5	2.2	0.3	0.2	0.2
Mes.MK005066_10104	Mes.gk043302	-	-	0.5	0.4	0.4	0.1	11.	6.6	1.7	1.2	0.2	0.2	0.9	3.7	1.4	1.7
Mes.MK013698_8548	Mes.gk024242	-	-	0.1	0.0	0.5	0.4	0.3	3.3	0.1	0.1	1.2	6.6	0.2	5.5	0.8	0.4
Mes.MK000126_13538	Mes.gk010874	-	-	0.2	1.2	0.4	3.0	0.7	3.2	0.1	0.0	0.8	0.2	2.1	4.3	0.2	0.5
Mes.MK001089_1794	-	-	Mes.gk012111	4.1	1.8	3.8	0.1	3.5	0.8	0.5	1.7	0.2	0.1	1.8	1.6	0.2	0.2
Mes.MK014140_2157	Mes.gk024381	-	-	2.7	0.9	0.6	3.5	0.2	4.9	0.1	0.1	0.1	1.7	0.4	7.2	0.9	0.0
Mes.MK045540_1967	-	-	Mes.gk032016	4.5	0.0	2.0	0.5	0.2	6.4	0.1	0.2	0.0	0.0	0.1	3.7	2.1	0.0
Mes.MK004454_1272	Mes.gk016647	-	-	0.8	2.2	0.7	1.6	0.4	1.6	0.8	0.2	0.8	2.8	3.5	5.1	0.0	1.1
Mes.MK001112_28150	Mes.gk042083	-	-	2.2	4.0	0.1	3.2	0.7	0.4	0.0	0.0	0.0	2.7	1.3	0.0	0.2	0.0
Mes.MK056035_16192	Mes.gk035170	Mes.gk035170	-	0.6	0.0	1.6	0.7	0.8	0.5	0.0	3.6	1.1	9.1	4.1	0.7	1.8	0.0
Mes.MK025967_4838	-	-	Mes.gk045965	0.1	0.8	3.3	0.0	5.3	0.3	0.0	0.6	0.1	4.1	2.0	0.0	0.5	0.1
Mes.MK021863_740	-	-	Mes.gk003699	0.0	4.3	3.8	0.1	3.6	0.8	0.3	0.0	0.0	0.3	0.8	0.0	0.5	0.5
Mes.MK022859_13121	Mes.gk003870	-	-	2.7	0.1	0.1	0.1	0.9	0.2	0.6	0.2	0.2	1.8	3.7	2.9	2.4	0.5
Mes.MK002722_25251	-	-	Mes.gk014496	1.0	3.6	1.1	2.4	0.3	3.0	1.0	1.2	0.2	1.5	0.9	3.9	1.0	1.1
Mes.MK002297_31795	Mes.gk013729	-	-	0.7	10.	0.1	2.2	0.1	4.7	4.1	1.7	0.2	0.7	0.0	1.6	1.0	0.0
Mes.MK001841_7781	Mes.gk013142	Mes.gk013142	-	5.9	0.0	1.6	0.2	3.2	0.0	0.1	1.0	1.6	0.1	1.6	0.2	3.1	1.3
Mes.MK042282_1129	Mes.gk006822	-	-	0.1	0.2	0.1	0.1	1.6	0.0	0.4	0.9	2.0	5.0	9.0	0.8	5.2	0.3
Mes.MK034732_1547	Mes.gk029530	-	-	4.5	0.9	0.8	0.1	2.7	4.1	0.1	0.7	2.4	0.0	0.4	1.1	2.7	0.6
Mes.MK022743_1294	Mes.gk003848	-	-	0.5	1.5	0.8	0.1	0.1	1.5	5.4	3.7	0.6	0.0	0.1	1.3	1.7	2.7
Mes.MK011929_8323	Mes.gk023838	Mes.gk023838	-	0.1	4.9	3.3	0.1	0.0	0.1	0.2	0.1	0.7	0.4	0.1	0.2	0.1	4.0
Mes.MK030875_6213	Mes.gk046332	-	-	0.0	1.0	0.2	0.2	1.1	0.0	1.1	0.1	1.9	1.2	5.1	2.9	1.6	4.1
Mes.MK000872_5134	Mes.gk000266	-	-	3.8	3.2	1.3	0.8	0.1	0.2	0.0	0.3	0.0	0.3	2.1	1.7	2.8	0.1
Mes.MK020085_10906	Mes.gk025847	-	-	2.6	0.4	0.8	1.4	2.9	3.7	2.1	0.4	1.1	0.5	0.0	0.2	5.2	2.4
Mes.MK030449_9439	-	-	Mes.gk028378	0.9	4.4	0.0	6.1	0.2	0.4	0.6	0.0	0.9	0.9	0.0	2.4	3.1	0.1
Mes.MK014606_6452	-	-	Mes.gk045112	0.0	0.4	0.1	10.	5.4	1.7	1.6	0.1	0.0	1.8	1.6	0.2	4.2	0.9
Mes.MK010955_112	-	-	Mes.gk001969	5.6	2.6	0.9	0.1	0.8	3.1	0.5	0.3	0.9	1.4	0.0	0.1	0.2	3.6
Mes.MK001395_20033	Mes.gk012598	Mes.gk012598	-	0.1	0.0	1.5	1.0	1.2	13	1.1	0.1	0.1	0.2	3.0	8.1	0.3	2.2
Mes.MK042341_16474	-	-	Mes.gk031243	0.3	7.3	0.0	0.3	0.8	4.3	1.1	3.8	0.2	0.1	0.0	0.0	2.2	0.6
Mes.MK012655_4567	Mes.gk002289	-	-	0.9	3.2	3.7	0.5	6.5	0.1	0.1	1.0	0.1	2.1	1.5	1.4	0.1	0.0
Mes.MK000492_26815	-	-	Mes.gk011318	1.8	0.0	0.2	1.3	2.4	0.4	3.1	0.7	4.9	1.0	7.7	0.2	2.2	0.0

Mes.MK053494_3387	Mes.gk008592	-	-	1.7	3.9	3.4	0.5	0.5	0.0	3.7	0.0	1.3	2.3	0.8	0.1	1.5	0.6
Mes.MK026202_4523	Mes.gk027339	-	-	3.3	1.4	2.9	0.0	0.4	1.0	1.4	3.2	1.5	0.1	0.3	0.4	0.1	1.6
Mes.MK060122_6620	Mes.gk009986	-	-	2.3	0.4	6.2	2.8	0.0	0.2	0.8	0.0	0.3	0.0	0.3	5.7	1.6	2.7
Mes.MK035744_5642	Mes.gk029742	-	-	1.7	5.3	0.1	2.0	2.1	1.0	0.1	2.0	0.1	4.5	2.0	3.4	0.2	0.0
Mes.MK014642_9158	Mes.gk045117	-	-	1.7	1.9	0.8	0.3	6.7	3.4	0.1	0.2	0.3	5.0	2.7	0.3	0.8	0.3
Mes.MK026108_8848	-	-	Mes.gk027315	0.0	0.3	3.3	0.2	0.0	0.2	0.9	0.7	2.2	5.1	0.6	3.9	0.4	0.9
Mes.MK049565_5681	-	-	Mes.gk047649	6.5	0.1	0.0	0.4	2.0	0.5	0.0	1.3	0.0	0.0	6.2	6.1	1.1	0.6
Mes.MK051623_13694	Mes.gk047796	-	-	0.0	5.1	0.1	0.3	4.9	0.0	0.1	0.4	1.0	3.1	1.2	0.0	1.7	2.5
Mes.MK003942_4844	Mes.gk043026	-	-	2.8	0.3	0.0	0.7	10.	0.4	0.4	0.3	2.1	0.8	3.7	0.2	0.1	0.3
Mes.MK056155_3294	-	-	Mes.gk009058	4.4	1.6	0.5	0.5	2.1	3.0	3.6	0.1	0.0	0.0	1.3	1.0	0.7	0.6
Mes.MK024522_5513	Mes.gk045854	-	-	0.1	2.7	0.8	0.1	0.0	3.9	2.4	1.9	0.7	0.2	0.4	4.4	0.5	0.7
Mes.MK000683_8048	Mes.gk011576	-	-	4.2	1.6	5.0	1.4	2.8	1.6	0.4	0.5	1.0	0.0	0.1	1.2	0.5	2.5
Mes.MK009951_13683	-	-	Mes.gk001837	0.5	0.6	0.3	5.2	3.3	0.1	2.0	0.1	0.0	3.2	0.6	0.3	1.9	0.0
Mes.MK012077_7002	Mes.gk023889	Mes.gk023889	-	0.1	0.5	0.4	0.0	4.6	3.8	0.5	0.0	0.0	0.2	3.4	1.5	2.6	0.0
Mes.MK004015_5469	Mes.gk016087	Mes.gk016087	-	0.1	6.5	0.1	0.0	3.8	0.2	0.0	4.8	1.1	0.9	1.5	0.0	1.0	0.0
Mes.MK030138_2805	Mes.gk046265	Mes.gk046265	-	3.5	4.2	0.2	1.0	0.1	0.0	0.0	1.9	0.7	0.0	2.2	3.1	0.0	0.0
Mes.MK001812_71546	Mes.gk013075	Mes.gk013075	-	1.0	1.0	6.7	0.0	4.2	0.6	1.2	0.5	0.1	0.0	3.1	0.9	0.0	0.2
Mes.MK030679_575	Mes.gk005059	-	-	0.5	6.1	0.2	0.5	0.2	1.8	5.0	0.9	5.3	0.6	1.6	0.2	0.8	0.0
Mes.MK007856_2245	Mes.gk022233	Mes.gk022233	-	1.0	0.1	0.2	0.9	4.3	1.9	1.8	0.3	0.1	0.0	5.8	4.5	0.3	2.1
Mes.MK020716_15963	Mes.gk025989	-	-	0.4	2.9	2.7	0.6	0.3	0.5	0.5	0.5	1.7	6.9	0.1	0.0	0.3	0.0
Mes.MK027763_1750	-	-	Mes.gk004624	4.0	3.1	0.5	6.6	2.1	0.1	0.8	0.3	1.2	0.0	1.6	1.8	0.1	0.2
Mes.MK003152_7374	-	-	Mes.gk014951	0.6	8.2	1.2	5.2	0.3	0.0	1.8	2.9	0.6	0.2	0.0	0.4	0.9	1.3
Mes.MK003152_7243	-	-	Mes.gk014951	1.5	5.6	2.3	3.7	0.8	0.3	0.9	3.5	0.7	0.1	0.3	0.7	0.4	0.6
Mes.MK043700_7876	-	-	Mes.gk031541	1.5	1.3	1.9	0.2	0.1	0.3	0.7	0.1	0.1	4.1	3.3	0.1	3.6	0.0
Mes.MK053529_6706	Mes.gk047951	-	-	3.0	1.8	0.0	0.5	2.4	6.5	0.0	1.5	0.0	0.7	1.4	1.5	4.1	0.0
Mes.MK052685_4456	Mes.gk047873	-	-	1.3	0.0	0.0	1.5	1.5	1.6	2.6	0.7	1.3	3.7	2.9	0.0	1.3	2.7
Mes.MK046610_8904	-	-	Mes.gk032293	2.9	0.6	0.3	2.0	2.9	1.1	0.5	0.2	0.1	0.2	1.1	1.0	2.8	0.5
Mes.MK046610_8931	-	-	Mes.gk032293	2.9	0.6	0.3	2.2	3.1	1.1	0.5	0.2	0.2	0.3	1.4	1.3	3.0	0.5
Mes.MK059676_7802	Mes.gk038577	Mes.gk038577	-	0.2	1.5	0.0	0.3	1.8	0.1	0.1	1.3	0.1	3.8	4.3	2.4	3.1	0.3
Mes.MK005675_3858	Mes.gk018327	-	-	3.6	0.0	7.5	0.7	1.3	1.4	0.0	0.4	0.4	2.3	0.0	0.5	1.0	3.9
Mes.MK005675_3838	Mes.gk018327	-	-	2.8	0.0	7.3	0.6	1.9	0.6	0.2	0.5	0.1	2.1	0.1	0.2	0.6	3.2
Mes.MK005675_3861	Mes.gk018327	-	-	3.9	0.0	7.7	0.6	1.2	1.2	0.0	0.2	0.4	2.1	0.0	0.3	1.2	4.2
Mes.MK011471_1142	Mes.gk023679	Mes.gk023679	-	0.4	1.4	0.2	8.8	3.6	0.0	0.3	0.8	0.0	2.4	1.5	0.2	2.9	0.1
Mes.MK002677_6985	Mes.gk014396	Mes.gk014396	-	0.5	0.0	4.3	0.0	0.8	0.5	4.2	1.2	4.4	0.9	2.7	0.2	0.8	1.3
Mes.MK037048_5700	Mes.gk030047	Mes.gk030047	-	0.1	3.0	1.0	1.0	0.0	1.7	0.6	5.6	3.7	0.3	0.0	0.8	0.0	0.1
Mes.MK003398_6310	Mes.gk015246	Mes.gk015246	-	0.1	0.3	0.0	0.0	0.0	2.7	0.4	4.6	0.7	2.7	1.4	6.8	0.1	0.0
Mes.MK002905_7815	Mes.gk000835	-	-	0.1	1.6	0.9	2.8	0.4	3.4	0.2	1.4	3.0	0.5	1.6	2.1	0.3	1.0
Mes.MK028060_7498	Mes.gk027789	Mes.gk027789	-	0.3	0.4	0.2	0.2	0.4	1.7	3.6	0.6	2.8	0.5	0.3	4.7	0.8	0.0
Mes.MK025441_8586	-	-	Mes.gk027136	5.2	0.5	1.5	1.7	2.8	1.6	5.8	0.1	0.1	1.3	0.1	2.2	0.1	0.5
Mes.MK026557_5001	-	-	Mes.gk027447	0.1	3.4	2.4	1.2	2.3	3.3	0.4	0.9	0.0	4.4	1.1	0.1	0.0	0.0
Mes.MK052830_840	Mes.gk008502	-	-	5.0	3.1	1.2	0.0	0.9	1.2	0.6	1.8	0.3	0.3	3.7	1.5	1.5	0.2
Mes.MK009582_1316	Mes.gk001768	Mes.gk001768	-	3.5	0.4	1.1	2.7	5.2	0.0	0.3	0.1	0.0	0.4	2.3	0.1	0.3	0.2
Mes.MK001289_1397	-	-	Mes.gk000381	1.4	1.5	0.0	1.0	0.1	1.5	3.9	3.1	0.8	0.0	1.3	5.7	2.2	1.2
Mes.MK001289_1420	-	-	Mes.gk000381	0.8	2.3	0.0	0.3	0.1	0.9	4.1	2.8	0.6	0.0	1.7	4.1	1.2	0.7
Mes.MK026108_8763	-	-	Mes.gk027315	3.7	0.0	4.5	0.1	1.0	1.5	0.6	0.8	0.2	2.3	0.3	6.4	0.5	0.2
Mes.MK018151_2170	Mes.gk003116	Mes.gk003116	-	1.9	1.6	2.0	1.6	2.9	0.5	4.6	3.4	2.3	0.7	0.4	0.0	0.0	0.4
Mes.MK018151_2168	Mes.gk003116	Mes.gk003116	-	4.6	3.6	0.1	0.2	1.3	1.3	1.7	1.7	2.0	1.6	3.9	0.1	0.1	0.0
Mes.MK003340_5780	Mes.gk015194	-	-	0.5	0.9	0.0	0.6	0.1	0.6	0.6	3.0	0.2	3.4	4.4	0.3	0.0	0.4
Mes.MK002079_7790	Mes.gk000587	Mes.gk000587	-	0.3	1.9	0.1	1.8	3.7	0.3	2.5	2.9	3.8	0.1	0.3	1.4	0.1	0.8
Mes.MK013999_320	Mes.gk002520	-	-	0.1	1.9	0.4	6.9	0.4	1.1	3.3	0.6	4.7	1.4	0.0	2.2	0.0	0.0
Mes.MK001809_11631	Mes.gk013061	-	-	0.4	0.0	0.5	1.0	0.1	3.3	0.7	2.7	2.0	6.3	4.4	1.4	0.3	0.8
Mes.MK005675_3862	Mes.gk018327	-	-	2.8	0.1	3.3	0.2	1.0	0.1	0.0	0.5	0.8	3.8	0.2	0.1	0.7	0.2
Mes.MK004517_8036	-	-	Mes.gk001334	3.8	0.1	0.1	0.2	3.0	3.0	1.9	1.1	0.6	1.2	2.4	2.2	0.3	0.0
Mes.MK054449_1132	Mes.gk008711	-	-	0.0	3.1	1.3	0.0	5.2	0.0	2.0	0.1	1.9	0.3	4.5	0.2	0.0	0.0
Mes.MK034712_1715	Mes.gk029516	-	-	1.3	5.1	1.2	0.3	0.6	5.5	0.1	0.3	0.1	0.3	0.6	9.2	0.2	2.5
Mes.MK048244_3354	Mes.gk007828	Mes.gk007828	-	1.6	0.2	0.1	0.4	3.1	0.7	0.8	0.3	0.0	0.1	3.7	3.5	0.9	0.0
Mes.MK048084_4795	Mes.gk032676	-	-	1.2	3.5	0.0	0.9	1.3	1.3	0.2	0.4	2.4	0.2	4.7	3.9	0.2	0.3
Mes.MK027755_1836	-	-	Mes.gk027706	1.7	0.0	2.6	0.0	6.2	2.5	0.2	0.1	0.0	7.2	1.2	2.8	0.2	0.1
Mes.MK004056_7407	Mes.gk016117	Mes.gk016117	-	0.1	3.1	1.5	0.0	4.4	0.3	0.0	1.8	1.9	0.2	3.4	0.1	0.1	0.1
Mes.MK046367_276	Mes.gk047418	-	-	5.9	1.8	3.2	0.2	2.9	0.0	0.7	1.4	0.1	0.0	0.2	0.3	1.2	2.1
Mes.MK003870_6529	-	-	Mes.gk015917	8.1	0.6	0.0	4.3	0.1	0.1	0.7	3.9	1.1	0.2	0.5	0.2	0.0	0.0

Mes.MK004129_33958	-	-	Mes.gk016223	1.8	4.4	0.3	0.2	0.0	3.5	0.3	0.3	0.4	2.4	1.1	0.3	0.0	0.9
Mes.MK000981_10327	Mes.gk011974	-	-	0.0	0.0	1.3	0.3	0.3	0.0	0.6	0.9	0.1	0.1	0.2	0.4	3.3	9.7
Mes.MK000186_3042	Mes.gk010930	Mes.gk010930	-	0.4	3.0	0.0	0.2	3.4	1.3	0.0	0.8	2.2	2.0	0.5	0.2	0.0	0.7
Mes.MK041439_3250	Mes.gk031017	Mes.gk031017	-	0.2	0.0	0.0	0.2	2.5	0.2	4.4	4.3	0.1	2.4	1.0	1.7	2.5	0.3
Mes.MK006776_19276	Mes.gk020247	-	-	0.8	0.0	0.7	0.4	1.0	0.0	0.5	1.0	2.2	0.2	0.0	0.0	9.8	3.7
Mes.MK011913_949	Mes.gk002166	Mes.gk002166	-	0.5	4.6	0.1	0.4	5.6	2.1	1.6	0.1	0.0	0.0	1.7	0.3	0.2	0.6
Mes.MK009451_205	-	-	Mes.gk001744	1.2	0.1	0.0	0.5	0.0	1.1	0.3	0.5	1.4	1.3	2.2	0.2	4.6	2.9
Mes.MK003263_5497	-	-	Mes.gk015116	1.2	1.0	0.6	1.6	2.4	0.0	0.2	1.4	0.8	0.8	0.3	0.7	4.8	2.9
Mes.MK027638_4387	Mes.gk004598	-	-	0.0	0.2	0.0	0.2	0.5	0.8	0.4	0.0	0.2	1.8	0.7	0.1	3.8	3.0
Mes.MK002022_55785	Mes.gk013326	Mes.gk013326	-	0.4	3.1	0.0	0.5	5.9	0.1	1.4	0.2	0.6	0.6	1.7	0.5	0.9	0.0
Mes.MK007399_13243	Mes.gk021416	-	-	1.3	1.4	1.0	0.2	0.1	0.9	0.5	2.7	3.2	0.1	0.1	0.0	0.1	0.5
Mes.MK001950_8871	Mes.gk013241	-	-	0.3	0.1	0.8	1.7	4.7	3.1	0.3	2.1	0.0	0.7	0.6	1.3	0.6	0.9
Mes.MK034616_842	Mes.gk046559	-	-	0.1	0.1	0.0	0.0	0.1	1.9	0.5	0.0	0.2	6.2	2.9	0.5	0.8	1.0
Mes.MK028204_6945	Mes.gk027842	-	-	2.9	5.3	1.1	0.1	0.0	0.2	0.5	1.0	1.4	0.1	1.4	0.2	0.2	0.4
Mes.MK022281_6956	Mes.gk026397	Mes.gk026397	-	0.4	0.9	2.1	0.1	0.1	0.1	0.1	0.0	1.6	2.7	2.8	0.0	2.0	0.0
Mes.MK050906_860	Mes.gk008229	Mes.gk008229	-	1.5	0.0	4.7	0.1	0.6	4.4	0.0	1.0	1.5	0.4	0.1	1.7	0.2	1.0
Mes.MK050906_907	Mes.gk008229	Mes.gk008229	-	1.5	0.0	4.7	0.0	0.3	4.4	0.0	1.0	1.0	0.2	0.3	1.7	0.1	1.0
Mes.MK002465_10478	Mes.gk014168	-	-	0.0	1.8	1.2	0.1	0.0	0.6	2.8	0.0	4.8	0.0	0.1	0.0	0.2	2.6
Mes.MK000927_16502	Mes.gk042026	-	-	2.7	1.2	4.0	0.2	0.9	0.1	0.5	1.5	1.1	1.0	2.1	0.3	1.1	1.7
Mes.MK018627_8589	-	-	Mes.gk025413	0.0	0.4	2.4	1.5	0.5	0.2	1.2	0.4	1.2	0.2	1.1	1.7	3.8	5.7
Mes.MK044352_2573	Mes.gk031711	-	-	0.8	1.6	0.0	0.0	0.2	0.2	1.0	0.1	0.1	0.3	0.1	0.0	3.0	4.3
Mes.MK042282_1127	Mes.gk006822	-	-	0.1	0.5	0.0	0.2	0.4	0.1	0.5	0.7	0.3	4.4	8.0	0.3	1.9	1.0
Mes.MK000696_1217	Mes.gk011589	-	-	0.0	0.0	0.9	0.5	3.7	4.2	0.2	1.2	0.8	0.6	2.2	2.2	0.1	0.5
Mes.MK053475_601	-	-	Mes.gk047944	1.0	0.4	0.0	0.9	1.3	0.6	1.0	0.9	0.6	3.2	4.6	0.1	0.9	1.5
Mes.MK025813_2073	Mes.gk027237	-	-	0.0	0.0	0.0	0.1	0.9	0.8	4.4	4.9	2.2	0.0	0.5	0.5	0.0	0.1
Mes.MK001950_8873	Mes.gk013241	-	-	0.8	0.1	0.9	2.1	6.2	2.9	0.1	2.4	0.0	0.5	0.4	1.1	0.5	1.4
Mes.MK002915_45088	Mes.gk014682	Mes.gk014682	-	0.8	4.6	0.9	0.9	0.1	4.3	1.5	0.0	0.1	0.9	0.4	0.0	0.9	1.6
Mes.MK003025_10465	Mes.gk014822	-	-	0.6	0.1	1.5	0.0	0.7	1.4	0.5	0.5	1.6	3.4	2.1	5.4	1.0	0.1
Mes.MK042438_4855	Mes.gk006850	-	-	2.9	4.5	0.1	0.3	0.0	0.3	1.1	0.5	0.5	0.2	2.1	0.2	0.0	2.2
Mes.MK006474_10461	Mes.gk043958	-	-	0.3	1.2	1.2	0.0	0.0	0.2	0.4	0.0	0.0	3.9	0.5	3.2	0.1	0.2
Mes.MK031712_7341	Mes.gk028817	-	-	2.2	3.6	2.8	0.1	0.0	0.4	1.1	0.2	0.0	2.1	1.8	0.1	0.0	0.8
Mes.MK048473_8837	Mes.gk032762	-	-	0.1	4.0	0.3	0.1	0.3	3.9	0.1	0.2	0.2	0.0	0.1	2.5	1.2	0.5
Mes.MK060366_2740	-	-	Mes.gk049910	4.7	0.1	1.1	2.9	0.1	0.5	0.8	0.4	1.3	0.2	0.1	0.4	0.4	1.2
Mes.MK002216_9740	-	-	Mes.gk000622	3.7	7.6	1.6	1.6	0.0	2.1	0.4	0.4	1.0	0.0	0.1	1.7	0.0	1.0
Mes.MK002216_9784	-	-	Mes.gk000622	3.1	7.1	2.0	1.9	0.0	2.2	0.6	0.4	0.7	0.0	0.2	2.1	0.0	1.4
Mes.MK052445_7953	Mes.gk033653	-	-	6.6	0.3	5.2	0.0	0.1	0.1	1.1	0.5	0.6	1.4	0.1	0.3	0.9	0.3
Mes.MK008679_672	Mes.gk023014	Mes.gk023014	-	0.2	0.2	4.5	0.6	0.5	2.7	1.2	0.5	0.1	0.1	0.6	2.5	0.2	0.4
Mes.MK054405_17244	-	-	Mes.gk048014	1.6	0.3	0.2	3.3	3.1	0.8	0.0	1.6	0.0	0.3	0.0	0.2	0.0	2.0
Mes.MK032048_14037	-	-	Mes.gk028894	0.1	0.0	1.1	2.4	0.7	0.2	0.0	0.6	1.8	4.3	1.9	3.0	1.1	0.6
Mes.MK061771_1600	Mes.gk050366	Mes.gk050366	-	0.2	0.0	0.9	2.3	0.8	2.2	0.0	0.0	0.0	0.1	3.4	3.5	0.0	0.0
Mes.MK008480_3310	Mes.gk022967	-	-	0.6	0.1	0.3	1.3	0.6	1.4	0.0	0.9	0.0	0.4	2.7	3.3	0.6	0.5
Mes.MK063452_8560	Mes.gk010724	-	-	3.4	0.0	0.0	4.4	0.1	0.2	1.5	0.3	0.2	1.8	0.9	0.0	0.3	0.1
Mes.MK002905_7850	Mes.gk000835	-	-	0.5	0.9	0.0	0.1	0.3	2.4	3.1	3.0	0.9	0.0	0.1	1.9	0.1	0.0

Table S10

Genes with repeatable quality and yield QTLs (reQY-QTLGs)

reQY-QTLGs	Gene Region	CDS region	Promoter region	NSR-1	NSR-2	NSR-3	SRY-1	SRY-2	SRY-3	SRY-4	SRY-5	DW-1	DW-2	DW-3	DW-4	FSC-1	FSC-2
Mes.MK013971_4747	Mes.gk002516	Mes.gk002516	-	5.8	3.2	3.5	7.5	0.1	0.1	2.9	0.7	6.2	3.2	0.5	1.7	4.1	1.2
Mes.MK004129_33958	-	-	Mes.gk016223	1.2	5.8	6.1	1.2	5.9	2.5	3.0	3.0	3.6	2.2	0.3	0.0	3.3	0.1
Mes.MK004125_41006	Mes.gk016201	-	-	0.2	2.8	5.9	0.0	4.3	0.0	3.7	3.3	0.2	0.0	3.4	0.9	0.1	0.2
Mes.MK004125_40957	Mes.gk016201	-	-	0.3	4.2	8.8	0.1	4.5	0.0	1.3	4.6	0.0	0.7	4.5	1.3	0.3	0.4
Mes.MK040449_998	Mes.gk030811	Mes.gk030811	-	0.3	6.3	0.4	0.4	0.0	3.0	2.9	0.5	0.0	4.7	1.3	0.5	3.9	0.4
Mes.MK001951_224	-	-	Mes.gk000542	0.2	4.1	0.6	2.8	0.0	3.1	4.3	0.5	1.1	0.0	5.0	2.7	0.0	2.6
Mes.MK004129_34017	Mes.gk016223	Mes.gk016223	-	0.5	4.0	4.9	1.0	3.7	1.8	2.7	2.1	5.0	2.3	0.2	0.0	3.6	0.1
Mes.MK046371_5010	Mes.gk032219	Mes.gk032219	-	0.2	2.8	0.0	0.0	1.1	0.3	3.1	1.0	3.3	4.2	0.8	6.5	1.9	6.3

Mes.MK040711_13182	Mes.gk047006	-	-	3.6	0.1	3.0	1.2	0.0	0.2	0.8	2.7	1.3	3.4	1.1	7.0	3.7	4.2
Mes.MK002915_45089	Mes.gk014682	Mes.gk014682	-	0.3	0.7	5.3	0.4	5.8	3.5	0.3	4.0	5.4	0.0	1.6	1.2	0.1	0.6
Mes.MK000186_3042	Mes.gk010930	Mes.gk010930	-	1.0	2.5	5.4	4.2	0.6	1.5	8.0	1.1	0.1	0.5	0.7	2.7	3.4	4.6
Mes.MK000271_8468	-	-	Mes.gk010995	0.7	3.7	1.8	0.0	1.0	3.4	2.3	3.6	0.1	7.2	0.3	0.0	2.9	0.4
Mes.MK041439_3250	Mes.gk031017	Mes.gk031017	-	0.8	1.7	0.1	3.0	1.6	3.2	1.5	0.0	0.0	3.8	0.0	4.0	4.7	3.6
Mes.MK030223_6213	-	-	Mes.gk028338	7.5	1.3	5.0	3.7	1.6	0.0	0.4	7.5	0.3	0.7	0.0	3.3	2.7	3.5
Mes.MK001729_14850	Mes.gk012973	-	-	2.9	0.0	0.3	2.8	4.7	4.8	0.0	1.6	0.7	2.2	2.1	0.7	3.2	0.9
Mes.MK041219_126	Mes.gk006638	-	-	0.0	0.2	0.0	3.4	0.3	3.0	0.1	0.0	1.3	5.1	1.5	3.2	4.9	6.4
Mes.MK040791_14515	Mes.gk030903	-	-	1.3	3.0	0.5	2.7	0.4	2.9	3.1	0.0	3.7	0.4	0.7	6.9	0.3	5.9
Mes.MK002795_15030	-	-	Mes.gk014569	1.0	4.5	1.4	1.1	0.1	2.9	1.1	0.1	3.5	4.0	0.0	0.1	3.3	0.1
Mes.MK025551_5547	Mes.gk027173	-	-	3.0	2.1	0.1	3.2	0.5	0.0	4.5	0.1	0.8	3.1	0.0	0.8	4.4	0.7
Mes.MK040257_16368	Mes.gk046972	-	-	1.3	4.9	0.8	1.7	3.6	0.9	7.6	0.2	0.0	3.1	0.5	0.1	3.7	0.0
Mes.MK053494_3510	Mes.gk008592	-	-	0.6	1.7	0.8	3.1	0.6	0.5	1.7	3.5	0.1	7.2	0.4	1.6	4.4	2.8
Mes.MK007606_10165	Mes.gk021793	-	-	0.7	0.0	0.0	0.4	0.9	3.0	4.2	0.0	5.6	3.2	3.0	0.0	0.7	0.1
Mes.MK052613_1074	Mes.gk033685	Mes.gk033685	-	3.5	0.2	1.7	0.4	6.8	0.0	0.1	0.0	4.5	3.4	2.2	6.3	0.8	5.4
Mes.MK002795_14839	-	-	Mes.gk014569	3.5	3.0	0.5	3.6	0.0	0.0	0.1	0.1	2.1	5.8	0.0	0.3	3.5	0.1
Mes.MK051000_11037	-	-	Mes.gk047745	0.3	0.8	0.2	0.0	0.5	2.0	3.2	0.8	0.8	8.7	4.8	6.4	6.1	2.3
Mes.MK004967_4042	Mes.gk043273	-	-	0.0	0.0	0.6	1.4	0.4	3.3	2.8	2.2	0.1	3.1	0.1	0.3	2.7	0.3
Mes.MK001955_15368	Mes.gk013244	Mes.gk013244	-	10.	2.1	0.3	4.1	1.5	3.0	0.2	0.7	0.0	0.4	0.2	0.2	2.8	0.0
Mes.MK046094_1775	Mes.gk047398	-	-	3.9	0.7	1.8	3.0	1.4	3.5	1.2	0.7	0.2	0.3	4.2	1.2	0.0	0.7
Mes.MK001377_8160	Mes.gk012502	-	-	0.0	0.9	7.3	0.0	3.2	0.5	3.2	2.9	1.3	1.3	0.0	0.6	0.8	0.7
Mes.MK059203_3592	Mes.gk038174	Mes.gk038174	-	0.0	8.6	0.4	0.6	0.0	0.5	3.0	0.0	1.0	3.0	5.9	0.1	1.8	0.4
Mes.MK004546_444	-	-	Mes.gk001360	1.2	0.3	0.4	0.7	1.1	1.5	0.1	0.3	0.5	5.0	3.9	4.8	4.5	3.8
Mes.MK025967_4841	-	-	Mes.gk045965	0.2	1.0	2.4	0.2	0.5	0.1	0.1	3.0	0.1	4.4	0.0	4.6	3.6	5.1
Mes.MK049198_13143	Mes.gk032950	-	-	0.0	0.4	4.4	0.8	0.2	2.5	3.9	3.0	0.2	0.0	6.2	0.5	0.4	0.2
Mes.MK035925_11780	Mes.gk029797	-	-	3.0	0.4	4.0	0.4	0.2	0.2	0.4	6.1	0.1	0.7	6.2	0.1	0.0	0.1
Mes.MK002648_13145	-	-	Mes.gk014331	2.2	3.6	4.5	0.6	3.0	2.0	0.3	3.1	0.2	0.9	1.5	0.1	0.5	0.6
Mes.MK002795_14976	-	-	Mes.gk014569	1.6	4.5	3.0	0.0	0.2	3.7	1.1	0.4	0.9	4.1	0.2	0.3	2.3	0.3
Mes.MK022395_4606	Mes.gk003794	-	-	0.6	0.0	3.2	3.6	0.1	0.0	0.0	3.6	0.0	0.0	2.4	0.9	0.0	2.9
Mes.MK021844_4002	-	-	Mes.gk026274	1.6	0.1	0.8	3.1	0.1	2.1	0.5	1.2	3.2	4.4	1.3	1.3	7.9	0.5
Mes.MK034683_13922	-	-	Mes.gk046567	0.2	3.8	9.1	0.5	0.0	1.8	2.6	5.3	0.4	0.0	0.3	6.1	0.1	4.3
Mes.MK034683_13904	-	-	Mes.gk046567	0.1	4.1	7.9	0.2	0.0	1.2	2.1	5.2	0.8	0.2	0.5	5.5	0.1	4.2
Mes.MK002156_12337	Mes.gk000599	Mes.gk000599	-	0.6	1.0	0.3	0.3	0.1	4.0	1.4	0.1	0.0	3.7	0.5	4.2	4.8	3.5
Mes.MK020160_2464	Mes.gk025863	-	-	0.0	0.3	5.0	0.0	0.3	2.6	0.0	3.5	0.0	3.2	6.7	0.0	2.2	0.0
Mes.MK043803_232	-	-	Mes.gk007043	0.3	5.3	4.0	2.4	0.0	3.0	1.9	0.9	0.1	0.0	4.0	6.1	0.1	2.4
Mes.MK003518_9729	Mes.gk015419	Mes.gk015419	-	0.0	0.3	2.8	4.9	0.0	0.0	0.2	8.2	0.0	3.1	0.6	0.2	1.0	1.3
Mes.MK045848_8238	Mes.gk047378	-	-	4.1	4.3	1.5	2.2	1.4	1.6	0.4	4.0	0.7	0.8	8.6	6.2	0.0	1.7
Mes.MK001150_2505	Mes.gk042094	-	-	0.0	0.4	0.1	3.2	0.5	5.4	4.4	0.9	1.5	0.1	0.1	4.0	0.1	5.1
Mes.MK046426_5609	Mes.gk032228	Mes.gk032228	-	0.0	0.1	1.6	0.5	1.8	3.0	0.3	0.1	0.1	8.7	3.0	0.0	10.	0.3
Mes.MK045290_1436	Mes.gk031957	Mes.gk031957	-	0.2	0.6	0.3	0.0	2.4	2.0	1.3	0.7	3.5	5.3	0.2	5.1	6.7	4.1
Mes.MK002795_14975	-	-	Mes.gk014569	0.4	3.7	4.9	0.1	0.9	6.2	0.6	1.2	0.6	4.2	0.0	0.1	2.5	0.3
Mes.MK002795_14956	-	-	Mes.gk014569	0.6	3.5	5.6	0.4	0.1	4.6	0.7	2.5	1.4	3.9	0.2	0.2	2.5	0.2
Mes.MK049198_13141	Mes.gk032950	-	-	0.0	0.8	2.9	1.9	0.5	3.5	4.6	2.0	0.0	0.8	5.2	0.2	0.4	0.0
Mes.MK023204_3087	-	-	Mes.gk045782	0.3	5.2	2.2	0.4	4.4	5.2	2.3	0.3	0.7	1.2	0.2	0.5	4.2	0.9
Mes.MK009450_4739	Mes.gk023169	-	-	1.5	0.0	0.0	6.6	0.5	0.8	2.9	0.2	1.1	1.0	3.2	6.6	0.3	6.3
Mes.MK028586_8169	Mes.gk027909	Mes.gk027909	-	2.7	4.2	2.1	0.6	0.0	5.6	0.0	1.1	0.0	1.0	3.9	0.0	0.3	0.2
Mes.MK004225_2223	Mes.gk016354	Mes.gk016354	-	5.9	2.4	0.7	0.0	0.5	7.6	1.9	3.3	1.5	2.2	0.2	0.6	1.9	2.8
Mes.MK002465_10478	Mes.gk014168	-	-	1.6	0.0	3.2	1.2	0.0	0.1	0.5	2.9	4.1	0.3	5.0	0.0	0.3	0.0
Mes.MK002915_45088	Mes.gk014682	Mes.gk014682	-	6.9	0.0	0.3	7.0	0.1	2.5	4.1	0.1	0.9	0.4	6.2	0.3	0.2	0.2
Mes.MK032739_4894	Mes.gk005322	-	-	0.0	3.5	0.1	2.8	1.8	0.0	5.5	0.4	4.3	0.7	0.0	0.0	0.4	0.0
Mes.MK048083_3255	Mes.gk032674	Mes.gk032674	-	0.1	0.8	3.6	0.0	0.0	1.7	1.0	1.3	0.5	3.0	4.9	1.7	3.4	1.7
Mes.MK021153_10615	Mes.gk026073	Mes.gk026073	-	1.7	0.2	3.7	1.5	0.8	0.1	0.0	0.8	0.0	5.2	4.7	1.5	5.6	1.0
Mes.MK045848_8245	Mes.gk047378	-	-	4.2	3.8	1.3	2.0	1.1	1.4	0.2	3.5	0.4	0.5	7.9	7.4	0.0	2.2
Mes.MK019512_2570	-	-	Mes.gk045474	0.9	0.0	0.7	0.5	2.0	4.7	2.7	0.0	1.9	6.1	0.5	0.2	2.9	0.0
Mes.MK046315_12192	Mes.gk032197	-	-	0.4	0.1	0.0	0.2	0.0	5.6	4.5	0.2	0.0	6.0	1.9	0.0	4.3	0.2
Mes.MK061053_4184	Mes.gk010151	Mes.gk010151	-	0.4	3.1	2.2	0.1	1.5	0.8	4.5	3.4	2.4	3.1	1.2	0.8	1.6	1.5
Mes.MK030223_6156	-	-	Mes.gk028338	5.7	0.1	4.2	1.9	2.0	0.3	1.0	6.6	1.1	1.1	0.7	2.8	4.6	2.4
Mes.MK030522_8708	Mes.gk028398	Mes.gk028398	-	0.3	2.9	1.9	0.2	1.0	2.4	3.1	4.2	0.0	4.8	0.0	2.2	2.3	1.3
Mes.MK026630_2398	Mes.gk027473	-	-	0.2	3.9	0.4	1.3	1.1	1.5	2.1	0.5	4.3	8.7	0.3	0.4	7.7	0.3
Mes.MK024659_5646	Mes.gk026954	-	-	2.8	2.0	5.9	0.8	0.9	0.2	2.9	4.6	0.5	0.1	1.3	0.3	0.4	2.3
Mes.MK022281_6933	Mes.gk026397	Mes.gk026397	-	3.3	0.1	0.0	2.7	2.0	0.2	0.0	0.7	2.2	4.3	4.4	0.4	3.1	0.1

Mes.MK033273_5589	Mes.gk029165	-	-	1.6	0.2	0.3	0.9	0.7	0.5	0.5	0.5	4.9	3.6	5.6	1.7	3.0	1.3
Mes.MK047203_4015	-	-	Mes.gk032431	5.6	2.8	0.5	2.7	7.9	0.2	0.8	1.1	0.1	0.5	0.7	0.3	0.2	0.0
Mes.MK001090_15908	Mes.gk000322	-	-	3.2	0.9	2.9	4.7	0.8	3.7	1.7	1.2	0.2	0.1	0.0	0.1	0.3	0.2
Mes.MK026386_6514	-	-	Mes.gk027384	4.7	0.5	0.4	6.3	2.0	0.1	0.8	0.0	3.4	2.0	3.2	0.1	0.2	0.3
Mes.MK056198_10253	Mes.gk009064	-	-	0.1	0.1	1.8	0.4	0.1	0.0	3.9	0.4	0.0	4.6	0.1	4.6	4.1	3.6
Mes.MK043374_638	Mes.gk047207	Mes.gk047207	-	0.0	4.1	4.4	1.2	1.8	0.2	0.1	3.3	0.4	0.9	1.4	3.3	1.0	3.1
Mes.MK014301_186	Mes.gk024416	-	-	0.3	3.6	1.5	1.2	2.8	2.0	6.3	2.4	0.0	0.1	0.1	4.4	0.3	6.2
Mes.MK025633_973	-	-	Mes.gk004309	2.2	4.5	3.5	0.0	1.0	0.0	8.7	1.9	0.2	1.0	5.0	0.1	0.2	0.9
Mes.MK025633_947	-	-	Mes.gk004309	2.5	1.1	4.1	0.2	0.7	1.1	3.7	3.6	0.0	0.1	4.5	0.5	0.1	1.5
Mes.MK003255_24474	Mes.gk015101	-	-	0.2	0.4	0.2	0.0	0.3	3.6	1.4	0.0	0.7	2.8	0.1	1.0	3.0	3.8
Mes.MK057695_6326	Mes.gk048988	-	-	0.0	0.1	0.9	2.9	0.1	0.7	0.0	2.8	1.4	6.1	0.2	0.6	2.8	0.1
Mes.MK059329_699	Mes.gk049563	Mes.gk049563	-	3.4	0.0	3.4	3.6	3.5	0.8	2.4	0.9	0.0	0.0	0.0	0.2	0.4	1.2
Mes.MK033273_5451	Mes.gk029165	-	-	2.4	0.2	0.1	2.9	5.1	2.8	0.4	0.0	1.1	1.6	4.1	0.1	0.5	0.1
Mes.MK044228_974	Mes.gk047271	Mes.gk047271	-	1.8	0.3	0.4	5.2	4.3	3.4	0.8	0.1	0.1	0.2	3.5	0.7	0.0	0.7
Mes.MK060740_16388	-	-	Mes.gk039488	0.4	0.0	0.0	0.6	1.4	0.0	1.1	0.2	3.4	4.0	4.5	0.3	7.2	0.7
Mes.MK003268_4618	-	-	Mes.gk000950	4.6	1.1	0.1	4.0	0.4	6.1	0.7	0.0	4.5	0.2	0.7	1.5	0.3	1.7
Mes.MK044967_2125	-	-	Mes.gk007244	0.4	0.2	0.1	1.0	0.3	0.4	1.1	0.0	0.3	3.5	3.3	2.2	4.3	0.5
Mes.MK026645_1030	-	-	Mes.gk004452	1.9	0.1	3.3	0.9	0.5	4.0	0.5	2.8	0.0	0.7	0.4	0.2	0.8	1.2
Mes.MK000106_50561	-	-	Mes.gk010835	0.7	2.0	0.2	0.0	0.9	1.1	3.4	2.9	1.1	1.0	9.5	1.4	1.6	0.4
Mes.MK003518_9727	Mes.gk015419	Mes.gk015419	-	0.0	1.1	1.2	5.2	0.1	0.1	0.8	4.7	0.0	5.0	1.0	0.2	2.1	0.1
Mes.MK046315_12193	Mes.gk032197	-	-	0.3	0.0	0.6	3.3	0.0	7.4	2.8	0.2	0.0	1.8	0.9	0.0	2.1	0.1
Mes.MK018779_10068	Mes.gk025472	-	-	3.0	1.0	0.6	4.1	4.8	0.0	0.1	1.1	0.2	0.5	2.0	0.3	0.0	0.3
Mes.MK055035_5763	Mes.gk008822	Mes.gk008822	-	0.8	0.5	6.7	0.0	1.4	3.0	0.0	4.7	0.0	1.6	2.2	0.1	0.8	0.0
Mes.MK055035_5739	Mes.gk008822	Mes.gk008822	-	1.1	0.7	8.4	0.1	2.2	3.4	0.0	6.1	0.0	1.3	2.0	0.0	0.4	0.0
Mes.MK002152_432	Mes.gk013520	-	-	0.3	1.4	9.5	0.7	3.1	0.7	2.6	5.5	1.0	1.6	1.5	0.1	2.0	0.4
Mes.MK042131_2570	Mes.gk031189	-	-	0.1	1.7	0.1	0.0	0.0	1.0	0.1	0.9	0.1	3.7	0.0	4.3	3.3	4.5
Mes.MK049470_6540	Mes.gk033006	Mes.gk033006	-	3.5	0.7	4.7	0.8	0.1	0.3	0.3	2.9	0.0	0.1	2.5	0.2	0.5	0.2
Mes.MK024436_601	-	-	Mes.gk004109	0.0	1.8	6.4	0.6	0.1	0.7	3.0	6.4	0.1	1.9	0.0	0.5	1.9	0.0
Mes.MK002836_30416	-	-	Mes.gk014606	0.9	0.5	2.7	0.0	12.	0.4	0.4	4.6	0.6	2.7	0.6	0.0	2.5	0.4
Mes.MK033753_7825	-	-	Mes.gk029282	0.2	0.0	4.2	0.3	2.7	0.3	0.0	3.0	0.2	1.4	0.1	0.7	1.6	0.0
Mes.MK004460_59961	Mes.gk016661	-	-	0.0	8.7	2.8	0.0	0.0	0.0	0.0	4.1	0.7	2.0	0.6	0.1	2.1	0.0
Mes.MK030449_9572	-	-	Mes.gk028378	2.5	1.4	0.0	0.4	3.2	0.2	2.9	0.2	1.0	2.5	0.1	0.4	5.8	0.3
Mes.MK031243_8727	Mes.gk028617	-	-	0.3	0.4	2.5	0.1	0.1	8.5	4.2	0.6	4.2	2.7	0.0	0.4	1.6	0.5
Mes.MK000544_4662	Mes.gk011401	-	-	0.1	0.3	0.3	3.5	2.8	0.2	1.3	0.0	0.5	3.2	0.9	0.3	1.9	0.1
Mes.MK000365_36150	Mes.gk011147	-	-	2.9	0.4	4.2	0.4	0.0	1.4	0.1	4.2	0.7	0.1	0.5	0.0	0.7	0.0
Mes.MK017898_2113	Mes.gk025221	Mes.gk025221	-	1.2	0.0	0.9	2.9	4.2	4.5	0.0	1.8	2.1	1.1	0.2	0.8	0.7	0.0
Mes.MK017898_2132	Mes.gk025221	Mes.gk025221	-	1.2	0.0	0.9	2.9	4.2	4.5	0.0	1.8	2.1	1.1	0.2	0.8	0.7	0.0
Mes.MK017898_2200	Mes.gk025221	-	-	1.2	0.0	0.8	2.9	4.2	4.7	0.0	1.7	2.1	1.2	0.2	0.8	0.7	0.0
Mes.MK000400_5218	-	-	Mes.gk011215	1.6	2.0	2.1	3.5	0.2	2.9	2.4	2.0	3.5	1.3	1.2	4.1	0.3	2.1
Mes.MK003168_17809	-	-	Mes.gk014985	0.1	0.9	1.3	3.1	1.8	4.7	3.4	1.1	0.0	0.1	0.1	0.9	0.1	0.0
Mes.MK036406_1167	-	-	Mes.gk046686	0.0	1.1	1.4	3.2	0.1	1.2	4.6	6.2	0.7	0.1	1.6	0.0	0.1	0.5
Mes.MK034732_1547	Mes.gk029530	-	-	3.2	0.1	0.0	4.6	0.1	4.6	0.9	0.3	0.5	0.1	0.7	0.7	0.2	0.3
Mes.MK002246_13008	-	-	Mes.gk000633	0.7	0.7	1.7	3.4	0.7	9.4	0.3	1.9	3.8	0.4	2.0	0.0	0.2	0.7
Mes.MK004469_11779	Mes.gk016693	-	-	0.4	0.6	4.2	0.3	0.2	0.0	4.7	7.5	2.5	0.1	0.0	0.0	0.4	0.2
Mes.MK010661_3428	Mes.gk023474	-	-	0.2	0.8	0.1	1.5	2.2	1.3	0.1	0.5	0.1	3.5	2.9	3.3	1.2	3.5
Mes.MK010661_3407	Mes.gk023474	-	-	0.2	1.2	0.1	1.5	1.7	2.0	0.2	0.2	0.1	3.5	3.4	4.6	1.2	4.9
Mes.MK006776_19276	Mes.gk020247	-	-	0.2	0.5	1.6	1.5	5.3	3.6	0.2	2.8	0.5	2.1	0.0	1.3	0.0	1.1
Mes.MK011913_949	Mes.gk002166	Mes.gk002166	-	2.5	0.4	0.3	0.0	0.3	0.7	0.8	0.0	0.7	7.5	0.5	6.0	5.6	5.9
Mes.MK004346_22362	Mes.gk016551	Mes.gk016551	-	0.1	2.2	0.0	0.4	0.0	0.1	0.0	0.0	0.1	2.8	5.2	1.0	3.3	0.4
Mes.MK032476_2008	Mes.gk005284	Mes.gk005284	-	0.1	0.7	2.6	0.0	0.3	0.7	1.3	1.2	1.0	4.4	0.6	7.2	5.8	3.1
Mes.MK024764_7579	Mes.gk026994	-	-	5.4	1.5	1.5	1.5	0.1	3.9	0.1	4.7	0.9	0.0	1.6	0.4	0.0	0.0
Mes.MK031244_8226	Mes.gk028620	-	-	0.5	3.2	0.2	0.1	0.3	6.9	3.4	0.3	0.2	1.0	1.5	0.6	0.0	1.0
Mes.MK031244_8228	Mes.gk028620	-	-	0.5	3.2	0.3	0.1	0.4	7.1	3.4	0.4	0.2	0.9	1.7	0.4	0.0	1.0
Mes.MK046426_5603	Mes.gk032228	Mes.gk032228	-	0.3	0.2	0.3	0.3	1.2	1.4	0.4	0.0	0.4	4.5	5.1	0.0	4.8	0.1
Mes.MK046426_5612	Mes.gk032228	Mes.gk032228	-	0.4	0.2	0.2	0.1	1.9	1.7	0.0	0.2	0.0	5.3	3.2	0.0	5.5	0.1
Mes.MK059793_4762	Mes.gk038653	-	-	2.5	0.3	5.1	3.6	0.0	0.0	0.2	4.2	0.1	0.0	0.4	0.6	0.0	0.5
Mes.MK007940_6770	Mes.gk022409	Mes.gk022409	-	0.0	5.3	0.5	0.5	0.1	3.5	3.3	1.7	0.6	0.5	0.6	0.2	0.3	0.2
Mes.MK003859_13593	Mes.gk043006	Mes.gk043006	-	0.5	0.0	0.2	0.6	1.2	0.7	0.0	0.8	0.8	3.5	4.1	0.2	3.9	0.0
Mes.MK034616_842	Mes.gk046559	-	-	0.1	0.3	0.7	5.2	0.4	4.9	3.6	0.0	0.2	0.0	1.0	2.8	0.1	1.2
Mes.MK022900_7715	Mes.gk045754	-	-	1.7	0.0	0.2	1.8	0.0	0.1	0.6	0.1	1.5	4.0	3.6	0.2	3.1	0.3
Mes.MK003130_5901	-	-	Mes.gk014932	1.0	4.7	0.0	1.1	4.1	0.2	3.0	0.0	0.3	0.1	0.5	0.5	0.3	0.6

Mes.MK028204_6945	Mes.gk027842	-	-	0.3	0.0	1.5	0.2	0.2	1.0	0.5	0.0	0.0	2.9	5.4	0.2	4.2	0.4
Mes.MK022281_6956	Mes.gk026397	Mes.gk026397	-	0.5	0.2	0.7	0.9	0.2	0.7	0.5	0.0	0.7	3.5	3.1	0.1	3.4	0.2
Mes.MK049659_2078	Mes.gk033039	-	-	0.0	0.4	0.2	0.0	1.3	1.2	0.0	0.0	0.7	2.7	3.1	0.2	6.8	0.7
Mes.MK059329_610	-	-	Mes.gk049563	3.0	0.0	3.1	2.5	3.1	0.4	2.0	0.8	0.1	0.0	0.0	0.2	0.3	1.5
Mes.MK031329_995	Mes.gk028648	Mes.gk028648	-	0.8	4.5	0.1	1.3	5.0	0.4	2.8	0.0	0.5	0.0	1.1	0.4	0.0	0.4
Mes.MK000029_38039	Mes.gk010750	-	-	0.0	4.9	2.0	0.8	0.0	1.1	4.1	2.9	0.0	0.1	0.4	1.0	0.3	1.1
Mes.MK017922_9756	-	-	Mes.gk025231	2.1	1.5	0.0	0.5	0.1	3.8	0.1	0.0	6.4	0.4	3.7	1.1	0.0	0.2
Mes.MK049217_3792	Mes.gk032956	Mes.gk032956	-	1.3	0.0	1.5	2.8	5.4	0.3	0.4	2.5	0.5	0.0	0.0	0.8	1.6	3.3
Mes.MK029736_8287	-	-	Mes.gk028185	3.4	0.2	0.9	6.0	0.0	5.0	0.5	1.0	0.7	0.3	0.3	3.0	0.2	1.8
Mes.MK000108_16687	Mes.gk010841	Mes.gk010841	-	0.0	1.8	2.4	3.7	1.8	0.1	0.2	0.9	0.1	4.1	2.7	0.7	1.7	0.6
Mes.MK044753_2192	Mes.gk031831	Mes.gk031831	-	0.4	1.0	0.8	0.1	1.5	6.1	7.1	0.7	4.9	0.3	1.1	0.0	0.1	0.0
Mes.MK030701_5138	Mes.gk028441	Mes.gk028441	-	0.2	0.7	0.6	0.4	0.5	0.0	0.0	0.0	7.8	2.3	2.7	0.0	2.9	0.1
Mes.MK014716_2959	Mes.gk002617	-	-	1.1	3.4	1.0	4.0	0.2	0.1	7.6	1.1	0.7	0.1	0.0	0.0	0.2	0.1
Mes.MK018406_1634	Mes.gk003153	-	-	0.0	0.3	1.0	3.2	0.2	3.2	1.7	1.5	1.7	0.2	3.9	2.0	0.2	0.7
Mes.MK002022_3597	-	-	Mes.gk013321	0.0	0.8	0.9	2.9	0.5	4.8	0.4	1.0	5.5	1.9	0.0	0.1	1.4	0.3
Mes.MK009463_5368	Mes.gk023180	Mes.gk023180	-	1.3	0.9	0.0	4.3	0.1	0.1	1.3	0.1	3.4	0.1	4.6	0.0	0.3	0.0
Mes.MK050443_1163	Mes.gk033177	Mes.gk033177	-	4.6	1.6	0.2	7.3	0.0	1.8	3.4	0.6	0.0	1.4	0.1	1.3	0.9	1.2
Mes.MK003400_6980	Mes.gk015249	-	-	1.8	0.2	3.5	2.0	0.0	3.1	0.0	3.5	0.1	0.6	0.5	0.4	0.3	1.5
Mes.MK003694_1707	Mes.gk042938	Mes.gk042938	-	1.2	0.1	1.2	0.3	1.9	0.3	0.0	0.2	0.1	3.2	3.6	3.6	0.3	3.4
Mes.MK012483_2701	Mes.gk023976	-	-	0.0	2.9	0.1	0.0	0.1	0.8	1.9	0.1	1.4	2.1	0.2	3.3	2.9	4.5
Mes.MK030755_6691	Mes.gk046315	Mes.gk046315	-	0.1	0.7	1.0	0.0	0.8	4.9	3.2	1.0	1.7	3.8	1.2	1.1	2.5	0.4
Mes.MK035444_31519	-	-	Mes.gk029692	1.8	0.1	4.3	1.5	1.6	3.0	0.8	2.8	0.2	0.6	2.2	0.0	2.1	0.8
Mes.MK014431_12841	Mes.gk045094	-	-	0.9	0.7	4.9	3.1	2.4	1.3	0.8	3.3	0.6	0.0	1.3	3.2	0.5	1.8
Mes.MK055282_12611	Mes.gk008878	-	-	2.2	0.5	0.7	3.6	0.2	0.4	3.1	1.1	0.3	0.8	0.5	6.1	2.1	5.4
Mes.MK000670_2596	-	-	Mes.gk011555	0.7	0.0	3.4	0.4	2.8	0.1	0.8	3.3	2.1	0.0	0.3	0.9	0.5	0.0
Mes.MK040257_16215	Mes.gk046972	-	-	1.3	3.1	0.4	1.7	3.3	2.4	4.8	0.0	0.0	1.6	0.4	0.1	2.3	0.2
Mes.MK038563_4432	Mes.gk006204	-	-	1.0	2.1	5.7	1.1	0.0	3.1	0.6	3.3	1.3	0.0	1.3	2.4	0.1	2.0
Mes.MK005553_13737	-	-	Mes.gk018095	0.8	1.0	0.3	0.0	0.5	1.0	0.0	0.9	0.1	7.1	3.6	0.7	5.8	0.7
Mes.MK022889_12190	Mes.gk026556	-	-	0.0	1.3	0.0	0.4	0.9	1.2	0.5	0.1	0.0	4.5	2.9	0.0	3.7	0.1
Mes.MK005722_3621	Mes.gk018416	Mes.gk018416	-	0.4	0.0	0.2	0.1	0.2	1.5	0.0	0.0	0.2	4.4	4.4	0.0	4.8	0.0
Mes.MK024932_900	Mes.gk027031	-	-	0.0	1.1	1.8	0.9	0.4	2.7	6.8	2.5	1.1	1.7	0.4	0.0	2.8	0.1
Mes.MK059633_1441	Mes.gk009875	-	-	0.1	1.3	1.7	0.3	0.1	0.1	2.4	0.0	2.3	3.3	0.9	1.9	7.9	3.0
Mes.MK000106_56979	-	-	Mes.gk010836	0.5	0.0	0.1	3.1	1.1	5.2	2.9	0.2	0.1	0.1	0.7	0.0	0.0	0.0
Mes.MK053129_10265	Mes.gk033798	-	-	0.7	0.3	1.1	3.2	2.8	0.2	0.9	0.0	0.7	0.7	3.3	1.4	0.0	2.1
Mes.MK007399_13241	Mes.gk021416	-	-	3.6	0.2	3.1	0.1	3.1	0.0	1.7	0.7	1.2	1.9	0.4	0.4	2.2	0.5
Mes.MK026575_6852	Mes.gk004441	Mes.gk004441	-	2.7	0.0	3.4	0.1	0.0	0.8	0.1	0.9	4.3	0.0	0.3	0.3	0.1	0.1
Mes.MK052085_3526	Mes.gk033575	Mes.gk033575	-	0.0	0.9	0.1	1.4	0.0	4.5	3.6	2.0	4.9	1.2	0.0	0.4	0.5	1.8
Mes.MK052085_3528	Mes.gk033575	Mes.gk033575	-	0.0	0.9	0.1	1.5	0.0	4.7	3.8	2.0	4.4	1.4	0.0	0.3	0.5	1.8
Mes.MK001000_6841	-	-	Mes.gk011997	0.8	0.6	0.6	6.5	4.4	4.6	0.1	1.3	0.1	0.0	0.7	1.7	0.1	1.5
Mes.MK032739_4891	Mes.gk005322	-	-	0.1	0.9	0.6	0.1	2.8	0.2	2.8	0.2	3.1	0.5	0.1	0.0	0.0	0.7
Mes.MK013933_4358	Mes.gk002505	Mes.gk002505	-	1.5	3.0	0.0	3.2	1.3	0.3	8.7	0.1	0.3	0.4	2.7	1.0	0.0	0.7
Mes.MK039490_342	Mes.gk046917	Mes.gk046917	-	3.1	4.2	3.5	0.1	1.8	1.6	0.7	0.0	1.8	0.0	0.5	0.0	1.1	0.4
Mes.MK038529_2481	Mes.gk006197	-	-	2.5	0.9	0.3	3.8	1.0	2.9	2.9	1.7	0.1	0.4	0.2	0.7	1.9	0.7
Mes.MK025610_2282	Mes.gk027187	-	-	2.2	1.6	0.7	1.0	0.0	1.9	3.7	0.1	2.8	0.6	4.6	0.1	1.4	1.3
Mes.MK003350_12815	Mes.gk042838	-	-	3.1	2.9	0.4	1.2	2.6	0.5	2.7	0.2	0.3	0.1	2.4	1.8	0.0	1.7
Mes.MK056364_4616	Mes.gk035448	Mes.gk035448	-	3.2	0.5	0.1	4.0	0.9	0.9	3.1	0.7	0.0	0.8	0.6	2.3	0.1	1.5
Mes.MK031508_25345	-	-	Mes.gk028752	3.2	0.1	0.0	5.0	0.0	0.0	7.8	0.3	1.0	0.2	0.2	0.4	0.1	0.0
Mes.MK021932_8128	Mes.gk003717	Mes.gk003717	-	2.7	0.0	0.5	3.0	0.2	4.1	1.3	1.0	0.0	2.4	0.7	1.6	0.3	1.0
Mes.MK021320_15598	Mes.gk045606	-	-	3.0	2.3	1.8	5.5	0.4	0.6	3.2	0.7	2.0	0.3	0.8	0.2	0.8	1.6
Mes.MK037190_337	Mes.gk030085	Mes.gk030085	-	3.6	0.2	0.2	4.1	0.0	1.4	5.5	0.6	0.7	0.0	0.0	0.1	0.1	0.0
Mes.MK009557_7219	Mes.gk044714	-	-	5.7	0.0	0.3	8.1	4.8	2.1	0.3	1.1	1.6	0.1	2.4	1.2	0.1	1.8
Mes.MK009592_315	Mes.gk001770	Mes.gk001770	-	3.5	0.1	0.0	3.9	4.1	0.4	0.7	0.2	0.4	0.7	0.6	2.4	0.1	2.4
Mes.MK046646_1031	-	-	Mes.gk007559	2.9	1.2	0.7	4.1	0.2	0.4	4.4	0.0	0.3	0.5	1.1	1.2	0.5	0.8
Mes.MK015832_9758	-	-	Mes.gk045198	3.4	2.7	0.1	3.2	0.6	0.6	2.1	0.0	0.2	0.2	2.6	1.0	0.2	0.0
Mes.MK000442_352	Mes.gk000142	Mes.gk000142	-	0.8	0.0	0.5	0.0	0.0	1.6	0.1	0.3	0.0	2.8	0.2	3.2	3.4	2.7
Mes.MK026144_4004	Mes.gk004385	-	-	0.1	0.2	2.7	0.4	0.4	3.8	0.2	2.8	0.3	3.2	0.9	0.0	1.4	0.1
Mes.MK048377_3748	-	-	Mes.gk047562	1.4	0.0	0.9	0.5	1.1	0.1	0.4	0.0	0.0	4.1	1.1	2.9	5.2	3.7
Mes.MK051363_3694	Mes.gk008290	Mes.gk008290	-	0.0	0.2	0.0	0.4	0.4	0.0	0.7	0.2	0.9	2.8	0.0	1.0	5.6	3.0
Mes.MK008747_4217	Mes.gk044644	-	-	1.8	2.9	2.9	0.2	1.7	1.0	0.0	2.9	2.4	1.1	2.4	0.6	0.0	0.0
Mes.MK001270_6185	-	-	Mes.gk012360	4.2	1.5	3.9	1.5	0.0	0.0	0.2	1.4	0.2	3.6	0.2	0.9	2.6	1.1
Mes.MK047898_1291	Mes.gk032612	-	-	2.6	0.8	1.2	0.9	0.2	4.6	2.9	0.8	0.0	2.3	3.7	0.2	2.4	0.0

Mes.MK022889_12177	Mes.gk026556	-	-	0.7	0.0	0.5	0.2	0.7	0.2	0.0	0.1	0.1	7.0	3.9	0.1	7.8	0.2
Mes.MK023049_7143	Mes.gk045772	Mes.gk045772	-	1.1	0.1	0.0	1.3	0.0	0.0	0.0	0.3	0.4	3.0	3.3	0.0	3.0	0.4
Mes.MK022889_12193	Mes.gk026556	-	-	0.1	0.2	0.4	0.5	0.8	1.4	0.6	0.3	0.6	4.9	5.5	0.0	5.7	0.0
Mes.MK009766_5878	Mes.gk001803	Mes.gk001803	-	2.0	0.3	0.0	1.7	0.1	0.8	0.1	0.1	0.1	3.5	6.5	0.8	3.6	0.7
Mes.MK053989_3153	Mes.gk008661	Mes.gk008661	-	0.0	0.3	0.2	0.9	2.0	0.0	0.4	1.0	0.4	2.8	0.1	1.4	3.3	3.0
Mes.MK039784_1121	-	-	Mes.gk006405	1.0	0.1	0.5	0.4	1.9	0.1	0.7	0.4	0.7	6.4	1.1	1.7	8.1	4.0
Mes.MK022406_6013	Mes.gk003796	-	-	0.0	0.1	2.7	0.0	0.6	0.5	1.0	0.5	6.0	2.9	2.8	0.1	2.0	0.0
Mes.MK048922_9318	Mes.gk032877	Mes.gk032877	-	3.1	5.0	0.0	2.4	0.3	0.9	4.1	1.1	0.4	0.0	0.7	0.0	0.1	0.4
Mes.MK048035_20400	Mes.gk047541	-	-	0.0	2.3	0.4	4.2	0.0	0.1	2.8	1.0	0.3	3.1	0.4	0.1	0.1	0.1
Mes.MK002213_12517	-	-	Mes.gk042443	1.0	1.7	0.2	1.2	2.1	0.0	4.9	0.0	4.7	3.5	1.2	0.0	2.2	0.2
Mes.MK025610_2284	Mes.gk027187	-	-	1.4	1.3	0.7	0.8	0.0	1.0	4.3	0.3	4.3	0.2	3.3	0.0	0.5	0.5
Mes.MK011913_1078	Mes.gk002166	Mes.gk002166	-	1.7	0.0	0.3	7.2	6.1	0.0	0.0	0.1	4.2	0.3	2.4	1.5	0.1	2.0
Mes.MK012494_3739	-	-	Mes.gk023983	3.5	0.4	1.0	11.	4.3	0.1	0.1	2.1	0.0	0.1	0.2	1.6	0.1	1.6
Mes.MK058604_1302	Mes.gk009625	-	-	0.3	0.0	1.4	2.8	2.5	0.1	1.8	0.8	6.1	4.5	0.9	0.1	1.3	0.4
Mes.MK000984_8265	Mes.gk011975	Mes.gk011975	-	3.4	1.8	1.7	5.1	1.5	0.3	3.8	0.2	0.8	0.3	0.0	0.4	0.2	0.8
Mes.MK034495_19625	Mes.gk029453	-	-	0.1	1.8	0.3	2.4	2.9	4.8	0.0	0.5	0.3	1.4	0.0	0.2	0.5	0.0
Mes.MK002317_62142	Mes.gk013867	-	-	0.1	1.4	0.0	8.5	0.2	4.4	0.6	0.2	1.3	0.9	0.1	6.0	0.1	2.5
Mes.MK016755_1831	Mes.gk002931	-	-	0.9	0.5	0.4	0.4	5.2	4.5	0.0	0.1	0.1	0.3	2.1	0.5	0.6	0.7
Mes.MK030630_3597	-	-	Mes.gk028419	0.5	0.0	1.6	0.4	2.9	7.9	0.5	0.2	0.7	0.9	0.0	1.6	0.0	0.5
Mes.MK031712_7339	Mes.gk028817	-	-	0.2	3.7	4.1	0.3	0.0	0.3	0.1	2.3	0.3	1.0	0.2	0.0	0.3	0.0
Mes.MK043690_256	Mes.gk007020	-	-	1.4	0.3	0.3	4.9	5.0	0.0	0.0	1.2	0.1	0.5	0.2	0.1	0.6	0.0
Mes.MK003698_12381	-	-	Mes.gk001091	0.1	0.8	0.0	0.0	3.5	0.0	3.2	1.1	1.3	0.0	1.2	1.1	0.0	0.7
Mes.MK003698_12400	-	-	Mes.gk001091	0.1	0.8	0.0	0.0	3.5	0.0	3.2	1.1	1.3	0.0	1.2	1.1	0.0	0.7
Mes.MK049202_9179	Mes.gk032952	-	-	1.0	0.9	0.4	2.6	3.8	0.1	0.1	3.0	0.4	0.0	1.1	0.1	0.1	1.7
Mes.MK041406_4770	Mes.gk031011	Mes.gk031011	-	0.0	0.0	0.4	5.7	0.0	5.4	0.3	0.4	2.5	0.0	2.3	4.1	0.3	1.8
Mes.MK025967_4838	-	-	Mes.gk045965	2.2	0.4	0.6	0.4	2.6	0.0	0.0	0.5	0.0	2.5	0.0	1.5	4.0	2.8
Mes.MK003480_10959	-	-	Mes.gk015350	1.0	1.6	0.0	3.0	1.7	4.2	1.9	1.1	2.0	0.0	0.6	0.0	0.2	0.0
Mes.MK003152_7243	-	-	Mes.gk014951	1.3	0.9	1.0	3.4	3.6	0.3	0.0	0.1	1.3	1.1	1.7	0.4	0.8	0.0
Mes.MK027638_4387	Mes.gk004598	-	-	0.9	0.0	1.6	0.2	0.0	5.9	1.4	2.8	0.0	0.7	2.6	0.3	1.2	0.0
Mes.MK016665_3983	Mes.gk024927	Mes.gk024927	-	2.4	2.2	1.4	0.7	3.3	6.0	1.6	0.2	0.2	0.5	2.5	0.0	2.3	0.2
Mes.MK057937_1823	Mes.gk009440	Mes.gk009440	-	1.0	0.3	2.3	3.8	0.0	0.1	1.6	3.9	0.6	0.1	0.4	0.3	0.0	0.4
Mes.MK032839_5033	-	-	Mes.gk046462	2.6	0.0	0.1	1.4	0.3	2.9	3.0	0.8	0.0	0.4	0.8	1.7	0.0	1.6
Mes.MK030630_3580	-	-	Mes.gk028419	1.1	0.0	1.9	0.8	3.8	4.5	1.7	0.1	0.1	0.3	0.4	0.4	1.2	0.2
Mes.MK030630_3553	-	-	Mes.gk028419	1.3	0.4	1.6	2.4	4.8	8.7	0.1	0.3	1.3	1.4	0.9	0.6	0.6	0.0
Mes.MK048295_5867	Mes.gk047557	Mes.gk047557	-	1.0	0.8	1.1	3.3	0.0	0.0	0.1	3.1	0.5	1.8	0.3	0.6	0.8	0.4
Mes.MK037209_1777	-	-	Mes.gk006013	0.7	0.4	1.8	0.9	1.5	3.7	1.7	3.1	0.7	1.1	0.3	0.9	1.7	0.0
Mes.MK002088_5918	Mes.gk013414	-	-	1.8	1.8	0.1	0.0	1.3	0.4	0.4	0.0	0.7	1.8	2.0	0.7	3.1	2.9
Mes.MK000872_5132	Mes.gk000266	-	-	0.0	2.3	0.0	1.5	13.	3.2	0.4	0.0	2.5	0.1	0.1	0.0	0.3	0.3
Mes.MK003856_3324	Mes.gk015887	-	-	0.3	0.6	0.8	0.9	0.1	3.2	3.7	0.3	0.6	0.0	1.3	0.0	0.0	0.0
Mes.MK004550_6550	-	-	Mes.gk043208	0.5	1.9	2.5	0.5	0.0	0.2	4.3	3.1	0.2	0.8	0.4	2.1	1.5	0.5
Mes.MK031712_7341	Mes.gk028817	-	-	0.0	4.2	4.4	1.0	0.0	0.4	0.4	2.6	1.3	1.1	0.1	0.0	0.5	0.1
Mes.MK000108_14250	-	-	Mes.gk010841	0.7	0.7	0.5	3.8	0.3	3.6	0.7	0.7	1.7	1.5	0.8	4.0	0.4	2.6
Mes.MK008679_672	Mes.gk023014	Mes.gk023014	-	0.0	1.3	0.0	0.0	0.2	0.0	0.1	0.7	0.1	2.0	0.0	2.3	5.6	4.0
Mes.MK002149_5286	-	-	Mes.gk013513	0.0	0.0	2.4	2.9	0.1	3.8	1.1	0.3	0.2	0.4	0.0	0.1	0.1	0.5
Mes.MK011023_5327	-	-	Mes.gk023540	0.1	0.0	1.1	3.1	0.0	0.0	0.9	5.0	0.1	0.0	0.1	0.0	0.1	0.0
Mes.MK005814_4695	Mes.gk018603	-	-	0.6	0.0	0.1	5.5	3.4	2.3	0.3	0.5	0.3	0.5	2.5	0.0	0.1	0.7
Mes.MK051100_1099	Mes.gk008251	Mes.gk008251	-	0.3	2.3	2.2	4.6	0.1	2.8	0.5	1.1	1.7	0.2	0.9	0.5	0.0	0.6
Mes.MK045139_6256	Mes.gk047319	Mes.gk047319	-	0.2	0.4	0.4	2.7	2.0	1.4	0.2	0.1	4.0	3.0	1.5	0.1	0.1	0.0
Mes.MK001800_3223	Mes.gk000513	Mes.gk000513	-	0.9	0.2	0.3	3.9	0.8	1.7	0.3	3.2	0.2	0.5	1.7	0.3	0.4	0.3
Mes.MK001365_3658	Mes.gk000397	Mes.gk000397	-	0.0	0.3	0.2	3.7	1.2	0.2	0.0	4.0	0.3	0.0	0.4	1.0	0.2	1.4
Mes.MK000683_8040	Mes.gk011576	-	-	1.5	1.0	0.1	3.6	1.7	0.1	3.2	1.3	1.0	0.1	0.5	0.1	0.3	0.4
Mes.MK033104_1619	-	-	Mes.gk029121	0.1	1.4	0.6	4.4	0.1	0.0	2.7	0.3	2.4	0.7	0.0	1.1	0.0	0.4
Mes.MK012503_2953	-	-	Mes.gk023984	1.2	0.2	0.9	5.8	0.1	5.4	0.7	1.1	2.7	0.0	1.4	0.2	0.0	0.2
Mes.MK003263_5407	-	-	Mes.gk015116	0.8	0.5	0.9	5.0	0.1	3.1	2.4	2.5	2.2	0.2	1.7	2.6	0.1	1.0
Mes.MK002524_2834	Mes.gk042563	-	-	1.0	0.9	0.3	5.1	0.3	0.1	6.9	0.2	0.1	0.2	0.4	0.2	0.1	0.1
Mes.MK003700_5885	Mes.gk042944	Mes.gk042944	-	0.2	2.1	0.4	5.5	0.3	0.5	4.4	0.1	1.3	0.8	0.9	0.2	0.0	0.2
Mes.MK044825_3228	-	-	Mes.gk047308	2.5	0.1	0.6	0.8	2.6	0.2	0.0	0.7	3.0	1.3	2.9	0.1	0.6	0.3
Mes.MK030461_3892	-	-	Mes.gk005020	2.6	0.0	0.0	0.1	1.9	0.3	0.1	0.4	2.9	2.9	0.0	0.1	1.9	0.3
Mes.MK021844_4007	-	-	Mes.gk026274	0.8	0.5	0.7	2.9	6.2	0.4	0.2	0.9	1.6	0.0	0.1	0.5	0.8	0.2
Mes.MK058430_9688	Mes.gk009573	Mes.gk009573	-	1.4	0.1	1.0	0.5	2.8	2.9	0.6	0.4	0.1	0.0	0.8	3.5	0.2	2.1
Mes.MK032890_3726	Mes.gk029076	-	-	0.0	1.1	0.9	0.0	0.9	5.4	2.8	1.6	0.3	0.0	0.5	0.0	0.1	0.0

Mes.MK035019_5660	-	-	Mes.gk005668	0.9	0.0	0.4	1.2	2.7	1.3	0.5	0.6	0.3	0.7	0.9	2.9	3.3	2.8
Mes.MK003428_2030	Mes.gk000999	Mes.gk000999	-	1.0	0.6	1.7	0.1	5.0	1.1	3.5	1.4	0.4	0.5	0.6	0.7	0.1	0.1
Mes.MK018627_8466	-	-	Mes.gk025413	0.0	0.0	1.0	0.3	4.9	0.1	1.4	3.7	0.4	0.3	1.4	1.2	0.4	0.6
Mes.MK035043_1243	Mes.gk005673	Mes.gk005673	-	3.8	0.1	3.8	0.4	0.1	1.1	0.3	2.6	0.0	0.1	0.7	0.2	0.0	0.2
Mes.MK026659_3302	Mes.gk004454	Mes.gk004454	-	0.0	0.1	1.4	0.0	0.1	2.9	4.7	1.4	0.0	1.8	0.0	0.2	0.3	0.0
Mes.MK001171_11307	Mes.gk012242	Mes.gk012242	-	1.8	0.7	2.1	1.4	2.5	3.4	2.8	2.2	1.7	0.7	0.5	0.0	0.2	0.0
Mes.MK028675_10522	-	-	Mes.gk046143	0.2	0.0	0.3	1.4	4.6	1.1	3.0	0.1	0.2	1.9	0.0	0.0	0.9	0.0
Mes.MK003428_2029	Mes.gk000999	Mes.gk000999	-	0.7	0.0	0.0	0.1	7.1	2.0	4.0	0.1	0.0	0.0	0.6	0.4	0.1	0.0
Mes.MK048574_4405	Mes.gk047575	-	-	1.1	2.9	2.8	0.7	1.3	0.0	0.1	0.8	0.3	1.6	1.3	0.0	1.0	0.1
Mes.MK000104_1152	-	-	Mes.gk000021	0.3	0.3	1.9	0.2	1.3	2.8	0.1	3.8	2.1	0.4	0.7	0.0	0.9	0.2
Mes.MK020304_6158	Mes.gk003478	-	-	0.0	1.0	2.4	0.0	0.0	0.6	2.9	4.1	0.7	0.1	1.5	1.7	2.7	0.0
Mes.MK019823_2398	-	-	Mes.gk025765	0.0	0.8	1.4	0.3	0.6	4.7	0.0	3.2	1.1	0.1	0.6	0.2	0.1	0.0
Mes.MK024659_5666	Mes.gk026954	-	-	0.1	1.1	0.7	0.0	0.6	0.0	0.8	0.1	0.7	1.7	0.2	1.0	3.2	3.4
Mes.MK014965_5905	-	-	Mes.gk024559	0.3	0.5	0.1	2.6	4.9	0.6	3.1	0.2	0.1	0.1	2.4	0.0	0.5	0.1
Mes.MK014153_3757	Mes.gk045066	-	-	1.6	0.6	1.0	2.8	0.6	3.2	1.5	1.0	1.2	0.8	0.2	0.8	1.9	0.2
Mes.MK002149_5201	-	-	Mes.gk013513	0.0	0.0	2.1	2.8	0.1	4.1	1.2	0.1	0.1	0.3	0.0	0.1	0.2	0.4
Mes.MK042467_5296	Mes.gk006854	Mes.gk006854	-	1.5	2.1	0.4	3.8	1.1	0.0	0.0	2.7	0.1	0.2	0.0	0.3	1.8	0.0
Mes.MK047772_7247	Mes.gk032575	Mes.gk032575	-	2.5	0.1	0.0	3.6	2.8	2.7	0.2	0.0	0.0	2.1	1.0	0.0	2.4	0.2
Mes.MK000379_3692	Mes.gk011178	Mes.gk011178	-	1.3	0.3	0.4	3.6	3.8	2.2	0.1	0.4	0.3	0.2	0.4	0.0	0.6	0.4
Mes.MK021765_4094	-	-	Mes.gk026245	2.7	0.9	0.8	4.5	0.7	0.1	7.6	1.3	0.3	1.8	0.4	0.4	1.9	0.0
Mes.MK004304_4858	Mes.gk001251	Mes.gk001251	-	0.1	0.0	1.2	2.8	0.6	3.2	0.1	0.4	0.8	0.0	0.4	0.1	0.0	0.0
Mes.MK002488_3321	Mes.gk000701	Mes.gk000701	-	0.2	0.1	0.1	0.1	0.9	0.5	0.2	0.6	4.3	0.8	3.0	0.1	1.7	0.1
Mes.MK039587_9002	Mes.gk030642	Mes.gk030642	-	0.1	0.2	0.6	1.4	0.0	0.7	1.1	0.3	7.3	0.1	3.2	0.6	0.2	1.0
Mes.MK039587_9201	Mes.gk030642	Mes.gk030642	-	0.0	0.0	0.5	0.0	0.0	1.1	1.0	0.6	3.4	0.0	7.1	2.5	0.0	2.7
Mes.MK013570_647	-	-	Mes.gk024201	0.8	0.9	0.6	0.0	0.2	0.7	1.5	1.7	3.0	5.3	0.1	0.0	2.0	0.0
Mes.MK020085_12027	Mes.gk025847	Mes.gk025847	-	0.2	0.2	0.6	0.3	0.3	0.6	1.1	1.2	3.6	0.2	3.2	1.3	0.1	1.1
Mes.MK002013_5527	-	-	Mes.gk013315	2.3	0.1	1.5	0.2	0.9	0.6	0.5	0.8	6.2	3.3	1.0	1.6	1.1	0.6
Mes.MK002013_5535	-	-	Mes.gk013315	2.5	0.0	1.6	0.2	0.9	0.5	0.5	0.8	6.1	3.2	1.1	1.4	1.2	0.5
Mes.MK062759_1263	-	-	Mes.gk050737	0.4	1.0	1.2	0.2	0.1	0.1	0.0	0.0	3.9	3.3	0.0	0.8	1.4	0.6
Mes.MK041445_138	-	-	Mes.gk031020	3.1	5.2	0.1	0.3	0.8	0.2	2.5	0.3	0.2	1.5	0.6	0.7	1.6	0.4
Mes.MK031382_75156	Mes.gk028703	Mes.gk028703	-	2.4	0.0	0.1	6.7	0.4	3.1	0.2	1.4	0.5	0.1	1.9	0.4	0.3	0.8
Mes.MK003702_11155	Mes.gk015653	Mes.gk015653	-	2.0	0.6	0.0	3.8	0.8	0.0	2.8	0.4	2.3	0.9	1.6	0.1	0.3	0.8
Mes.MK026555_978	Mes.gk004439	Mes.gk004439	-	1.6	1.5	0.0	5.1	1.7	0.1	3.7	0.1	1.9	0.0	0.7	0.2	0.1	0.3
Mes.MK026555_944	Mes.gk004439	Mes.gk004439	-	2.1	1.6	0.6	5.6	1.0	0.1	4.2	0.1	2.2	0.2	0.8	0.3	0.1	0.4
Mes.MK030913_4327	Mes.gk028538	Mes.gk028538	-	0.0	0.0	0.2	3.7	0.6	0.1	2.7	0.0	1.9	0.0	0.7	0.0	0.3	0.6
Mes.MK036499_2938	Mes.gk046694	Mes.gk046694	-	0.4	1.7	0.2	3.2	0.2	0.7	3.2	1.2	0.1	0.6	1.5	0.0	0.1	0.0
Mes.MK005427_14115	Mes.gk017837	Mes.gk017837	-	0.0	0.5	1.2	0.3	0.9	0.1	0.2	0.0	0.8	2.1	1.4	7.4	6.4	6.5
Mes.MK035019_5690	-	-	Mes.gk005668	0.9	0.0	0.4	1.2	2.7	1.3	0.5	0.6	0.3	0.7	0.9	2.9	3.3	2.8
Mes.MK056371_19133	Mes.gk009097	Mes.gk009097	-	0.1	0.2	0.1	3.2	4.5	0.4	0.3	0.6	0.1	0.0	0.0	3.0	0.1	1.4
Mes.MK000104_1270	-	-	Mes.gk000021	0.3	0.2	2.4	0.2	1.7	3.2	0.1	4.2	2.1	0.3	1.1	0.2	0.9	0.7
Mes.MK000104_1207	-	-	Mes.gk000021	0.3	0.2	2.4	0.2	1.7	3.2	0.1	4.2	2.1	0.3	1.1	0.2	0.9	0.7
Mes.MK031593_6179	Mes.gk028786	-	-	0.0	1.1	0.5	0.0	4.4	3.6	1.1	1.0	2.5	2.0	2.0	0.0	0.3	0.1
Mes.MK023337_5018	Mes.gk026659	-	-	0.1	2.3	0.2	0.2	4.1	0.9	2.8	0.3	0.2	0.1	0.5	0.6	0.1	0.7
Mes.MK035161_8650	Mes.gk029628	Mes.gk029628	-	0.6	1.7	1.3	0.9	0.5	1.6	2.9	2.7	0.0	0.7	1.4	0.4	0.6	0.8
Mes.MK022394_6576	Mes.gk026426	Mes.gk026426	-	0.1	0.0	0.1	0.1	0.2	2.1	1.6	0.0	1.6	2.3	2.7	2.5	2.8	2.8
Mes.MK029550_10745	Mes.gk028146	Mes.gk028146	-	0.6	0.2	0.5	0.1	0.1	3.6	0.2	4.4	0.3	0.5	0.9	0.3	0.1	0.1
Mes.MK000106_57313	-	-	Mes.gk010836	1.3	0.0	0.2	3.4	0.4	3.7	2.0	0.0	0.0	0.4	0.8	0.0	0.1	0.0
Mes.MK021443_6081	Mes.gk045619	-	-	2.9	3.2	0.5	1.0	0.0	0.3	2.5	0.0	0.3	0.1	1.6	0.1	0.0	1.3
Mes.MK027899_39	-	-	Mes.gk004646	0.2	0.4	0.4	2.9	1.1	5.1	0.3	0.0	0.3	0.8	0.0	1.2	1.5	1.5

Captions for additional tables

Table S3

Annotation of monomorphic fully methylation in population, with BGI WEGO (P<0.001, χ^2 text), compared with cassava KU50 genes[14]

Table S5

QTL^{epi} results in KS population

Table S8

Genetic analysis of cassava, including SNVs, QTLs and annotations
